# The Clock and Wavefront Self-Organizing model recreates the dynamics of mouse somitogenesis *in vivo* and *in vitro*

**DOI:** 10.1242/dev.202606

**Published:** 2024-05-16

**Authors:** Julie Klepstad, Luciano Marcon

**Affiliations:** Andalusian Center for Developmental Biology (CABD) CSIC-UPO-JA, Carretera de Utrera km 1, 41013 Seville, Spain

**Keywords:** Mathematical model, Self-organization, Reaction-diffusion, Explants, Phase waves, Excitability

## Abstract

During mouse development, presomitic mesoderm cells synchronize Wnt and Notch oscillations, creating sequential phase waves that pattern somites. Traditional somitogenesis models attribute phase waves to a global modulation of the oscillation frequency. However, increasing evidence suggests that they could arise in a self-organizing manner. Here, we introduce the Sevilletor, a novel reaction-diffusion system that serves as a framework to compare different somitogenesis patterning hypotheses. Using this framework, we propose the Clock and Wavefront Self-Organizing model that considers an excitable self-organizing region where phase waves form independent of global frequency gradients. The model recapitulates the change in relative phase of Wnt and Notch observed during mouse somitogenesis and provides a theoretical basis for understanding the excitability of mouse presomitic mesoderm cells *in vitro*.

## Teaser

**Highlighted Article:** A theoretical framework named Sevilletor shows that the clock and wavefront model with a new self-organizing region can recapitulate the oscillations and excitability observed during mouse somitogenesis.

## INTRODUCTION

**Fig. 1.**
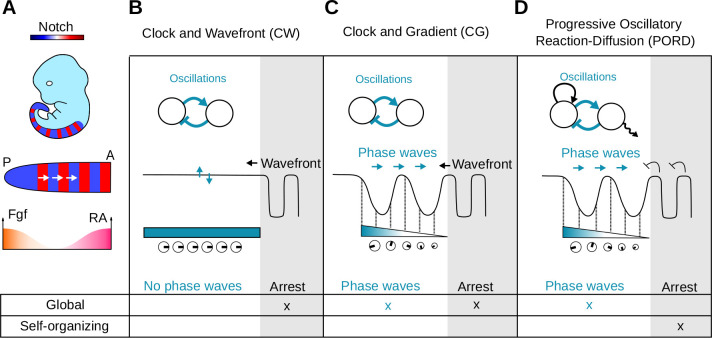
**Previous models of somitogenesis.** (A) During somitogenesis, coordinated genetic oscillations give rise to phase waves traversing the embryo from the posterior (P) to the anterior (A) side, orchestrated by signals like Fgf and retinoic acid (RA). (B-D) Previous somitogenesis models show oscillations emerging from delayed negative feedback (blue arrows) modulated by global frequency profiles (blue/white regions and clocks) or local cell communication, influencing the formation and arrest of phase waves. (B) The Clock and Wavefront model (Cooke and Zeeman, 1976) features homogeneous phase oscillations regulated by a global determination front. (C) The Clock and Gradient model (Morelli et al., 2009; Herrgen et al., 2010) exhibits phase waves due to a monotonically decreasing frequency profile. (D) The Progressive Oscillatory Reaction-Diffusion model (Cotterell et al., 2015) forms phase waves with a global frequency profile and arrest oscillations in a self-organizing manner.

During embryonic development, precise coordination of cell behaviors in both time and space is essential to generate robust gene expression patterns at the tissue level. A remarkable example of this coordination can be observed during somitogenesis (Miao and Pourquié, 2024), the formation of body segment precursors. In vertebrates, this process is characterized by waves of gene expression that propagate from the posterior tip of the tail to the anterior side, resulting in the sequential formation of somites (Fig. 1A). These waves are generated by synchronizing oscillations driven by the segmentation clock, a genetic network of presomitic mesoderm (PSM) cells that pattern somites in a rhythmic manner. Previous studies have demonstrated that the core of the segmentation clock is implemented by delayed negative feedbacks (Lewis, 2003; Takashima et al., 2011) and that the waves arise from a phase shift of the clock along the anterior-posterior axis (Masamizu et al., 2006; Aulehla et al., 2008). The specific mechanism responsible for the spatial synchronization of oscillations at the tissue level, however, remains a subject of debate.

The Clock and Wavefront (CW) model (Cooke and Zeeman, 1976) was the first theoretical study to suggest that oscillation in the posterior part of the PSM (clock) controls somitogenesis. According to this model, when cells exit this posterior region they enter a determination front (wavefront) that could be defined by global positional information signals (Wolpert, 1969) undergoing rapid changes governed by the phase of the clock to form periodic somite patterns (Fig. 1B). The main ideas of the CW model have received strong experimental support in chick, zebrafish and mouse, where it has been shown that the posterior PSM exhibits homogeneous oscillations of Notch, Wnt and Fgf signaling (Aulehla and Pourquié, 2010; Oates, 2020; Palmeirim et al., 1997). Moreover, experimental evidence shows that posterior signaling gradients of Wnt and Fgf can modulate the oscillations at the posterior tip of the tail (Dubrulle et al., 2001; Naiche et al., 2011; Aulehla and Pourquié, 2010), while retinoic acid (RA) forms an anterior gradient that localizes within newly formed somites and promotes differentiation (Duester, 2007; Aulehla and Pourquié, 2010; Oates, 2020) (Fig. 1A).

In its original formulation, however, the CW model fails to account for the formation of phase waves observed during vertebrate somitogenesis. A popular reincarnation of the CW model that addresses this issue is the Clock and Gradient model (CG), which assumes that the frequency of the clock slows down gradually from posterior to anterior (Morelli et al., 2009; Herrgen et al., 2010) promoting a spatial alternation of phases (Fig. 1C). Adding local coupling to the CG model enhances robustness and scales the overall frequency (Morelli et al., 2009; Herrgen et al., 2010) but the formation of wave patterns in these models is promoted by the frequency profile (Oates et al., 2012). One possibility is that the tissue-wide frequency profile is regulated by posterior signals, for example by modulating the local coupling-strength of Delta-Notch (Kuyyamudi et al., 2022). Genetic manipulations in mice, however, have shown that multiple phase waves can form even with the ectopic activation of posterior signals throughout the entire PSM (Aulehla et al., 2008), suggesting that the formation of phase waves may exhibit a degree of self-organization.

On one hand, this spatial self-organization could occur in a cell-autonomous manner, as recent experiments indicate that isolated cells of the zebrafish PSM *in vitro* slow down and stop oscillations depending on their anterior-posterior position (Rohde et al., 2021 preprint). On the other hand, the synchronization of oscillation underlying phase waves may involve local cell communication (Maroto et al., 2005; Masamizu et al., 2006; Webb et al., 2016; Oates, 2020). This is especially relevant in mouse, where it has been shown that isolated PSM cells stop oscillating *in vitro* and can be excited to oscillate by increasing cell density (Hubaud et al., 2017), showing that, in mouse, cell communication plays a central role in both initiating and synchronizing oscillations at the tissue level (Newman, 2022).

The capacity of the mouse PSM to spontaneously synchronize oscillations and generate phase waves has also been highlighted with tail explants *in vitro* (Lauschke et al., 2013; Hubaud et al., 2017; Tsiairis and Aulehla, 2016). These explants can generate circular phase waves that resemble somitogenesis upon significant cellular re-arrangement and perturbation of embryonic signals. Wave formation has also been observed in more heterogeneous cultures obtained by mixing cells from different tailbud explants (Hubaud et al., 2017; Tsiairis and Aulehla, 2016), highlighting the ability of the PSM to self-organize. The idea that PSM can self-organize has also been explored in the Progressive Oscillatory Reaction-Diffusion (PORD) model (Cotterell et al., 2015), in which new somites form through a relay mechanism triggered by the last formed somite via local cell communication (Fig. 1D). In this model, however, phase waves are still formed by a global frequency profile gradient similar to the CG model. Therefore, the role that self-organization plays in phase wave formation remains unclear.

Here, we present a novel theoretical framework for studying spatial synchronization of oscillations via self-organization or graded frequency profiles. Our framework includes a core reaction-diffusion system driven by delayed negative feedback between two self-enhancing genes, a regionalizing function and a graded frequency function. This model can generate diverse self-organizing behaviors at the tissue level including lateral inhibition, rotating waves and periodic wave patterns. These are common behaviors of systems far from equilibrium (Cross and Hohenberg, 1993; Tyson and Keener, 1988) and have been observed in previous reaction-diffusion models (Shepelev and Vadivasova, 2019; Singh and Sinha, 2013). One of the strengths of our framework lies in its capacity to generate this range of self-organizing behaviors by adjusting a single reaction parameter that promotes a bifurcation. Additionally, near this bifurcation, our system exhibits a previously undescribed dynamic behavior: the formation of periodic phase waves via diffusion-driven excitation of a bistable state. These phase waves resemble patterns observed in models of the Belousov–Zhabotinsky chemical reaction (Zhabotinsky and Zaikin, 1973; Prigogine and Lefever, 1968; Nicolis and Prigogine, 1977; Field and Noyes, 1974), such as the Brusselator and the Oregonator, from Brussels (Belgium) and Oregon (USA), respectively. Continuing the tradition, we named our theoretical framework the Sevilletor, as it was developed in Seville (Spain).

We demonstrate that the Sevilletor framework can recapitulate the main qualitative patterning behaviors of the principal theoretical models proposed to explain somitogenesis with minimal parameter changes. This allows us to directly compare the qualitative behavior of different models. Moreover, it leads us to devise a new somitogenesis model that extends the CW model with an excitable self-organizing region where phase waves form independent of global frequency gradients. We name this model the Clock and Wavefront Self-Organizing model (CWS), and show that it provides a theoretical basis for understanding the excitability of mouse PSM cells observed *in vitro* (Hubaud et al., 2017). Notably, the addition of this excitable region can also explain the change in the relative phase between Notch and Wnt observed in the middle part of the mouse tail (Aulehla et al., 2003; Sonnen et al., 2018). Overall, we show that the CWS model can recapitulate the self-organizing potential of the PSM both *in vivo* and *in vitro*.

## RESULTS

We begin the results section by providing a summary of a detailed theoretical analysis presented in the Materials and Methods, where we use complex systems theory and numerical simulations to introduce and characterize a new reaction-diffusion system called Sevilletor. The rest of the result section focuses on the application of this system to study the qualitative behaviors of previous somitogenesis models and to propose a novel somitogenesis model where phase waves are formed with an excitable behavior.

### Summary of the theoretical analysis of the Sevilletor system

**Fig. 2.**
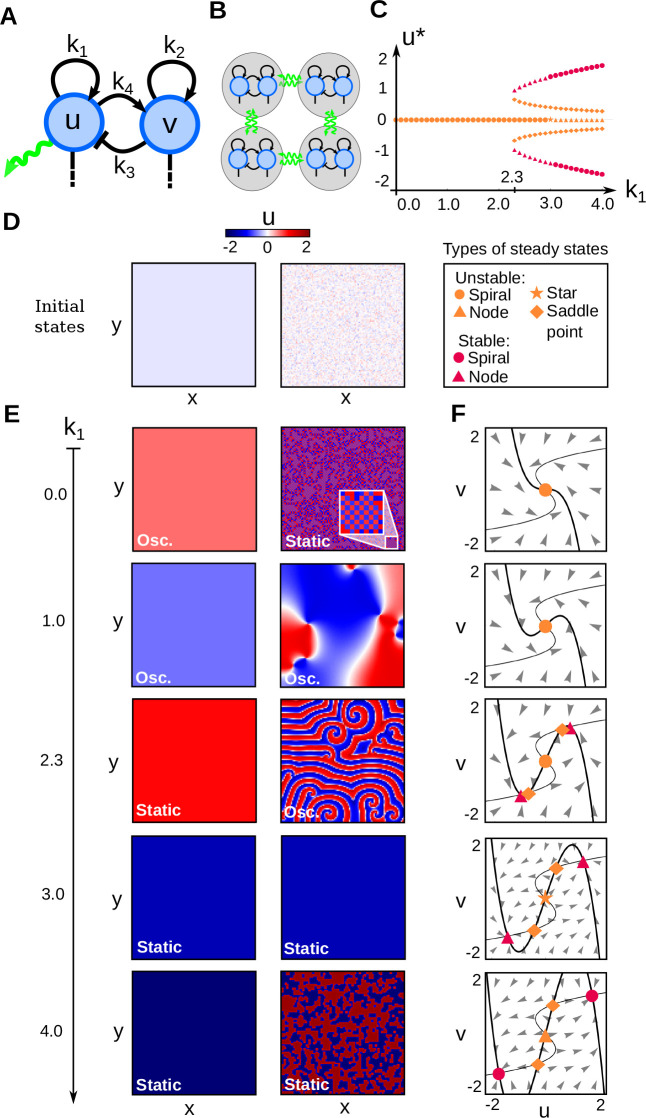
**Patterning behavior and bifurcations in the Sevilletor equations.** (A) The Sevilletor equations couple self-enhancing reactants ***u*** and ***v*** with negative feedback. *k*_1_ determines the strength of ***u*** self-enhancement (*k*_2,3,4_=1). (B) Diffusion of ***u*** influences neighboring cell behaviors in a 2D grid (green arrows). (C) Bifurcation diagram for *k*_1_ in the Sevilletor equations, showing steady state values of ***u***. (D) Initial ***u*** conditions: homogeneous on the left, with noise on the right. The size of the systems are *L*_***x***_=*L*_***y***_=100 and ***D***=0.3. (E) Two-dimensional simulations with increasing *k*_1_: left column shows homogeneous oscillatory or static patterns, right column shows lateral inhibition, rotating waves, spirals, homogeneous patterns, and salt and pepper bistable patterns (Movie 1). (F) Phase spaces with increasing *k*_1_: nullclines of ***u*** (thick line) and ***v*** (thin line), steady states (red and orange markers) and vector field direction (arrowheads). The nullcline of ***u*** changes shape with increasing values of *k*_1_, undergoing a bifurcation at *k*_1_=2.3 gaining two stable states (red markers) near to two unstable states (orange markers). All parameters are shown in Tables S1 and S2 in Supplementary Section S23.

We devised a minimal equation system called Sevilletor that couples two self-enhancing reactants *u* and *v* with a negative feedback, and limits the deviation of concentrations with cubic saturation terms, see Fig. 2A and Eqns 1 and 2, introduced in detail in the Materials and Methods. On the one hand, the negative feedback between the two reactants (*k*_3_ and *k*_4_) in the model promotes sustained oscillations like the one generated by single reactant models with a delayed negative feedback. On the other hand, varying the relative self-enhancement strength of *u* and *v* (*k*_1_ and *k*_2_) promotes a bifurcation from an oscillatory state around an unstable point to a bistable state (Fig. 2A-C), adding two additional stable points close to two additional unstable points (Fig. 2F; Fig. S4). This differs from the bifurcations seen in classical models of wave formation (FitzHugh, 1961; Cross and Hohenberg, 1993; Tyson and Keener, 1988), where the bistable regime is typically characterized by a central unstable and two stable points (Fig. S3C).
(1)

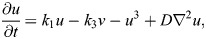

(2)

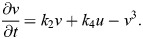


**Fig. 3.**
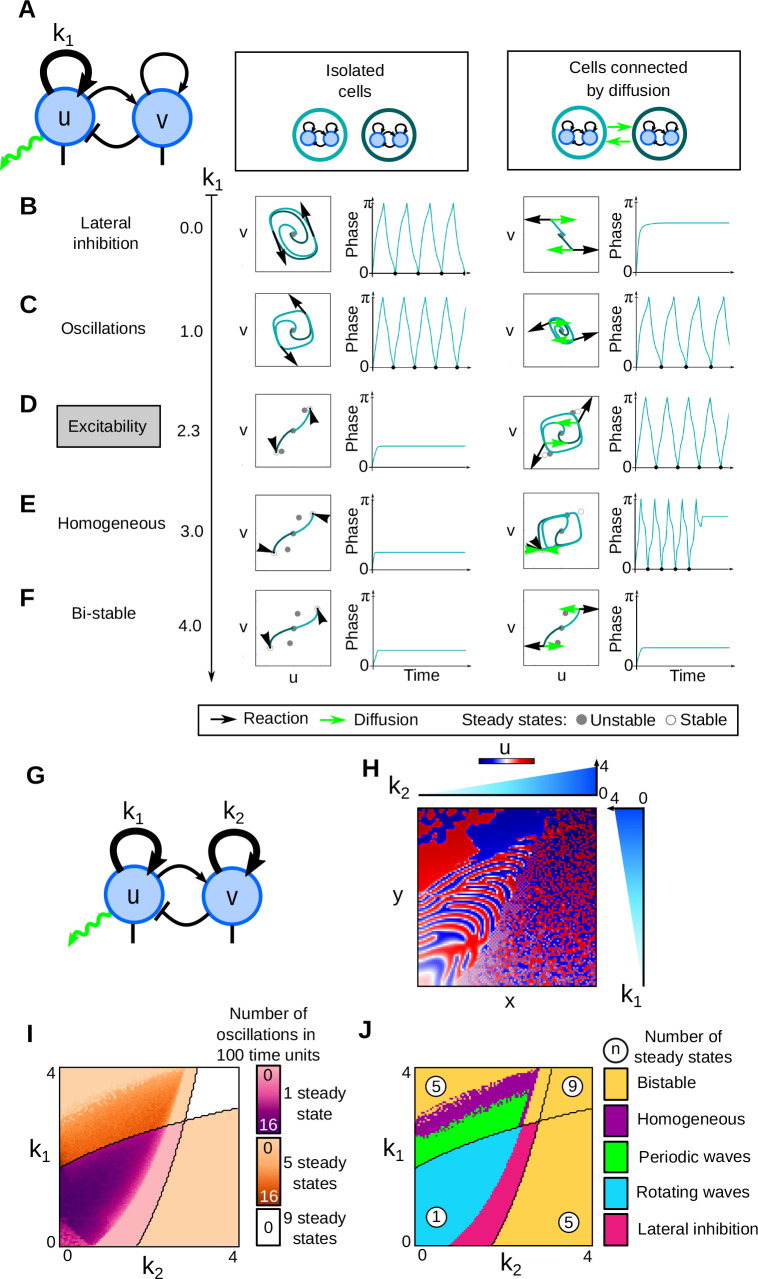
**Effect of *k*_1_, *k*_2_ and diffusion in the Sevilletor equations.** (A-F) Varying *k*_1_ induces different behaviors in two cells with opposite phases, with and without diffusion (***D***=0.3 and ***D***=0). Initial conditions: (***u***, ***v***)=(−0.1, 0) in first cell (dark teal) and (***u***, ***v***)=(0.1, 0) in second cell (light teal). *k*_3_=*k*_4_=1. Left: trajectory of two cells in phase space; right: phase of one cell over time (calculation shown in Fig. 6). Black and green arrows indicate reaction and diffusion contributions, respectively. (B) With *k*_1_=0, isolated cells oscillate but stop when diffusion counterbalances reaction (Movie 2). (C) With *k*_1_=1, cells oscillate with and without diffusion. (D) With *k*_1_=2.3, isolated cells are in a bistable regime but can excite each other to oscillate when coupled by diffusion, generating a new limit cycle (Movie 3; Supplementary Section S3). (E) With *k*_1_=3, isolated cells are in a bistable regime but can synchronize oscillations and eventually stop when coupled by diffusion. (F) With *k*_1_=4, both isolated and coupled cells are in a bistable regime. (G-J) Bifurcation diagrams for *k*_1_ and *k*_2_ with associated numerical simulations. (H) Two-dimensional simulation with graded *k*_1_ along the *y*-axis and *k*_2_ along the *x*-axis recapitulates all patterning behaviors (Movie 5). *L*_***x***_=*L*_***y***_=100 and ***D***=0.3. (I,J) Number of oscillations quantified in two-cell simulations for different *k*_1_ and *k*_2_ values extend the bifurcation diagram (graded colors in I), showing different patterning regimes promoted by diffusion (colored regions in J). Black lines in I and J depict bifurcations without diffusion. All parameters are shown in Tables S1 and S2 in Supplementary Section S23.

In addition, we observed that for different self-enhancement strengths, diffusion of *u* between cells can give rise to five different patterning behaviors from noise: lateral inhibition patterns, rotating wave patterns, periodic wave patterns with spiral formation, homogeneous patterns and bistable frozen patterns (right column in Fig. 2E, Fig. 3G-J; Movie 1). An analysis with two-cell simulations revealed that diffusion plays a different role in each of these scenarios, as it can be seen by comparing the trajectories with and without diffusion in phase space (Fig. 3A-F; Movies 2 and 3). It can be either stabilizing (lateral inhibition) to freeze the oscillations of neighboring cells in opposite phase generating chessboard patterns, synchronizing to generate rotating waves, or destabilizing. In the latter case, cells are in a default bistable state and diffusion excites neighboring cells in opposite phase to generate periodic phase waves (Fig. 3D; Movie 3). A convenient feature of the Sevilletor model is that a progressive change in the strength of *k*_1_ in the model not only promotes a bifurcation from oscillation to bistability but it also controls the distance between stable and unstable points within the bistability regime (Fig. 2F), which determines the excitability of the system. When this distance is small then diffusion can easily excite the bistable state, whereas a larger distance decreases the impact that diffusion has on the dynamics, lowering the excitability of the system. A similar phase portrait was presented in Jutras-Dubé et al. (2020) and François et al. (2007), however this novel diffusion-driven excitable behavior has not been described previously. This behavior differs from excitability in classical models such as the FitzHugh-Nagumo (FitzHugh, 1961) and the Complex Ginzburg-Landau (Tyson and Keener, 1988; Aranson and Kramer, 2002), where excitatory dynamics typically emerge from destabilizing a single stable point with a large stimulus, triggering a temporary deviation from the equilibrium state of the system (Fig. S3B) (Supplementary Section S3). In addition, this behavior differs from a classical Turing instability (Turing, 1952) (Fig. S2; Supplementary Section S2).

In the following section, we show that the different dynamical behaviors are amenable to simulate the main patterning hypotheses proposed to study mouse somitogenesis.

### Sevilletor as a framework to study somitogenesis

Our goal is to compare the qualitative patterning behaviors of different somitogenesis models by exploiting the dynamical patterning regimes exhibited by the Sevilletor equations. Within this context, our objective is to capture the qualitative aspects that control the emergence of oscillations, phase waves, and the arrest of oscillations in different models, rather than replicating the quantitative details of each scenario. To do so, we modified the Sevilletor equations by adding two spatial functions – R (regions) and FG (frequency gradient) – that promote distinct modulations along the anterior-posterior axis of the developing tail:
(3)

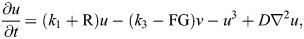

(4)

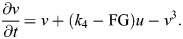


**Fig. 4.**
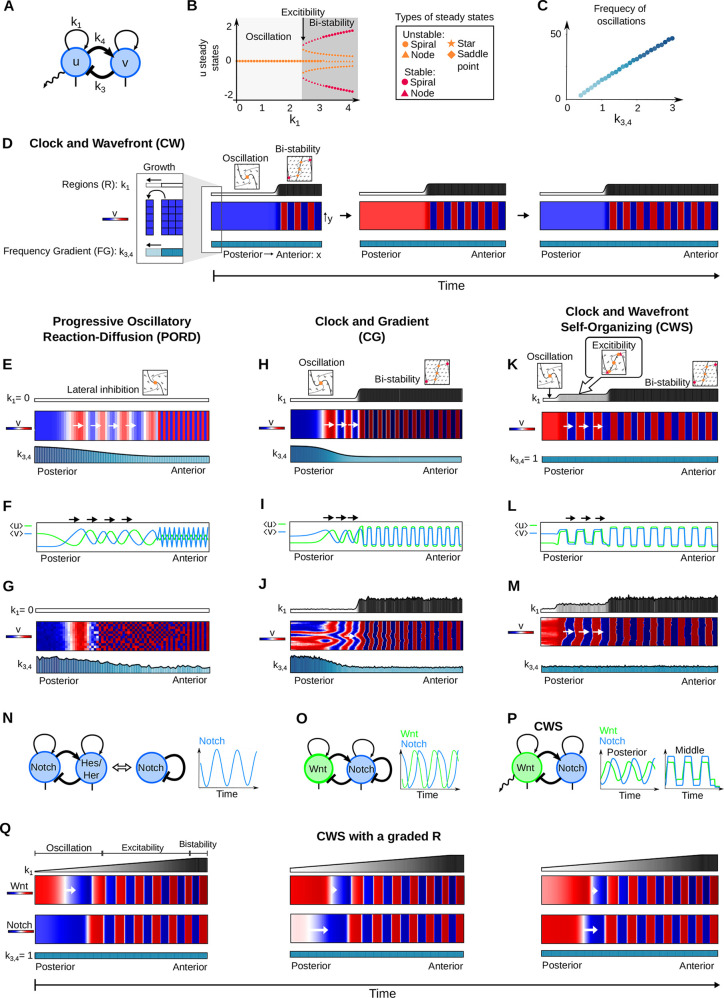
**Previous somitogenesis models and Clock and Wavefront Self-Organizing model in the Sevilletor framework.** (A) Network diagram of the Sevilletor equations. (B) Varying *k*_1_ transitions from oscillatory to bistable states, shown by the bifurcation diagram. (C) Oscillation frequency increases linearly with negative feedback loop strength (*k*_3_ and *k*_4_) for *k*_1_<2.3. (D) Simulation of the Clock and Wavefront model (CW). As the tail grows, adding a new line of cells at the posterior tip, cells exit an oscillatory regime and enter a bistable determination front. (E,H,K) Two-dimensional simulations of Sevilletor implementations of Progressive Oscillatory Reaction-Diffusion (PORD), Clock and Gradient (CG) and Clock and Wavefront Self-Organizing (CWS) models shown for ***v***, with white arrows indicating phase waves. Corresponding Figure for ***u*** is shown in Fig. S15 (Supplementary Section S10). (F,I,L) Average ***u*** (green) and ***v*** (blue) values along the anterior-posterior axis of the simulations above. (G,J,M) Simulated ***v*** patterns with added multiplicative noise to concentrations and parameters. (E-G) The PORD model generates somites sequentially via a relay mechanism triggered by a pre-patterned anterior somite (Movie 6). A frequency gradient (white-blue gradient of *k*_3_ and *k*_4_) directs phase wave formation, which is fragile to noise. (H-J) The Sevilletor implementation of the CG model promotes phase wave formation with a frequency profile and freezes oscillations with a determination front (Movie 7). Noise in the frequency profile disrupts the pattern over time. (K-M) The CWS model adds an intermediate excitability regime to the CW model, where phase waves form without a frequency gradient (Movie 8). (N) Negative feedback between ***u*** and ***v*** can be interpreted as a delayed inhibition of Notch. (O) Delayed inhibition of Notch can coexist with feedback between Notch and Wnt, coupling their oscillations. (P) The CWS model has feedback between Wnt and Notch that drives out-of-phase oscillations in the posterior tip and in-phase oscillations in the middle part, as observed in experiments by Aulehla et al. (2003) and Sonnen et al. (2018). (Q) Simulation of the CWS model with a graded modulation of *k*_1_ shows waves becoming thinner while traveling anteriorly (Movie 9). All parameters are shown in Tables S3 and S4 in Supplementary Section S23.

We simulate these equations on a 2D growing rectangular grid of virtual cells elongating at a constant speed along the *x*-axis, representing the anterior-posterior axis of the developing tail (Materials and Methods). Tail growth occurs via proliferation of the posterior-most cells, generating a new line of cells inheriting concentrations of reactants *u* and *v* (Fig. 4). The functions R and FG have sigmoidal spatial profiles along the *x*-axis, defined as 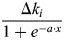, where *x* is the anterior-posterior spatial coordinate, Δ*k*_*i*_ is the amplitude of the sigmoid function, and *a* is its steepness.

The function R regionalizes the anterior-posterior axis into discrete regions with different dynamical regimes using a steep sigmoid function with *a*=1 that modulates parameter *k*_1_ step-wise in space. Theoretical analyses in Figs 2 and 3 reveal *k*_1_ as a key parameter promoting bifurcations and changes in dynamical behavior. This modulation, for example, promotes a bifurcation from a posterior oscillatory regime (*k*_1_<2.3) to an anterior bistable regime (*k*_1_≥2.3), forming the basis to study cell commitment to somite formation as they move anteriorly (Fig. 4A, B). This approach mimics the CW model (Cooke and Zeeman, 1976) when R increases anteriorly with Δ*k*_1_=3 and *a*=1, defining a moving wavefront that promotes cell commitment to a specific phase. Such modulation resembles bifurcations observed in Jutras-Dubé et al. (2020) and François et al. (2007) and in neural tube patterning models (Panovska-Griffiths et al., 2013).

The function FG in Eqns 3 and 4 controls the strength of the negative feedback between *u* and *v* (*k*_3_, *k*_4_), which, similar to the strength of delayed negative feedback in single reactant models, is linearly correlated with oscillation frequency (Fig. 4C).

### A Sevilletor implementation of the PORD model

The first somitogenesis patterning hypothesis we explored is the PORD model from Cotterell et al. (2015), in which somites are formed in a self-organized manner (Supplementary Section S9). Analyzing the original PORD model from Cotterell et al. (2015) through two-cell simulations (similar to Fig. 3B), we discovered that the pattern is formed by a diffusion-driven arrest of oscillations of neighboring cells in opposite phases (Fig. S13). This behavior is equivalent to the lateral inhibition case of the Sevilletor, and to the original case of ‘stationary waves of extreme short wave-length’ introduced by Turing (1952), which differs from the model presented in Wang et al. (2022) where cells are in a default stable state in the absence of diffusion. Thus, to recreate the PORD model within the Sevilletor equations, we assumed all cells in the tail are in an oscillatory regime without regionalization (R=0), with parameters *k*_1_=0 and *D*=1 to trigger lateral inhibition (Fig. S13B).

To form somites, the PORD model requires initial conditions with an anterior pre-patterned somite, triggering a relay mechanism that produces activator peaks spanning few cells as the tail grows. Introducing a frequency gradient that decreases anteriorly with the function FG with Δ*k*_3,4_=0.3 and *a*=0.1 generates phase waves as in the original PORD model (Fig. 4E,F; Movie 6). Subsequent studies reformulated the PORD model, triggering relay mechanisms with a gradient without the need for a pre-patterned somite (Pantoja-Hernández et al., 2021; Kuyyamudi et al., 2022).

Our PORD implementation confirmed the fragility of this model to spatial noise, consistent with studies by Pantoja-Hernández et al. (2021). In both the original PORD model and our implementation, noise disrupts the periodic somite patterns, resulting in a salt-and-pepper pattern (Fig. 4G; Movie 6; Fig. S14). In summary, although the lateral inhibition regime of the PORD model explains anterior oscillation arrest through local cell communication without changing the oscillatory regime, it forms periodic patterns with peaks spanning few cells that are sensitive to noise.

### A Sevilletor implementation of the CG model

The second somitogenesis patterning hypothesis that we explored was the CG model, a popular reincarnation of the CW model in which the frequency of the segmentation clock is modulated to decrease progressively along the anterior-posterior axis, promoting phase wave formation.

In our Sevilletor version of the CG model, both the initiation and arrest of oscillations are emergent features of the dynamical system (Fig. 4H,I; Movie 7). Nevertheless, the oscillatory regime is equivalent to a series of coupled type IIIo oscillators (Cross and Hohenberg, 1993) as in previous CG models (Fig. S9; Supplementary Section S6) (Morelli et al., 2009; Herrgen et al., 2010). The spatial frequency profile is introduced by decreasing the feedback strength between *u* and *v* with the anteriorly decreasing frequency gradient FG with Δ*k*_3,4_=2 and *a*=0.1_,_ similar to the PORD model. In agreement with previous CG models, this frequency profile generates phase waves that become thinner moving anteriorly, and freeze into periodic patterns entering the bistable determination front. This is obtained with a bifurcation from oscillation to bistability promoted by R (Δ*k*_1_=3, *a*=1), similar to previous body segmentation models (François et al., 2007; Jutras-Dubé et al., 2020).

As discussed in previous studies (Oates et al., 2012), variation of the slope or position of the frequency gradient strongly influences the phase wave patterns. The dependence of the model on the frequency profile is also highlighted by the lack of phase waves in the absence of a frequency gradient (Fig. S21J), and by the progressive disorganization of the somite pattern when noise is applied to FG (Fig. 4J). The model is, however, robust to cell movements (Fig. S17).

### The clock and wavefront self-organizing model

Motivated by the observation that in both the PORD and CG models, phase waves arise owing to a frequency profile, we investigated whether the CW model could be extended to form phase waves via local cell-to-cell communication. Our aim was to devise a model where PSM cells can form phase waves independently of frequency gradients and can recapitulate the excitability observed *in vitro* (Hubaud et al., 2017).

The foundational concept of this new model aligns with the basis of our implementation of the CW (Fig. 4D), where cells oscillate posteriorly, transitioning to bistability through a determination wavefront. In our new CWS model (Fig. 4K), we introduced a third discrete middle region in which cells have intermediate values of *k*_1_. This is implemented with the function R that promotes a two step change in *k*_1_ with a posterior and an anterior sigmoid starting from *k*_1_=2.3:
(5)

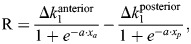
where *x*_p_ and *x*_a_ are the *x* coordinates from the posterior and anterior boundary respectively, Δ*k*_1,anterior_=1.7 to promote bistability, Δ*k*_1,posterior_=1.3 to promote oscillations and *a*=1 to promote steep sigmoids. In the intermediate region, cells are in the default self-organizing excitable diffusion-driven behavior presented in Fig. 3D and Movie 3, giving the name to the model: the Clock and Wavefront Self-Organizing model (Fig. 4K; Movie 8).

The CWS model forms phase waves in the absence of a frequency gradient: FG=0 (white arrows, Fig. 4K). This is possible because of the excitable behavior in the intermediate region for *k*_1_=2.3 that gives rise to coordinated somitogenesis waves moving anteriorly within the tail. The excitable behavior would generate periodic phase waves with spirals starting from random initial conditions (Fig. 2E), but within the tail the same behavior robustly propagates phase waves because of the proximity to the oscillatory region in the posterior end that initiates the waves (Figs S6,S7). Thus, the patterning behavior in the intermediate part of the CWS model can be described as a guided self-organizing process in which posterior oscillations guide the phase waves that move anteriorly (Morales et al., 2021). This is illustrated in simulations where the tail of the CWS model is cut in two, separating the posterior from the middle part of the tail. In agreement with previous experiments (Özelçi et al., 2022), the simulations show that the posterior oscillations guide the alternation of phases in the border to the excitable middle region, whereas the propagation of waves is a self-organizing process (Fig. S20; Supplementary Section S13; Movie 10).

When the posterior oscillatory region is expanded, the model predicts the formation of multiple phase waves in the absence of a frequency gradient, providing a possible explanation for the multiple phase waves observed upon expansion and saturation of posterior gradients in mouse mutants (Aulehla et al., 2008) (Fig. S21; Supplementary Section S14). In addition, our analysis revealed that the CWS model exhibits a high degree of patterning robustness when exposed to multiplicative noise in comparable amounts with the PORD and CG models (Materials and Methods; Fig. 4M; Movie 8). We have further tested the CWS model to show that it is also robust to cell movements (Fig. S17), hexagonal lattice (Fig. S26; Supplementary Section S19), temporal fluctuations in noise (Fig. S16; Supplementary Section S11), changes in the length of the oscillatory posterior tailbud (Fig. S19; Supplementary Section S12) and tail width (Fig. S18). The model can also recapitulate the change in somite length observed with slower oscillations and tail growth (Fig. S25; Supplementary Section S18; Herrgen et al., 2010; Goudevenou et al., 2011).

Finally, our analysis revealed that the excitable behavior in the intermediate part of the tail is possible for a broad set of intermediate *k*_1_ values and diffusion constant *D* (Figs S10,S12), provided a sufficiently small spatial discretization dx (Fig. S11).

### Possible molecular implementations of the CWS model for mouse somitogenesis

The general aim of this study was to explore how global and local synchronization of oscillations drive somitogenesis in different models, without capturing the underlying molecular details. The Sevilletor equations can, however, also provide insights into the minimal regulatory terms between two reactants that give rise to specific patterning behaviors. In this section, we discuss two alternative molecular interpretations for the CWS model and relate each interpretation to experimental observations. We focus our analysis on the negative feedback between *u* and *v*, which is the core regulatory topology of the Sevilletor equations that implements a delayed negative feedback without explicitly representing delays in the equations (Casani-Galdon and Garcia-Ojalvo, 2022).

The first molecular interpretation is that the negative feedback represents a transcriptional inhibition of Notch mediated by Hes/Her proteins, a crucial motif in the vertebrate segmentation clock (Takashima et al., 2011; Ay et al., 2013). In this interpretation, *u* corresponds to Notch and *v* to a Notch signaling effector such Hes7 (Fig. 4N), with diffusion representing juxtacrine Notch signaling (Ferjentsik et al., 2009; Jiang et al., 2000). Our analysis suggests a diffusion constant for Notch of ∼0.05 μm^2^/s to generate somites every 2-3 h, consistent with juxtacrine signaling (Sonnen et al., 2018; Matsuda et al., 2020).

The second molecular interpretation is that the negative feedback represents coupling between Notch and Wnt signaling pathways, in accordance with their coordinated oscillations during mouse somitogenesis (Aulehla et al., 2003; Sonnen et al., 2018). Perturbation experiments support this hypothesis, showing entrainment between Wnt and Notch oscillations (Sonnen et al., 2018). In this scenario, *u* represents Wnt and *v* Notch. An extended model presented in Supplementary Section S5 (Fig. S8) demonstrates that the Wnt-Notch feedback can coexist with a delayed negative feedback of Notch to drive sustained oscillations (Fig. 4O). For simplicity, in the rest of the study, we considered a reduced model with only the negative feedback between Wnt and Notch, which is sufficient to drive oscillation and to couple the two signaling pathways (Fig. 4P).

This molecular interpretation predicts that Wnt and Notch oscillate out of phase posteriorly and in phase in the middle part of the tail, consistent with previous findings (Aulehla et al., 2003; Sonnen et al., 2018) (Fig. 4L). This relative phase change arises as the cells move from the oscillatory posterior part with oscillations with a phase shift around an unstable state, to the excitable region where the cells have a long permanence time around the stable states where Wnt and Notch are in phase (Fig. S24; Supplementary Section S17). This is possible only when Notch has a negative influence on Wnt in agreement with Acar et al. (2021), and when Wnt has a positive influence on Notch, as previously proposed in Aulehla et al. (2003) and Gibb et al. (2009), see Supplementary Section S16 (Fig. S23). This phase shift is also observed in an extended CWS model with graded *k*_1_ modulation capturing wave thinning and the faster propagation of Notch waves with respect to Wnt waves (Sonnen et al., 2018) (Fig. 4Q; Movie 9).

Finally, in agreement with previous measurements (Hatakeyama et al., 2023; Kicheva et al., 2007), this implementation predicts that Wnt diffusion must be in the order of 0.05 μm^2^/s to generate somites every 2-3 h (see Supplementary Section S22). Importantly, adding diffusion of Notch or higher levels of Wnt diffusion does not alter the qualitative behavior of the model (Fig. S22; Supplementary Section S15).

### The excitability of the CWS model recapitulates the behavior of the mouse PSM *in vitro*

**Fig. 5.**
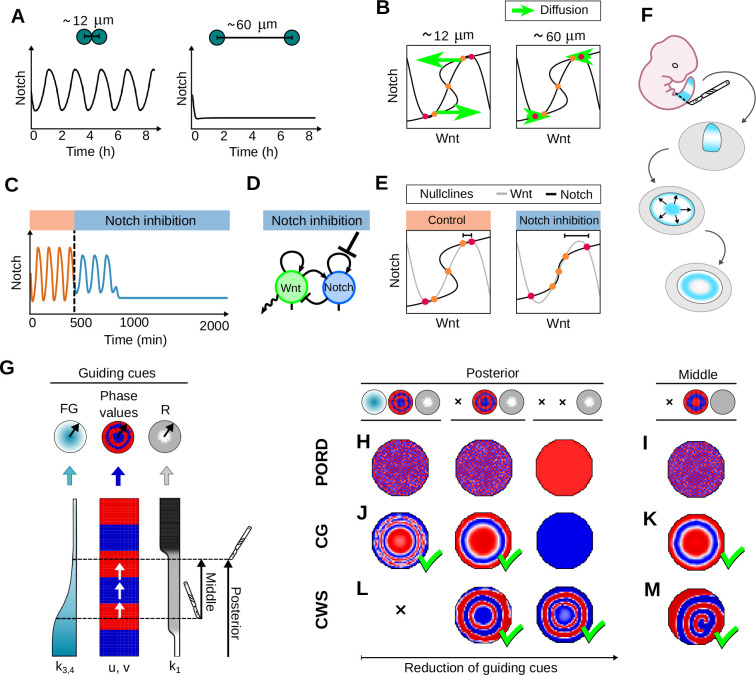
**The CWS model recapitulates the behavior of the mouse PSM *in vitro*.** (A) Simulations of two cells in the CWS excitable regime that are excited to oscillate at high density (12 μm distance) and stop oscillating in low density (60 μm distance), as observed in Hubaud et al. (2017) (model units estimated in Supplementary Section S22). (B) Phase spaces show that when cells are in high density they excite each other to oscillate, leaving stable states (red dots), towards the trajectory of the unstable states (orange dots) due to the stronger contribution of diffusion (green arrows). (C) Simulation of two cells in the CWS excitable regime where oscillations are dampened and stopped by inhibiting Notch, as seen in Hubaud et al. (2017). (D) Illustration of Notch inhibition in the Sevilletor network (*k*_2_=0). (E) Phase spaces showing altered nullcline of Notch with inhibition, increasing the excitability threshold (black marker). (F) Illustration of circular signaling waves formation in mouse presomitic mesoderm (PSM) explants. (G) Illustration of how virtual explant simulations are created, with a radial projection and mixing of cells from the simulated tails. (H,J,L) Explants from the whole posterior part of the Sevilletor implementations of the PORD, CG and CWS models under different guiding cues shown for ***v*** (illustrated in the top row). Corresponding Figure for ***u*** shown in Fig. S27. (I,K,M) Explants from the middle part of the tail with phase values inherited from the tail. (H,I) PORD explants form chessboard patterns with the FG gradient and/or phase values, and homogeneous oscillations form from the same initial phase (without phase values pre-pattern in the third explant in H). (J,K) CG explants form circular waves with a gradient FG and/or phase values, and homogeneous oscillations from the same initial phase (third explant in J). (L,M) CWS explants do not possess frequency gradients and form circular waves, even starting from the same initial phase (Movies 11 and 12). All parameters of the simulations are shown in Table S5 (A-E) and Tables S6 and S7 (H-M) in Supplementary Section S23.

A previous study has shown that mouse PSM cells stop Notch target oscillations like Lfng at low density *in vitro* cultures on fibronectin, but can be excited to oscillate when the density is increased (Hubaud et al., 2017). The excitable behavior of the CWS model offers a new mechanistic explanation for this phenomenon. Indeed, we found that increasing the distance between two cells beyond 60 μm in the model, led to the spontaneous arrest of oscillations (Fig. 5A). The model displays identical bistable phase portraits in the low and high cell density situation (see red and orange points in Fig. 5B), but as the distance between cells increases, the weaker diffusion effect fails to push Wnt and Notch out of bistability toward the trajectory of the nearest unstable points, explaining the arrest of oscillation (see green arrows in Fig. 5B). This diffusion-driven excitable behavior differs from the one of the FitzHugh Nagumo equations used in Hubaud et al. (2017) (see Supplementary Section S3) and recapitulates the effect of cell density without changing other parameters in the model.

In addition, our analysis revealed that excitability in the CWS model depends not only on the distance between cells, but also on the distance between stable and unstable steady states in phase space (Materials and Methods). Previous experiments have proposed that inhibiting Notch signaling reduces the stimulus that triggers excitability, leading to the arrest of oscillations observed in the LuVeLu reporter (Hubaud et al., 2017) (Fig. 5C). In the CWS model, the self-enhancement of Notch can be interpreted as positive feedback in response to autocrine Notch signaling (Bone et al., 2014). Notably, inhibiting Notch self-enhancement in the model results in oscillation arrest even at high density (Fig. 5C-E), by increasing the distance between stable and unstable steady states, thereby elevating the excitability threshold of cells (see red and orange points in Fig. 5E).

In a similar vein, our model suggests that the oscillations observed in low-density cultures on fibronectin upon Yap signaling inhibition (Hubaud et al., 2017) may be attributed to a decrease in the distance between stable and unstable steady states, lowering the excitability threshold. Although the mechanism by which Yap signaling normally increases this threshold remains unclear, one possibility is that Yap inhibits Notch signaling. However, upregulation of Notch signaling via Dll1 addition in low-density cultures is insufficient to rescue oscillations (Hubaud et al., 2017). An alternative prediction of our model is that Yap may promote Wnt auto-activation, associated with higher values of *k*_1_ that increase the excitability threshold, shown in Fig. 2F for *k*_1_≥2.3. This hypothesis is consistent with the observation that fibronectin increases from posterior to anterior in the chick PSM (Duband et al., 1987) increasing Yap (Hubaud et al., 2017), which in turn could increase the rate of Wnt auto-activation (*k*_1_) as shown in Fig. 4Q, owing to cross-talk between the pathways (Jiang et al., 2020). A direct way to test this hypothesis would be to check whether the inhibition of canonical Wnt signaling can rescue the oscillation of PSM cells in low density culture grown on fibronectin.

Finally, another prediction of our model is that the diffusion-driven excitable behavior arises between cells that lie in opposite half-planes in phase space (see Movie 3 and Fig. 3D). This promotes oscillations that originate from the destabilization of opposite stable states leading to out-of-phase oscillations in neighboring cells. Intriguingly, a quantification of the oscillations observed in the low-density culture shown in Hubaud et al. (2017) revealed that neighboring cells oscillate out of phase in agreement with the prediction of the model (Fig. S32; Supplementary Section S21).

Next, we explored how local cell communications contribute to generating coherent somitogenesis wave patterns in virtual explants of the PORD, CG and CWS models.

### Explants of the PORD, CG and CWS models

In our tail simulations of the Sevilletor implementations of the PORD, CG and CWS models shown in Fig. 4E, H, K, consistent periodic somitogenesis patterns emerge because of synchronization of oscillations in the PSM but also owing to the growth dynamics and geometry of the tail, which act as global patterning cues. Tail explants enable examination of PSM patterning behavior when these guides are perturbed. Previous studies have shown that explants can generate sequential waves of Notch signaling that propagate from the center of the explant, resembling somitogenesis (Lauschke et al., 2013; Hubaud et al., 2017; Tsiairis and Aulehla, 2016). Depending on the protocol used, the waves ceased after a few cycles, together with re-establishment of global signaling gradients (Lauschke et al., 2013), or persisted over 2 days in the presence of activators of Fgf and inhibitors of RA, among others, without detected graded signals of Fgf targets (phosphorylated ERK and Spry2) (Hubaud et al., 2017) (detailed description in Supplementary Section S20).

As our study focuses on understanding the mechanisms that drive the spatial synchronization of oscillations, we conducted virtual explant simulations to investigate initial formation of traveling waves observed in Lauschke et al. (2013) and Hubaud et al. (2017). A virtual explant is created by projecting a part of the tail into a circular domain, with the most posterior end at the center and the most anterior part at the outer edge (Fig. 5G) (more details provided in Supplementary Section S20: Detailed description of explant simulations and experiments). Given uncertainty about whether these waves depend on global signals or local communication, we simulated explants with varying levels of guiding cues that can be provided by re-established frequency gradients FG (*k*_3_ and *k*_4_) and the phase values that the cells had in the tail at the time of dissection (*u* and *v*) (Fig. 5H-M).

Our simulations showed that, when cells inherit phase values, our PORD explants tended to form chessboard patterns even in the presence of frequency gradients (Fig. 5H; Fig. S30B), while they gave rise to homogeneous oscillation starting from the same initial phase. This is due to the model's intrinsic tendency to generate lateral inhibition patterns (Supplementary Section S9). On the other hand we observed that when CG explants inherit phase values, they are able to self-organize to form circular wave patterns even in the absence of frequency gradients. Yet, they form homogeneous oscillations starting from the same initial phase (Fig. 5J; Fig. S30C). Finally, we found that CWS explants can form circular wave patterns even when cells have the same initial phase value and in the absence of frequency gradients (Fig. 5L; Movie 11; Fig. S30D). Ablating the central population in this explant, as in previous experiments (Hubaud et al., 2017), still generates periodic waves that progressively disorganize or dissipate depending on initial phase values (Fig. S29). Moreover, circular wave patterns can also be observed in CWS explants derived solely from the middle part of the tail, where all cells are in the excitable regime (Fig. 5M; Fig. S31) independent of a central cell population (Movie 12).

To further test the self-organizing capacity of virtual explants in the CG and CWS models, we randomized cell positions to disrupt any pre-pattern inherited from the tail. Similar experiments (Tsiairis and Aulehla, 2016; Hubaud et al., 2017) have been performed by centrifuging cells from tails of different mouse embryos. Our simulations revealed that, in both the CG and CWS models, mixed explants form rotating wave patterns (Fig. S28; Movie 13) that arise from the local synchronization between cells with different phase values (Uriu et al., 2021).

It remains unclear how these coordinated rotating patterns emerge in mixed explants. Future experiments could explore these self-organizing behaviors further by mixing the intermediate part of multiple tails to maximize the initial heterogeneity of phase values in the explants. Evaluation and removal of potential re-established gradients such as Fgf should also be performed and a non-adherent culture should be used to eliminate potential guiding cues.

## DISCUSSION

During embryonic development, cells need to coordinate their behaviors to form coherent spatial patterns that drive tissue specification. One way to achieve this coordination is by responding to global signals, such as morphogen gradients that provide positional information to the cells (Wolpert, 1969). Alternatively, self-organizing spatial patterns can be formed by coupling cell-autonomous behaviors at the tissue level through local cell communication (Turing, 1952). In addition, increasing evidence is showing that these two patterning strategies are not exclusive and that embryonic development is often controlled by self-organizing processes guided by external global signals (Morales et al., 2021).

The sequential waves of gene expression observed during vertebrate somitogenesis are a striking example of this coordination that arise from the spatial synchronizations of genetic oscillations. In this study, we introduced a system of equations named Sevilletor to investigate how oscillations can be synchronized by global spatial modulations or local cell-to-cell communication. We showed that this minimal phenomenological framework can be used to compare the qualitative behaviors of different somitogenesis models, such as the CW model (Cooke and Zeeman, 1976), the PORD model (Cotterell et al., 2015) and the CG model (Morelli et al., 2009; Herrgen et al., 2010; Jörg et al., 2016).

Using this phenomenological framework, we remain neutral regarding the source of global spatial modulation that controls oscillation arrest, which could arise from morphogen gradients or cell-autonomous regulations (Rohde et al., 2021 preprint; Boareto et al., 2021). Instead, we explore whether phase wave formation and the excitability of mouse PSM observed *in vitro* can be driven by local cell communication.

As, in our basic implementation of the CW model, posterior cells were in an oscillatory regime and anterior cells in a bistable regime, we envisioned an extended CW model where intermediate cells are in a novel diffusion-driven excitable regime (Movie 3). Remarkably, cells in the intermediate region were able to sustain and propagate phase waves independently of global frequency gradients, solely relying on local cell interactions showing a high degree of robustness (Movie 8). We named this model the CWS model to highlight the hypothesis that intermediate cells are in a self-organizing regime.

The key distinction between the PORD, CG and CWS models lies in how phase waves emerge. In the PORD and CG model, phase waves arise within a pure oscillatory state via frequency modulation along the anterior-posterior axis, governed by parameters *k*_3_ and *k*_4_ in the Sevilletor equations (Fig. 4H). In contrast, in the CWS model, phase waves emerge at the interface between an oscillatory state and bistability, modulated by parameter *k*_1_ along the anterior-posterior axis (Fig. 4K). This is promoted by the excitation of PSM cells that are in a bistable state, which differs from the default behavior of PSM cells in zebrafish (Maroto et al., 2005; Masamizu et al., 2006) and aligns with the excitable behavior of mouse PSM cells *in vitro* (Hubaud et al., 2017).

In a previous study, this excitable behavior was investigated using a single-cell model based on the FitzHugh-Nagumo equations. The difference between low- and high-density cultures in this model was simulated by adjusting the magnitude of a stimulus that can excite a single stable steady state (Hubaud et al., 2017). In contrast, in the CWS model the transition from a quiescent to an oscillatory state emerges from a bistable regime. In this case, cells are excited to oscillate by the stronger influence of diffusion at high density, without having to change any other parameters in the model (Fig. 5A; Supplementary Section S3).

We further hypothesized that, alongside the cell-autonomous negative feedback of Notch (Takashima et al., 2011; Ay et al., 2013), the segmentation clock underlying the CWS model might involve a negative feedback loop between Notch and Wnt signaling, consistent with previous observations in mouse (Aulehla et al., 2003; Sonnen et al., 2018). According to this assumption, the model recapitulated that Notch and Wnt oscillate out of phase in the posterior part of the tail, but oscillate in phase in the middle part (Aulehla et al., 2003; Sonnen et al., 2018), coinciding with excitability and phase wave formation. Additionally, our analysis revealed that the excitability of the CWS model is determined by the distance between stable and unstable states in phase space. This insight helped us interpret the arrest of oscillations upon Notch signaling inhibition in high-density cultures and the induction of oscillations upon Yap inhibition in low-density cultures as changes in excitability (Hubaud et al., 2017). Moreover, it suggested that Yap may increase the excitability threshold of the mouse PSM by modulating Wnt signaling via the known cross-talk between the two pathways (Jiang et al., 2020).

Finally, we conducted virtual explant simulations to further assess the self-organizing capabilities of the CG and CWS models. These simulations aimed to examine the behavior of mouse PSM cells outside the embryonic context, which can generate coherent circular wave patterns despite significant cell re-arrangements (Lauschke et al., 2013; Hubaud et al., 2017). We found that both the CG and the CWS models can form coherent circular phase waves in the absence of frequency gradients if the cells inherit the phase values possessed in the tail. On the other hand, we found that the CWS model could also form coherent wave patterns starting from homogeneous phase values.

In a broader context, the Sevilletor model offers a minimal theoretical framework for exploring multicellular pattern formation via synchronized oscillations. Our study focuses on phase wave formation and excitability, specifically examining the interplay between Notch and Wnt signaling pathways during mouse somitogenesis. However, the Sevilletor equations can generate various patterns with minimal parameter changes, including lateral inhibition chessboard patterns like those mediated by the Notch signaling in retina (Formosa-Jordan et al., 2013) and inner ear development (Adam et al., 1998; Petrovic et al., 2014). As has been previously suggested, modulators and co-factors may influence the timing or strength of the Notch pathway, promoting different patterning outcomes such as lateral inhibition or phase wave synchronization (Liao and Oates, 2017). Looking ahead, we anticipate that our framework could be used to explore how changes in signaling pathway feedback drive different self-organizing behaviors.

## MATERIALS AND METHODS

### Theoretical analysis of the Sevilletor system

We devised the Sevilletor equations as a minimal reaction-diffusion system to study how genetic oscillations can synchronize and self-organize in space through local cell-to-cell communication (Fig. 2A,B). The following section is dedicated to present theoretical properties of the network, and assumes that the reader is familiar with some aspects of complex systems theory, such as the notion of steady states, stability analysis, bifurcation diagrams and phase portraits (Murray, 2002; Sneppen, 2014).

The system consists of two partial differential equations representing interactions between two reactants named *u* and *v* (Eqns 1 and 2). The dynamics of the system are centered around the fixed point (u*, v*)=(0, 0), which represents intermediate concentrations. However, the equations can be easily adjusted to form only positive values without affecting the behavior of the system (see Supplementary Section S1 and Fig. S1).

The core of the system is a negative feedback between the two reactants *u* and *v*, controlled by the rates *k*_3_ and *k*_4_, which gives rise to a limit cycle centered on (u*, v*) that promotes oscillations. This is the simplest regulatory logic that can generate oscillations without explicitly adding delays to equations (Casani-Galdon and Garcia-Ojalvo, 2022). The sustained oscillations generated by this model are equivalent to the one generated by a single reactant model with a delayed negative feedback through a sufficiently large monotonically decreasing function (Lewis, 2003).

In addition, the model considers cubic saturation terms that limit the deviation of concentration from the fixed point (u*, v*). These negative saturation terms should not be interpreted as degradation terms, but rather as an effective symmetric saturation for concentrations that are far from the fixed point but without significant effects for concentrations closer to the fixed point.

Finally, the model includes two positive self-regulatory feedbacks for each reactant, controlled by *k*_1_ and *k*_2_, which, together with saturations, determine the number of fixed points of the system. Overall, the Sevilletor equations can be viewed as an extension of the first-order formulation of the Van Der Pol oscillator (FitzHugh, 1961) (Supplementary Section S3) with an additional linear self-enhancing feedback and cubic saturation term in Eqn 2.

To characterize the behavior of the system, we focused our analysis on the effect of the positive feedbacks *k*_1_ and *k*_2_, as these are the two key parameters that drive bifurcations. We generally consider the non-dimensionalized version of the model for *k*_2_ by rewriting the system as *k*_2_→1, *k*_1_/*k*_2_→*k*_1_, *k*_3_/*k*_2_→*k*_3_ and *k*_4_/*k*_2_→*k*_4_. Importantly, for each set of reaction parameters, we also investigated the behavior of the system in the presence of diffusion by allowing the reactant *u* to diffuse with diffusion constant *D*=0.3. For simplicity, we considered the reactant *v* to be immobile, as spatial coupling with the diffusion of one reactant (*u*) is enough to synchronize oscillations in space and to promote self-organizing patterning behaviors (Prigogine and Lefever, 1968; Nicolis and Prigogine, 1977; Field and Noyes, 1974; Cotterell et al., 2015). In our full somitogenesis model Eqns 3 and 4, however, we also explored the case where *v* diffuses and find equivalent theoretical predictions (Fig. S22).

#### Self-enhancement strength and initial conditions determine Sevilletor patterning dynamics

The bifurcation diagram in Fig. 2C shows how *k*_1_ affects the number of steady states and their stability. For all values of *k*_1_, there is an unstable steady state at (u*, v*)=(0, 0), and for *k*_1_≥2.3 the system undergoes a bifurcation that adds four additional steady states (Fig. 2F). The two steady states furthest away from the center are stable, whereas the other three are unstable. This bifurcation is illustrated by 2D numerical simulations started with homogeneous initial conditions (Fig. 2D,E, left column), showing homogeneous synchronized oscillations for 0≤*k*_1_<2.3 and homogeneous static patterns for *k*_1_≥2.3 associated with bistability.

Starting from random initial conditions, however, numerical simulations show a variety of complex oscillatory and static patterns (Fig. 2E, right column) depending on the parameter *k*_1_ (Movie 1). These include lateral inhibition patterns for *k*_1_=0, which are characterized by cells with alternating opposite concentrations, rotating waves when *k*_1_=1, periodic wave patterns for *k*_1_=2.3, propagating bistable fronts that generate homogeneous static patterns when *k*_1_=3 and bistable frozen states for *k*_1_=4 (Movie 1). These complex patterns arise because of the combination of reaction and diffusion in the system. As classic phase portraits only take into account the contribution of reactions, we extended our complex system analysis to study how phase portraits change with diffusion between two cells.

#### The effect of cell-to-cell communication on patterning behaviors

We use a simplified version of the Sevilletor with only two cells to study how diffusion affects the patterning behavior. The cells start with a heterogeneous initial state with (*u*_1_, *v*_1_)=(0.1, 0) and (*u*_2_, *v*_2_)=(−0.1, 0). We run two simulations for each representative value of *k*_1_: one without diffusion (*D*=0), and one with diffusion (*D*=0.3) (Fig. 3B-F). Without diffusion, each cell acts as an individual unit and its behavior depends solely on changes driven by reaction (Fig. 3B-F, left columns). This case corresponds to the behavior seen in square 2D simulations with a homogeneous initial state (Fig. 2E, left column), as when every cell has the exact same amounts of *u* and *v* there is no active contribution from diffusion, i.e. 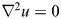. By including cell communication in the form of diffusion of *u* (Fig. 3B-F, right columns), the combined effect of reaction and diffusion coordinates the behavior of the two cells, giving rise to large scale patterns.

With *k*_1_=0 and *k*_1_=1, the cells oscillate individually in the limit cycle without diffusion. For *k*_1_=0, with diffusion these oscillatory trajectories are counterbalanced and stabilized by the effect of diffusion (Fig. 3B; Movie 2) (Singh and Sinha, 2013). Our 2D simulations show that this behavior generates a lateral inhibition (chessboard) pattern from noise (Fig. 2E; first column in Movie 1). With *k*_1_=1, with diffusion the cells continue to oscillate by following a smaller limit cycle that, in the long run, synchronizes the two cells together as in a type IIIo system (Cross and Hohenberg, 1993) (Fig. 3C). Using random initial concentrations that lay on one of the half planes, this behavior is associated with homogeneous oscillations (Fig. S7; Supplementary Section S4). However, starting from random initial concentrations spread on the two half planes, the system generates rotating spirals similar to those formed by a diffusive Van der Pol oscillator (Fig. 2E, right column; Fig. S5C; second column in Movie 1).

At the bifurcation point *k*_1_=2.3, in the absence of diffusion, the two cells do not oscillate and are trapped at the nearest stable state on the upper or lower half plane (Fig. 3D). When diffusion is added, however, the equilibrating effect of diffusion pushes cells out of stability towards the trajectory of the closest stable point (green arrows in Fig. 3D). Following this trajectory, each cell goes to the opposite half-plane towards the stable steady state, and it is again destabilized by diffusion. The repetition of this process generates a novel limit cycle that keeps cells oscillating (Movie 3). This new limit cycle generates in-phase oscillations of *u* and *v*, because the permanence time around the stable states is greater than the time it takes to follow the trajectory to the opposite half-plane. In 2D simulations with random initial conditions, these dynamics give rise to a new type of diffusion-driven excitable periodic wave pattern with spiral formation that, to the best of our knowledge, has not been described previously (Fig. 2E; third column in Movie 1) (see detailed analysis in Supplementary Section S3). The pattern looks very similar to those formed by classic models of the Belousov-Zhabotinsky reaction (Zhabotinsky and Zaikin, 1973) such as the Brusselator (Prigogine and Lefever, 1968) and Oregonator (Field and Noyes, 1974); however, it emerges from a different dynamical behavior that has not been described in previous models (Supplementary Section S3). The limit cycle that underlies periodic wave patterns is possible for a variety of values of *k*_1_ and diffusion constant *D* (Fig. S10; Supplementary Section S7) and spacial discretisation (Fig. S11; Supplementary Section S8).

This dynamical behavior changes for larger values of *k*_1_. For example, for a self-enhancement strength with *k*_1_=3, a few oscillations are stimulated, but the two cells eventually freeze together at the same stable state (Fig. 3E). This makes a propagating front that covers the whole domain in square 2D simulations (Fig. 2E; fourth column of Movie 1). For even stronger self-enhancement with *k*_1_=4, the distance between stable states and saddle points is too large for diffusion to impact the trajectory of the cells (Fig. 3F), making a static bistable frozen pattern that amplifies the pre-pattern present in the initial conditions (Fig. 2E; fifth column of Movie 1).

A characteristic of the Sevilletor system is that its patterning dynamics can be changed by varying just the parameter *k*_1_. This property can also be exploited to easily switch between different patterning behaviors over time (Movie 4).

#### The relative self-enhancement strength and diffusion determines the dynamical behavior of the system

To further characterize the effect of both self-enhancements, we performed a 2D simulation of the full system with Eqns 1 and 2 by increasing the values of *k*_1_ and *k*_2_ along the *y*- and *x*-axis to recapitulate all the different dynamical behaviors of the system within the same simulation (Fig. 3H; Movie 5). The corresponding 2D bifurcation diagram (Fig. 3I) shows the bifurcation between 1, 5 and 9 steady states. This bifurcation diagram was further divided into regions of different patterning behaviors by calculating the number of oscillations of a series of two-cell simulations with diffusion (as in Fig. 3B-F right columns) for each combination of parameters (*k*_1_, *k*_2_) in a 100×100 grid for a total of 10,000 two-cell simulations (Fig. 3I). This allowed us to derive an extended bifurcation diagram in which different colored regions correspond to the different dynamical behaviors that the system can generate with diffusion (Fig. 3J). This extended bifurcation diagram shows that, starting from random initial conditions, the relative self-enhancement strength of *k*_1_ and *k*_2_ together with diffusion determine the different patterning behaviors of the system.

### Quantification and statistical analysis

#### Stability and types of steady states

The steady states of the Sevilletor model and their properties are calculated using a phase plane analysis (Murray, 2002). The steady states of a system are all pairs (u*, v*) for which 
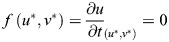
 and 
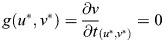
. The stability of the steady states are found by using linear stability analysis from the determinant and trace of the matrix *A*:
(6)

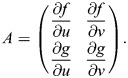
For Det(*A*_(u*,v*)_)>0 and Trace(*A*_(u*,v*)_)<0 the steady state is stable, otherwise it is unstable. The type of steady state is identified as discussed in Appendix A in Murray (2002) and describes the shape of the vector field around the steady state.

#### Calculation of the phase and the number of oscillations of two cell simulations

**Fig. 6.**
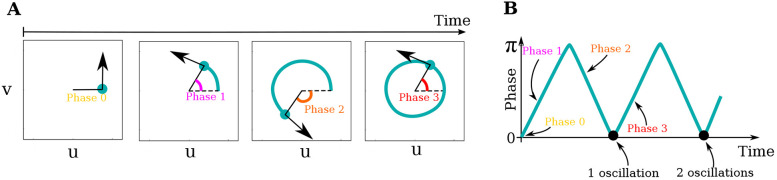
**Details of the calculation of phase and number of oscillations in** Fig. 3. (A) The timeline shows the path of a cell in the phase space. The phase is calculated for each time point, starting from 0, increasing to π for half a loop, and decreasing back to 0 for the second half of the loop. (B) The phase is plotted as a function of time and the number of oscillations is measured as the number of local minima in phase=0 excluding the initial minimum for *t*=0, marked by black points.

The phase in Fig. 3 is calculated, at every time point, as the angle between the initial position and the position of the cell in phase space around the central unstable steady state in (*u*, *v*)=(0, 0).
(7)

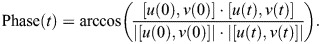
The number of oscillations Fig. 3 is determined by counting the local minima that are close to zero along the phase profile, excluding the first minimum for *t*=0 (see Fig. 6 for calculation).

### Simulation details

Simulations are run with a finite difference solver written in Julia 1.6.4. The complex systems analysis and two-cell simulations have been run in Mathematica 12, and Fiji (ImageJ) has been used to analyze the patterns seen in the simulated and experimental explants. Python 3.8.10 is used to create all plots, excluding the phase spaces and bifurcation diagrams, which have been generated using Mathematica 12.

#### Time discretization

In all simulations, we discretize time using a Euler method to update the values of *u* and *v* for each cell in the system with position (*x*, *y*):
(8)

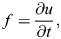

(9)

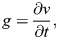

(10)

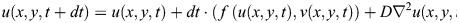

(11)

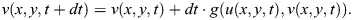


The values used for *D* and *dt* are given in Tables S1-S7 in Supplementary Section S23.

#### Space discretization

The diffusion term 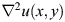 in the system is calculated using a first order finite difference scheme to calculate the discrete Laplace. This is done by using a Taylor expansion of the functions *u*(*x*, *y*, *t*) and *v*(*x*, *y*, *t*) around the steady state (0,0). *ε*  = dx=dy in the following. For *u* the calculations are:
(12)

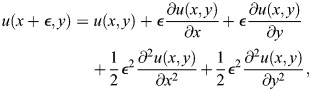

(13)

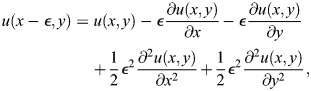

(14)

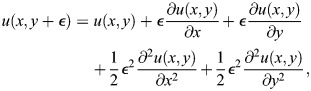

(15)

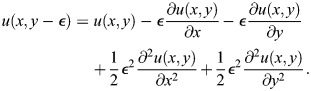


We isolated the first order differential terms in the equations and combined the equations to get:
(16)

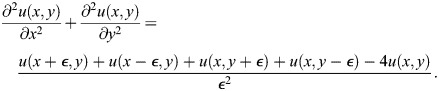
Similar calculations are also performed for *v*. The values used for dx are given in Supplementary Section S23, Tables S1-S7.

#### Boundary conditions

In all simulations, except the one in Fig. S6B, we considered zero flux Neumann boundary conditions. This means that nothing diffuses between the outside and inside of the system, i.e. the Laplace is equal to zero at the boundaries. The Laplace at the boundary is derived as:
(17)

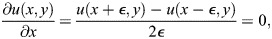

(18)

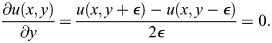


This leads to:
(19)

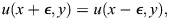

(20)

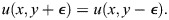


Inserting the appropriate substitutions (Eqns 19 and 20) for the edge/corner into the discrete Laplace Eqn 16 gives the correct formula, for example for the bottom left corner (*x*, *y*)=(1, 1) in Eqn 21:
(21)

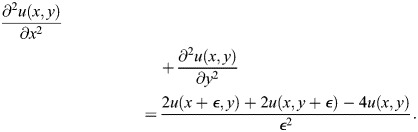
The periodic boundary conditions used for the simulation in Fig. S6B are also calculated using the discrete Laplace, treating cells on the boundaries as direct neighbors with cells on the corresponding boundary. For example, for the cell in the bottom left corner (*x*, *y*)=(1, 1) in a system where the lengths of the axes are *L*:
(22)

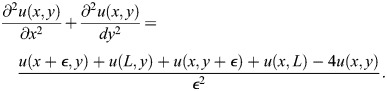


#### Noise

Multiplicate noise is added to the simulations shown in Fig. 4G,J,M and Fig. S16. For each cell *i* the noise is added in the following way at *t*=division:

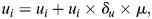


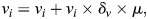
where *μ* is a normally-distributed random number with mean 0 and standard deviation 1. The amplitude *δ* is used to scale the noise to a set percentage of the relevant value (*u*/*v*/*k*_1_/*k*_3_/*k*_4_).

For the parameters *k*_1_, *k*_3_ and *k*_4_, noise is added to the 1D arrays along the *x*-axis at each position *j* at *t*=division with amplitudes *δ*_*k*_:

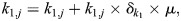


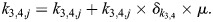


The same array is used for *k*_3_ and *k*_4_. The noise is added to the complete arrays of the parameters, so that as the tail grows, the most anterior parts of the arrays continuously have the same average amount of accumulated noise as the posterior part.

In Fig. 4G,J,M, *δ*  =5% noise is added to the values of *u* and *v* and *δ*  =0.5% noise is added to the parameters *k*_1_, *k*_3_ and *k*_4_ every time a new row of cells is added on the posterior side. An exception is the PORD model, in which no noise is added to the value of *k*_1_=0. In Fig. S16C the amplitude *δ* of the multiplicative noise is sampled from a uniform distribution between 1 and 10% for *u* and *v*, and between 0.1 and 1.0% for *k*_1_, *k*_3_ and *k*_4_.

## Supplementary Material



10.1242/develop.202606_sup1 Supplementary information
