# Supplementary material for: The Clock and Wavefront Self-Organizing model recreates the dynamics of mouse somitogenesis in vivo and in vitro
Source: Development. 2024 May 16;151(10):dev202606. doi: 10.1242/dev.202606 (PMC11165719; doi:10.1242/dev.202606)
Supplement: Supplementary information [file develop-151-202606-s1.pdf]

Supplementary Sections

S1 Sevilletor equations with positive values of u and v 1

S2 Dispersion relation diagrams 2

S3 Oscillations, excitability and bistability in previous models 4
S3.1 Models of the Belousov–Zhabotinsky reaction 5
S3.2 Van der Pol 7
S3.3 FitzHugh-Nagumo model 8

S4 Initial phase values determine the spatial synchronizations 9

S5 The self-regulatory delayed negative feedback of Notch can be coupled with a negative feedback between Wnt and Notch without changing the oscillatory behavior 10

S6 Oscillations of coupled oscillators and with a negative feedback 11

S7 Parameter space that gives rise to periodic wave patterns 12

S8 Spatial discretisation 13

S9 The original PORD model by Cotterell et al. (2015) 15

S10 Wnt patterns in models of virtual tails 18

S11 Fluctuating noise and cell movements 19

S12 Varying tail width and the size of the tip 20

S13 Dissection of the tailbud in the CWS model 21

S14 Extended posterior regions and gradients 22

S15 Variation of diffusion parameters in the CWS model 24

S16 Reversed negative feedback between Wnt and Notch in the CWS 25

S17 Anterior-posterior phase shift of Wnt and Notch oscillations 26

S18 Perturbing period of oscillations and tail growth in the CWS 27

S19 Hexagonal cells 28

S20 Detailed description of explant simulations and experiments 29
S20.1 Wnt patterns in models of virtual explants 29
S20.2 Rotating wave patterns observed in mixed explants 30
S20.3 Ablating the center of explants from the CWS model 31
S20.4 Varying the position of the cut in explants 32
S20.5 Possible outcomes of middle tail explants in CWS model 33

S21 The excitable regime of the CWS model predict out of phase oscillations of neighboring cells 34

S22 Units in the CWS model 35

S23 Parameters (Tables S1-S7) 36

S24 Captions of Movies 41

Development • Supplementary information

Supplementary Figures

S1 Excitable phase waves with positive values of u and v. 1
S2 Dispersion relation diagrams for the steady states of the example systems in Figure 2E 3
S3 Schematic representation of the dynamical regimes that lead to wave formation in the Oregonator model, based on Figure 78 in Cross and Hohenberg (1993) 5
S4 Schematic representation of the dynamical regimes that lead to wave formation in the Sevilletor model 6
S5 Models that produce rotating wave patterns 7
S6 The Sevilletor excitable regime creates straight traveling phase waves in systems with periodic boundaries 8
S7 Spatial synchronizations are determined by initial concentrations of u and v 9
S8 The Sevilletor includes a delayed negative feedback between Wnt and Notch, complementing the direct delayed negative feedback of Notch 10
S9 Oscillations of a single cell for coupled oscillators (Uriu et al., 2021) and for a negative feedback with the Sevilletor equations 11
S10 Parameter values of k_1 and D that give rise to periodic wave patterns 12
S11 Pattern formation with variations of spatial discretisation (size of `dx` = `dy`) and diffusion constant D 13
S12 Somitogenesis models implemented in the Sevilletor framework perform equivalently with re-scaled values of spatial discretisation and diffusion constant D 14
S13 Two cell simulations of the original PORD model by Cotterell et al. (2015) and the Sevilletor implementation 16
S14 Noise in the PORD model creates a disrupted pattern 17
S15 Somitogenesis models in Figure 4 shown for u 18
S16 The CWS model is robust to fluctuating noise 19
S17 Sevilletor implementations of the CG and CWS models are robust to cell movement 19
S18 The CWS model is robust to changes in tail width 20
S19 The CWS model is robust to changes in the length of the tip 20
S20 Virtual dissection of the tailbud in the CWS model 21
S21 Expansion of posterior signals in Aulehla et al. 2008 Nature Cell Biology, and variations in the positions of `R` and `FG` in the CG and CWS models 23
S22 Including diffusion of Notch to the CWS model does not affect the overall dynamics 24
S23 Alternative implementation of the CWS model when the signs of the negative feedback loop between Wnt and Notch are inverted 25
S24 Description of the dynamics of phase change of Wnt and Notch in the Clock and Wavefront Self-Organizing (CWS) model 26
S25 Perturbations affect the size of somites in the CWS in agreement with experiments 27
S26 The posterior part of the tail with the CWS model on a grid with hexagonal cells shown without growth 28
S27 Somitogenesis explant models in Figure 5 shown for u 29
S28 The Sevilletor implementations of the CG and CWS models recapitulate the rotating wave behavior observed in mixed explant experiments shown in Movie S3 presented in Hubaud et al. (2017) 30
S29 Phase waves keep propagating after ablating the center of explants derived from the Clock and Wavefront Self-Organizing model 31
S30 Varying the position of the cut in explants 32
S31 Variability in virtual middle explants of the Clock and Wavefront Self-Organizing model 33
S32 Pairs of cells with out-of-phase oscillations of LuVeLu 34

Development • Supplementary information

S1 Sevilletor equations with positive values of u and v

The patterns generated by the Sevilletor model are centered around the fixed point $(u^*, v^*) = (0, 0)$, which represents intermediate concentrations. In this context, negative values do not represent negative concentrations but rather a decrease from intermediate values. The equations can easily be modified to generate only positive values by substituting u and v in equations (1) and (2) as $u \to (u - c)$ and $v \to (v - c)$, as shown in equations (S1) and (S2). An example is shown in Fig. S1B, where a periodic wave pattern is formed centered around the value $c = 2$ with $k_1 = 2.3$ and $D = 0.3$.

$$\frac{\partial u}{\partial t} = k_1(u - c) - (v - c) - (u - c)^3 + D\nabla^2(u - c), \tag{S1}$$

$$\frac{\partial v}{\partial t} = (v - c) + (u - c) - (v - c)^3. \tag{S2}$$

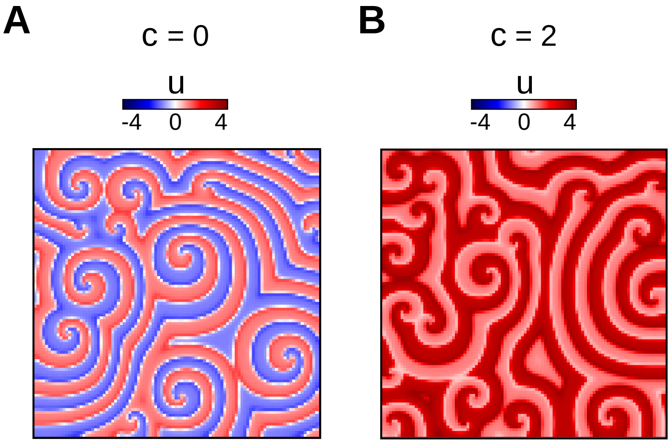

Fig. S1: **Excitable phase waves with positive values of u and v.** A periodic wave pattern with spirals generated by equations (S1) and (S2) forms from initial noise around the steady state $(u, v) = (0, 0)$ with $c = 0$ (A) and around the steady state $(u, v) = (2, 2)$ with $c = 2$ (B). In the latter case all phase values are positive. $k_1 = 2.3$ and $D = 0.3$. All parameters are shown in Tables S1 and S2 in supplementary Section S23.

S2 Dispersion relation diagrams

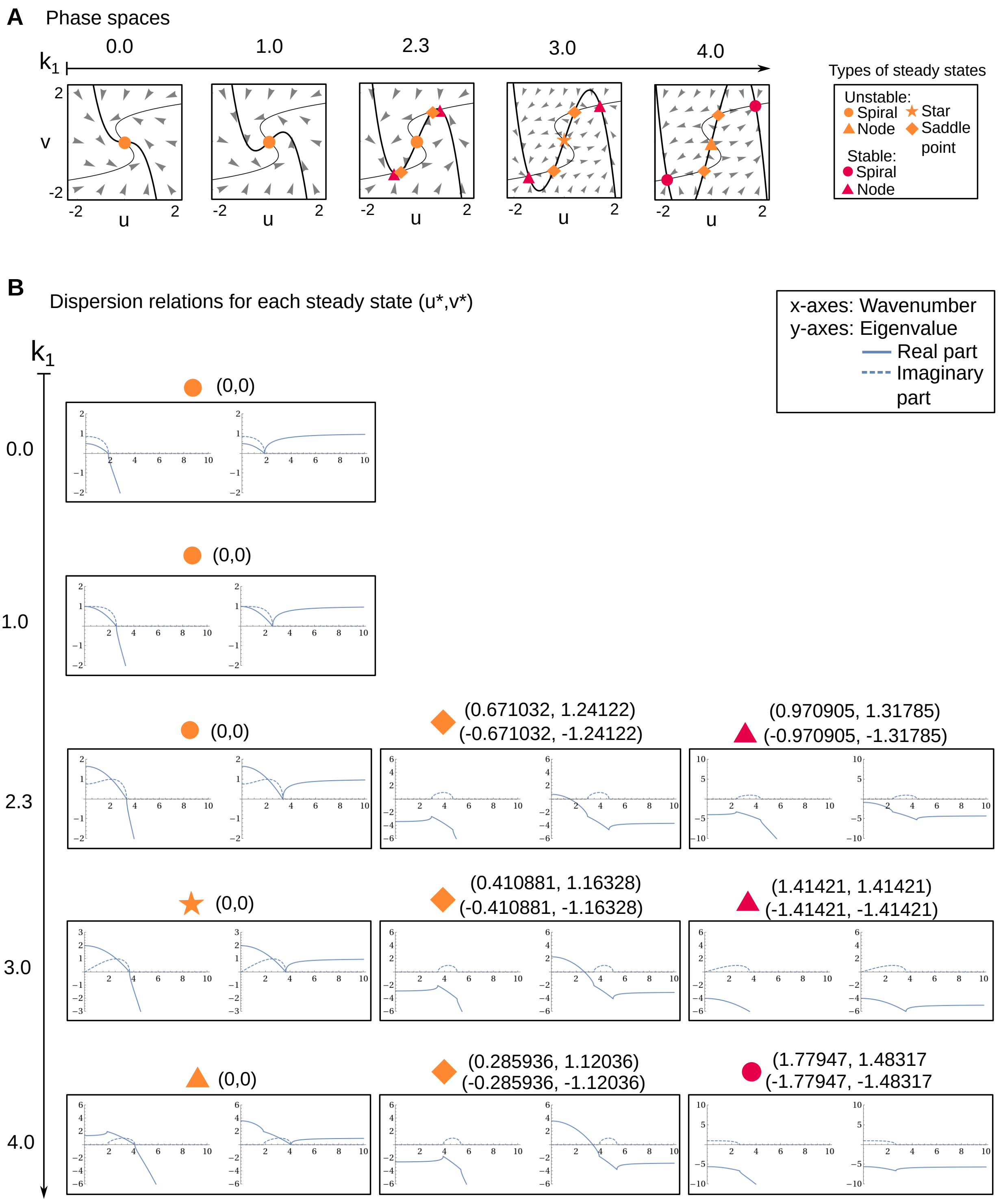

Fig. S2: (Caption on next page.)

Development • Supplementary information

Fig. S2: **Dispersion relation diagrams for the steady states of the example systems in Fig. 2E.**
A) Phase spaces (as shown in Fig. 2F).
B) Dispersion relations computed via linear stability analysis around the different fixed points of the Sevilletor equations for different values of k_1. The dispersion relation plots show the wavenumber on the x-axis and eigenvalue on the y-axis for each fixed point (u^*, v^*) (above the graphs). The real (solid line) and imaginary (dashed line) parts of the eigenvalues are plotted against the wavenumber (2π/wavelength). In the second and third columns for $k_1 => 2.3$, only two representative eigenvalues are displayed, as they are identical for both fixed points, indicating system symmetry. For wavenumber>0, a positive real part of the eigenvalue denotes Turing instability. If the real part of the eigenvalue is positive at wavenumber=0, the system is unstable without diffusion; conversely, if negative, the system is stable without diffusion. The rightmost columns for $k_1 \geq 2.3$ demonstrate the absence of Turing instability from the two stable points in the system (negative real eigenvalues for wavenumber>0).

Development • Supplementary information

S3 Oscillations, excitability and bistability in previous models

Spatio-temporal wave patterns are a classical phenomenon observable in systems with an oscillatory type III behavior (Cross and Hohenberg, 1993). In these systems, waves propagate through the spatial synchronization of oscillations originating from an unstable fixed point (Fig. S3A) (Novák and Tyson, 2008). In the case of chemical systems, synchronization can be mediated by diffusion. Depending on the initial conditions and the trajectory around the unstable point, these systems can generate traveling waves, target patterns, or spiral patterns (Cross and Hohenberg, 1993).

However, wave patterns can also emerge from stable fixed points that can be destabilized in response to a large stimulus, triggering a temporary deviation from the system's equilibrium state (Fig. S3B), a behavior referred to as excitability. When excitable systems are spatially coupled, for example, through diffusion, a stimulus induced by a localized perturbation can propagate in space in the form of a wave pattern. The type of wave patterns formed depends on the type of perturbation and on the trajectory around the fixed point, which is determined by the nonlinearity and feedback in the system.

Finally, wavefront patterns are also observed in bistable systems, which in the simplest case exhibit two stable states separated by an unstable equilibrium (Fig. S3C). In these systems, wave propagation usually occurs as a transition from one stable state to another, which, similarly to the excitability case, can be triggered by a large local perturbation propagating as a wavefront at the interface between the two states.

Systems involving reaction-diffusion or reaction-convection terms, such as the FitzHugh-Nagumo model (FitzHugh, 1961), the Oregonator (Cross and Hohenberg, 1993), Brusselator (Prigogine and Lefever, 1968), or the Complex Ginzburg-Landau equation (Tyson and Keener, 1988; Aranson and Kramer, 2002), can undergo a bifurcation from an oscillatory to a bistable behavior and exhibit all the different wave patterns presented above. Bifurcations in these models can arise by changing a single parameter, promoting a phase space change similar to the transitions between Figure S3. In the majority of studies, however, from the bistable regime these models give rise to wavefronts triggered by local perturbations that can spread or dissipate one of the two stable states across space. An exception is the case of the discrete version of the Fitzhugh-Nagumo model simulated in a 2D lattice in Shepelev and Vadivasova (2019), where several types of wave patterns in a bistable regime occur due to coupling between the cells in the lattice.

The difference between the Sevilletor equation and previous models lies in the shape of the phase-space in the bistable regime. In the classical models discussed above, the bistable regime is typically characterized by three fixed points observed in previous models (Fig. S3C), comprising a central unstable point and two peripheral stable points. In the Sevilletor equation, upon bifurcation (for $k_1 \geq 2.3$), the system exhibits 5 fixed points: a central unstable point and two peripheral stable points with two unstable points in the vicinity, as shown in Figure S4B. This phase space is identical to the one presented in recent models of body segmentation (François et al., 2007; Jutras-Dubé et al., 2020). In this study, we characterized for the first time the patterning behavior that this phase portrait exhibits in the presence of diffusion. In particular, we showed that diffusion between neighboring cells in opposite phases can generate a diffusion-driven excitable oscillation (see Figure S4B). A convenient feature of the Sevilletor model is that a progressive change in the strength of k_1 in the model not only promotes a bifurcation from oscillation to bistability but it also controls the distance between stable and unstable points within the bi-stability regime (see Figure S4B-C), which determines the excitability of the system.

Development • Supplementary information

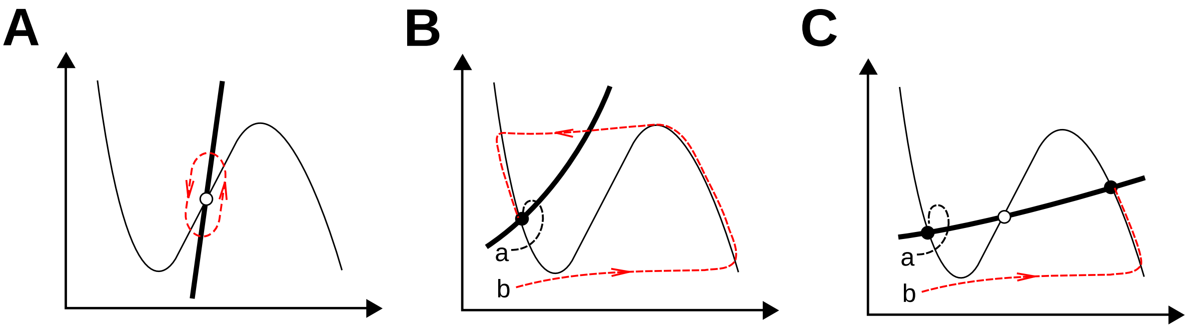

Fig. S3: **Schematic representation of the dynamical regimes that lead to wave formation in the Oregonator model, based on figure 78 in Cross and Hohenberg (1993).** Thick and thin lines depict nullclines of two species, black dots represent stable states, and white dots indicate unstable states. Dashed lines illustrate trajectories in phase space.
A) Starting from a single unstable point (white dot), the system exhibits type IIIo oscillatory behavior with a limit cycle (red dashed line). Synchronization of oscillations in space by diffusion can lead to traveling waves, target patterns, or spiral patterns.
B) The system can also exhibit excitability from a stable point (black dot), where small perturbations (a) quickly return the system to equilibrium (black dashed line), while large perturbations (b) temporarily deviate the system from equilibrium before returning to the fixed point (red dashed line). In spatially coupled systems by diffusion, large local perturbations can propagate as wave patterns.
C) In a bistable regime characterized by two stable points (black dots) and a central unstable point (white dot), small perturbations (a) rapidly return the system to equilibrium (black dashed line), while large perturbations (b) induce temporary deviations before returning to a fixed point (red dashed line). In spatially coupled systems by diffusion, large local perturbations can propagate as wave patterns.

The diffusion-driven oscillation promoted from the bi-stable Sevilletor regime is also not a Turing diffusion-driven instability. Indeed, the dispersion relation starting from the two peripheral stable points show no positive real eigenvalues, see two columns on the right for $k_1 = 2.3$ in Fig. S2B.

S3.1 Models of the Belousov–Zhabotinsky reaction

The pattern generated by the Sevilletor model near the bifurcation point ($k_1 = 2.3$) looks similar to those formed by classic models of the Belousov–Zhabotinsky reaction (Zhabotinsky and Zaikin, 1973) such as the Brusselator (Prigogine and Lefever, 1968) (equations (S3) and (S4)) and Oregonator (Field and Noyes, 1974). However it emerges from a different dynamical behavior. The classic models (Prigogine and Lefever, 1968; Field and Noyes, 1974) generate spiral patterns based on a limit cycle around a single unstable steady state as shown in Fig. S5B, similar to the rotating pattern presented in this paper with $k_1 = 1$ (Fig.3C). In contrast, the periodic wave patterns with spirals formed by the Sevilletor model with $k_1 = 2.3$ arises from a diffusion-driven excitation of a bistable regime which includes three unstable states (Fig. 3D and Movie 3).

Brusselator equations (Prigone, 1978):

$$\frac{\partial X}{\partial t} = A + X^2Y - (B+1)X + D_X\nabla^2 X, \tag{S3}$$

$$\frac{\partial Y}{\partial t} = BX - X^2Y + D_Y\nabla^2 Y. \tag{S4}$$

We have simulated periodic wave patterns containing spirals with the Brusselator equations with the parameters found in the paper by Torabi and Davidsen (2019): $A = 1.9$, $B = 4.8$, $D_X = 1.0$

Development • Supplementary information

and $D_Y = 0.7$, $L = 256$. We set the initial values around $(X, Y) = (1.9, 2.52632)$ and used zero flux boundaries.

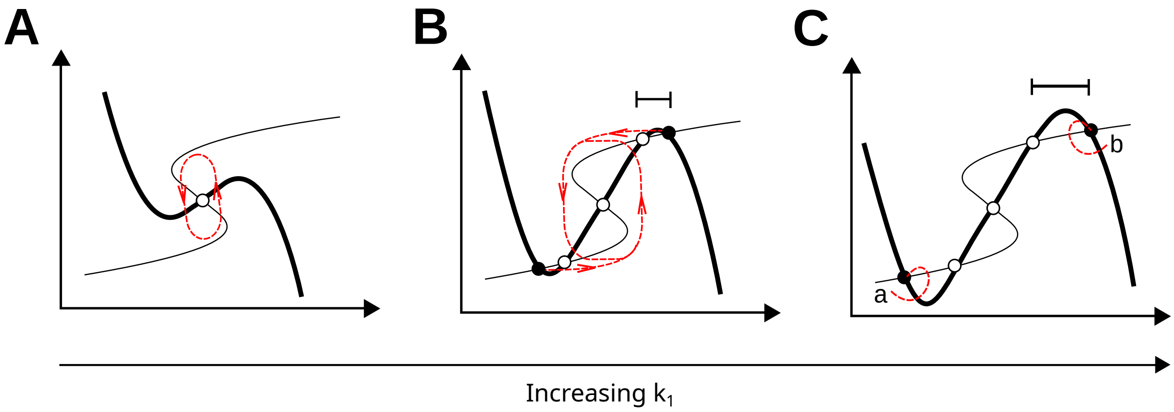

Figure S4: **Schematic representation of the dynamical regimes that lead to wave formation in the Sevilletor model.** Thick and thin lines depict nullclines of two species, black dots represent stable states, and white dots indicate unstable states. Dashed lines illustrate trajectories in phase space.
A) Starting from a single unstable point (white dot), the system exhibits type IIIo oscillatory behavior with a limit cycle (red dashed line). Synchronization of oscillations in space by diffusion can lead to rotatory wave patterns (shown in second row Fig. 2E).
B) Due to the non-linearity of the system, in the bi-stable regime the Sevilletor equations have 5 fixed points, a central unstable (white dot), and two peripheral stable points (black dots), each in the vicinity of an unstable point (white dots). When cells in opposite phases are coupled by diffusion, the system is excited out of equilibrium to generate a diffusion-driven limit cycle (red dashed line). This happens even in the absence of perturbations starting from the bi-stable points.
C) When the distance between the peripheral stable and unstable points (distance bar) is large (for larger values of k_1), the bi-stable states cannot anymore be destabilized by diffusion between cells in opposite phases and the system behaves as a classical bi-stable system.

Our analysis shows that in the Brusselator model, spirals are formed out of local heterogeneities in the initial conditions. Analysis of the phase space shows that these neighboring cells follow a spiral trajectory towards the central steady state, temporarily transforming the unstable steady state of the system into an effectively stable one. Following this trajectory, neighboring cells reach the steady state and eventually synchronize their phases following the original limit cycle. This event is associated with the disappearance of spiral centers. These initial spiral centers are formed independently of boundary conditions and are required to produce a stripy phase pattern.

In contrast, with $k_1 = 2.3$, the Sevilletor model generates spirals that appear and disappear dynamically over time while moving in space (Movie 1). Numerical simulations show that these spirals are formed only with zero flux boundary conditions, suggesting that the spiral appearance is due to crashing of phase waves reflected by zero flux boundary conditions. With periodic boundary conditions, the long term behavior of the system is to generate a periodic phase wave pattern of straight stripes (Fig.S6).

Development • Supplementary information

S3.2 Van der Pol

The Sevilletor equations build upon another set of equations that also form rotating patterns from an unstable state: the Van der Pol equations (S5) and (S6) shown in Fig. S5A. The Sevilletor extends the Van der Pol equations with an additional self-enhancing feedback and cubic saturation term in equation (S6).

Van der Pol equations (FitzHugh, 1961):

$$\frac{\partial X}{\partial t} = c(X - X^3/3 + Y) + D_X \nabla^2 X, \tag{S5}$$

$$\frac{\partial Y}{\partial t} = -X/c. \tag{S6}$$

We have simulated rotating wave patterns with the Van der Pol equations with the parameters: $D_X = 0.3$, dt=0.002, $c = 1$, $L = 100$, with initial noise around $(X, Y) = (0, 0)$ and zero flux boundaries.

The Sevilletor equations: Equations (1) and (2) repeated for convenience:

$$\frac{\partial u}{\partial t} = k_1 u - v - u^3 + D\nabla^2 u,$$

$$\frac{\partial v}{\partial t} = v + u - v^3.$$

We simulate rotating waves with $k_1 = 1.0$ and periodic wave patterns with $k_1 = 2.3$. $D = 0.3$, $L = 100$. The initial values are around $(u, v) = (0, 0)$ and the system has zero flux boundaries.

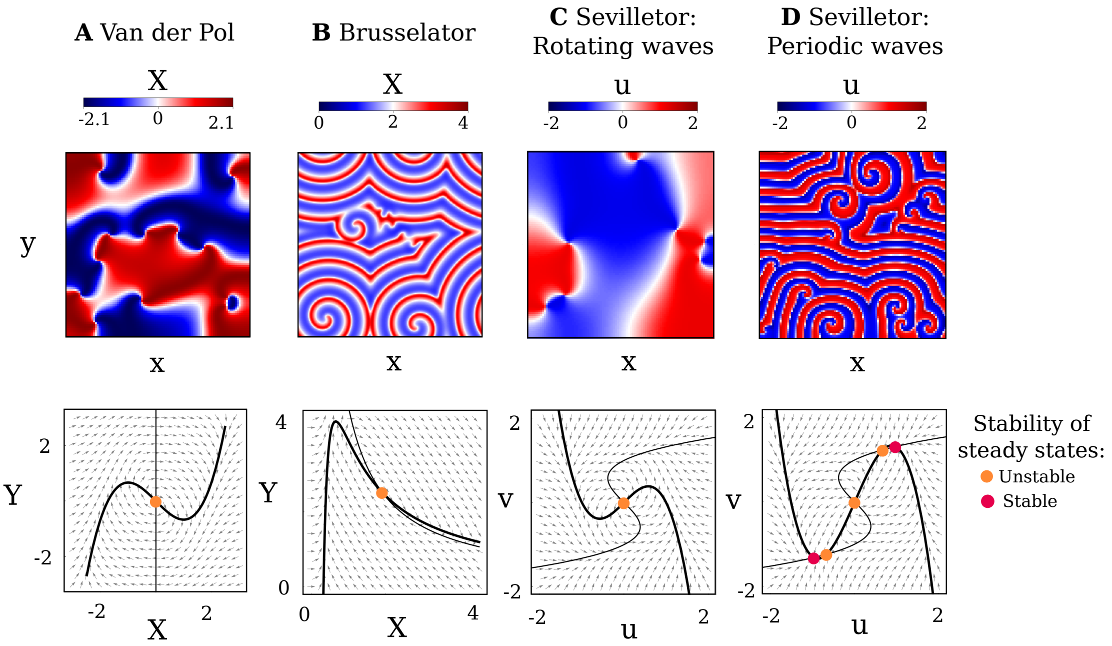

Fig. S5: **Models that produce rotating wave patterns.**
Simulations of rotatory wave formation (top row) and corresponding phase spaces (bottom row). The Van der Pol (FitzHugh, 1961) (A), Brusselator (Prigone, 1978; Torabi and Davidsen, 2019) (B) and Sevilletor rotating waves (C) have a single unstable steady state and a limit cycle in the phase space. The Sevilletor periodic wave excitable regime (D) has five steady states, including two stable states. However curiously, visually the Van der Pol is similar to (C), and the Brusselator similar to (D).

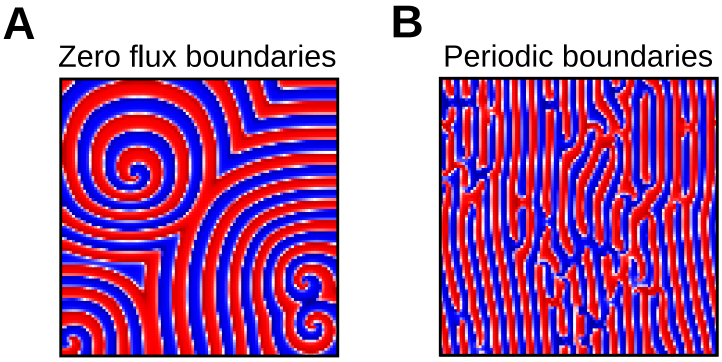

Fig. S6: **The Sevilletor excitable regime creates straight traveling phase waves in systems with periodic boundaries.** $k_1 = 2.3$ and $D = 0.3$. Plots are shown for u.
A) Zero flux boundaries allows for crashing of phase waves that creates spiral centers.
B) A system with periodic boundaries creates phase waves that travel from right to left, exiting the simulation on the left boundary and re-entering the simulation on the right boundary. All parameters are shown in Tables S1 and S2 in supplementary Section S23.

S3.3 FitzHugh-Nagumo model

Like the Sevilletor model, another model that was also developed from the Van der Pol equations is the famous FitzHugh–Nagumo model, proposed by FitzHugh in 1961 (FitzHugh, 1961) to study neuronal firing. In this model, an additional excitation term in equation (1) and a negative linear term in equation (2) was added to investigate how a stable system could be excited to become unstable. Unlike the FitzHugh–Nagumo model, the Sevilletor model always has an instability in the steady state $(u^*, v^*) = (0, 0)$ that is driven by the two positive self-enhancing feedbacks of u and v. Moreover, while the FitzHugh–Nagumo model can have only up to three steady states, due to the linear shape of the v nullcline, in the Sevilletor model both v and u have nullclines with non-linear shapes that can intersect on more than three points, which is fundamental to give rise to the excitable bistable behavior.

Development • Supplementary information

S4 Initial phase values determine the spatial synchronizations

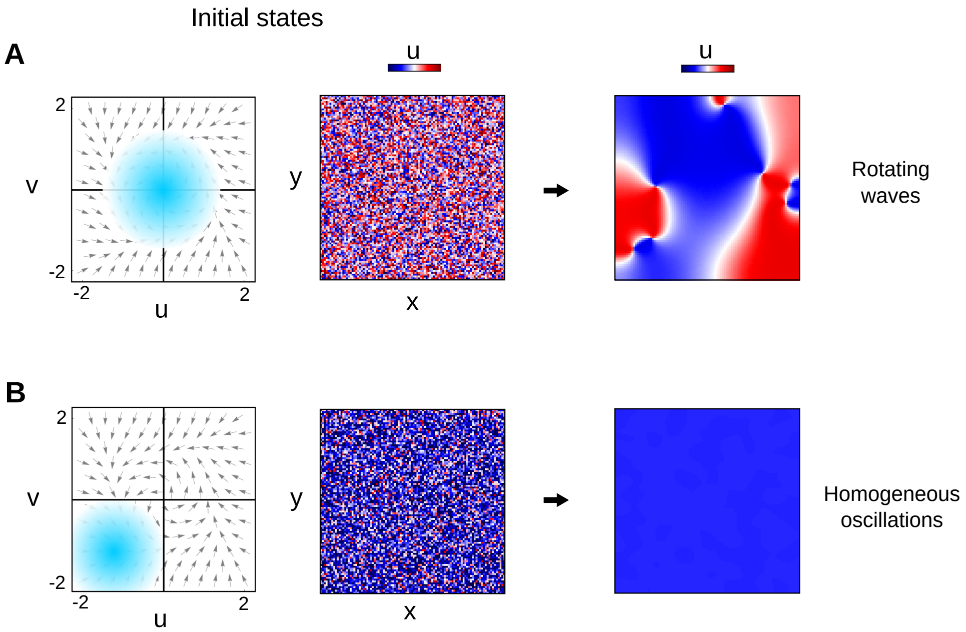

Fig. S7: **Spatial synchronizations are determined by initial concentrations of u and v.** The self-organizing patterning formed by the Sevilletor equations with $k_1 = 1$ is influenced by the half-planes where initial conditions lie. Left column: initial concentrations illustrated in cyan area of the phase space (steady states are not shown). Middle column: Corresponding initial conditions for the 2D simulation. Right: Result of the corresponding 2D simulation.
A) Uniformly distributed random initial concentrations around $(u, v) = (0, 0)$ that lie both in lower and upper half planes give rise to rotating wave patterns.
B) Uniformly distributed random initial concentrations around $(u, v) = (-1, -1)$ that lie only on the lower half plane give rise to homogeneous oscillations. The same result can be obtained with initial conditions that lie in the upper half plane. All parameters are shown in Tables S1 and S2 in supplementary Section S23.

Development • Supplementary information

S5 The self-regulatory delayed negative feedback of Notch can be coupled with a negative feedback between Wnt and Notch without changing the oscillatory behavior

In the majority of classical somitogenesis models, oscillations are driven by a cell autonomous transcriptional feedback of Notch mediated by repressors of the Hes/Her family (Takashima et al., 2011; Ay et al., 2013). This transcriptional inhibition can be implemented with a delayed negative feedback that generates sustained oscillations (Lewis, 2003) (Fig. S8A):

$$\frac{\partial \texttt{Notch}}{\partial t} = -\texttt{Notch}_{delay}(\tau - 15) \tag{S7}$$

In the Sevilletor equations, we assume that the additional negative feedback that couples Wnt and Notch complements the core delayed negative feedback of Notch. This additional feedback is implemented by two simple linear interactions with opposite sign and parameters k_3 and k_4, generating an effective delayed negative feedback that gives rise to out of phase oscillations of Wnt and Notch (Fig. S8B).

$$\frac{\partial \texttt{Wnt}}{\partial t} = \texttt{Wnt} - k_3\texttt{Notch} - \texttt{Wnt}^3 + D\nabla^2\texttt{Wnt} \tag{S8}$$

$$\frac{\partial \texttt{Notch}}{\partial t} = \texttt{Notch} + k_4\texttt{Wnt} - \texttt{Notch}^3 - \texttt{Notch}_{delay}(\tau - 25) \tag{S9}$$

The delayed negative feedback that couples Wnt and Notch is sufficient to drive oscillations (Fig. S8C). Together with the self-enhancement, this feedback generates self-organizing patterns due to synchronizations mediated by diffusion, see Fig. 2 and Fig. 3. To maintain the model complexity to a minimum, we consider this reduced self-organizing model without explicitly including the direct delayed negative feedback of Notch:

$$\frac{\partial \texttt{Wnt}}{\partial t} = \texttt{Wnt} - k_3\texttt{Notch} - \texttt{Wnt}^3 + D\nabla^2\texttt{Wnt} \tag{S10}$$

$$\frac{\partial \texttt{Notch}}{\partial t} = \texttt{Notch} + k_4\texttt{Wnt} - \texttt{Notch}^3 \tag{S11}$$

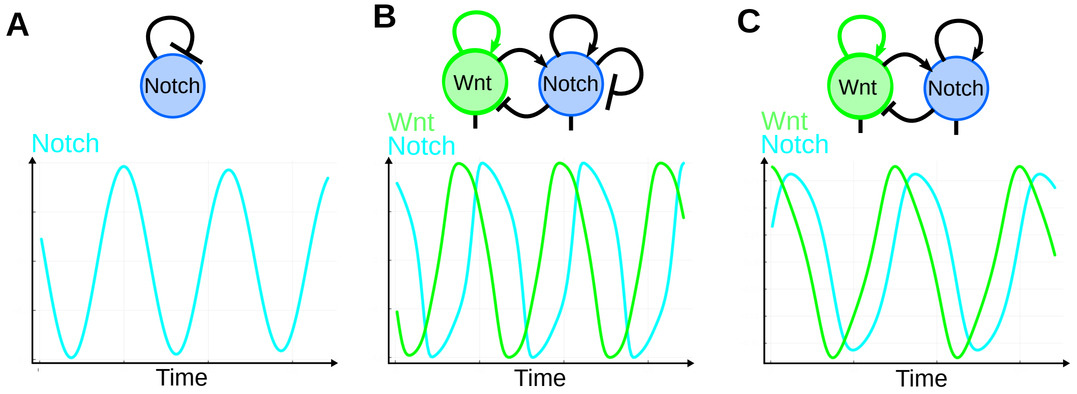

Fig. S8: **The Sevilletor includes a delayed negative feedback between Wnt and Notch, complementing the direct delayed negative feedback of Notch.**
A) A delayed negative self-regulation of Notch gives rise to oscillations.
B) Including an additional delayed negative feedback that couples Wnt and Notch as in the Sevilletor network does not affect the oscillatory behavior of the system ($k_3 = k_4 = 1$).
C) The delayed negative feedback that couples Notch and Wnt is sufficient to drive oscillations with a phase shift of Wnt and Notch ($k_3 = k_4 = 1$).

S6 Oscillations of coupled oscillators and with a negative feedback

The oscillatory regime in the posterior tail for $k_1 = 1$ in the Sevilletor implementations of the CG and CWS model is an emergent phenomenon driven by the negative feedback between u and v, leading to sustained cell-autonomous oscillations, equivalent to phase oscillators (Morelli et al., 2009; Herrgen et al., 2010; Jörg et al., 2016) (Fig. S9) or those in delayed negative feedback models (Lewis, 2003). If cells are coupled along the anterior-posterior axis by diffusion with $D = 0.3$, they exhibit robust collective oscillations resistant to noise. Starting from random phases, the coupling of oscillations forms rotating phase wave patterns, similar to pinwheel waves in Kuramoto models (Breakspear et al., 2010) as in the CG model in Uriu et al. (2021).

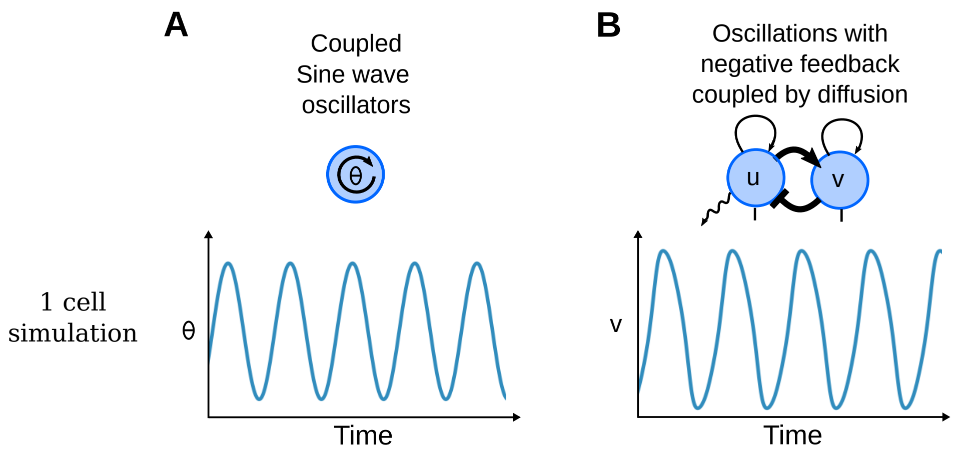

Fig. S9: **Oscillations of a single cell for coupled oscillators (Uriu et al., 2021) and for a negative feedback with the Sevilletor equations**.
A) Previous CG models coupled the phases of single cell oscillators represented by a phenomenological Sine wave function that represent sustained oscillations (Morelli et al., 2009; Herrgen et al., 2010; Jörg et al., 2016). Starting from heterogeneous phase values, 2D simulations of coupled oscillators give rise to rotating wave patterns (Uriu et al., 2021).
B) A negative feedback between two reactants coupled with positive feedbacks generates sustained oscillation. When these oscillations are coupled in space by diffusion, they give rise to rotating wave patterns, as shown on the right of Fig. 2E with $k_1 = 1$ started from noise, which is equivalent to to pinwheel waves in Kuramoto models (Breakspear et al., 2010) and the simulation from random phase values of the CG model presented in Uriu et al. (2021).

Development • Supplementary information

S7 Parameter space that gives rise to periodic wave patterns

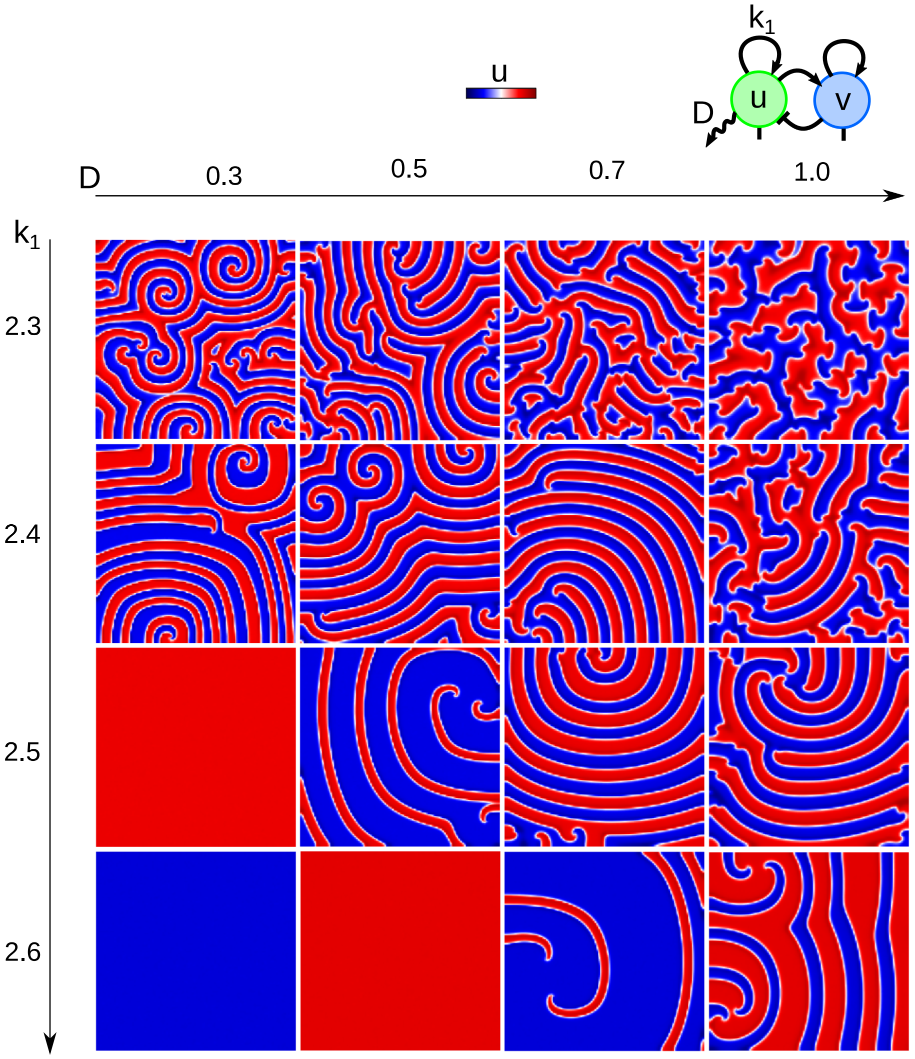

Fig. S10: **Parameter values of k_1 and D that give rise to periodic wave patterns**. As showed in Fig. 3D, periodic wave patterns arise near the bifurcation point between oscillations and bistability ($k_1 = 2.3$), where diffusion can destabilize the system to generate a new limit cycle. This behavior is possible for a broad range of values of $k_1 \geq 2.3$ provided that the destabilization by diffusion is strong enough $D \geq 0.3$. All parameters are shown in Tables S1 and S2 in supplementary Section S23.

Development • Supplementary information

S8 Spatial discretisation

The Sevilletor model is based on a discretization of space where the diameter of one cell is dx=1. As shown in Wang et al. (2022), how space is discretized affects the outcome of the simulation. In Fig. S11 we show that the two patterning behaviors that form excitable waves and lateral inhibition patterns preform equivalently with re-scaling the sizes of the cells dx when also scaling the diffusion constant D with a constant ratio $\sqrt{D}/\mathtt{dx}$, similar to the relation between wavelength and diffusion constant found in Wang et al. (2022). This proportion comes from the calculations of how diffusion distributes u in the system with a factor $D/\mathtt{dx}^2$ (see equations (10) and (16) in the Methods Section). In addition, while lateral inhibition requires a specific dx and D ratio (green squares along the diagonal in Figure S11B), we observe that excitable wave formation is robust to changes in the ratio between dx and D (green squares in Figure S11A) requiring only that D is sufficiently large relative to dx.

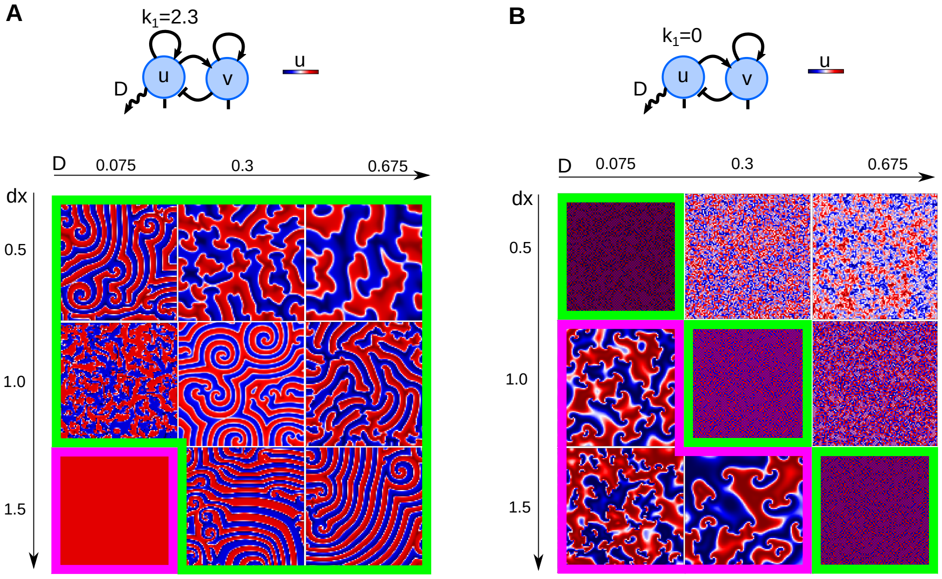

Fig. S11: **Pattern formation with variations of spatial discretisation (size of dx = dy) and diffusion constant D. A)** Excitable behavior with $k_1 = 2.3$. **B)** Lateral inhibition behavior with $k_1 = 0$. The size of each system is set to $L_x = L_y = 100\mathtt{dx}$ to be able to see the patterns. Green edges mark the behaviors similar to the ones observed throughout the paper with standard spatial discretisation and diffusion (middle squares), and magenta edges mark the behaviors that differ significantly from the standard. Both patterns perform equivalently with proportional values of dx and D with a constant ratio $\sqrt{D}/\mathtt{dx}$ (diagonal from upper left to bottom right).
A) Excitable phase waves are robust to changes in the ratio between dx and D (green squares), with variations in wavelengths and shapes of the pattern. An exception is when diffusion is not strong enough to excite large cells when dx increases too high relative to D, creating a static homogeneous pattern (magenta square).
B) Lateral inhibition patterning is sensitive to changes in the dx and D ratio, with only the ratio $\sqrt{D}/\mathtt{dx}$ creating a chessboard pattern (green squares). The wavelength changes with the size of the cells (dx), because one wavelength is equal to two cells, as in the pattern observed in Wang et al. (2022). A static disorganized pattern is formed when diffusion is high relative to dx (upper right corner), while waves are formed when diffusion is too low (relative to the cell size) to freeze the cells in phase space (magenta lower left corner). This change in behavior differs from the one observed in Wang et al. (2022) which is more robust to variations in the diffusion constant. A reason could be that it is because cells are by default in a stable state, while in the Sevilletor equations the system has only an unstable state (see Figure 2F first row).
All parameters are shown in Tables S1 and S2 in supplementary Section S23.

All three models implemented with the Sevilletor equations (PORD, CG, CWS) also preform equivalently with re-scaling the sizes of the cells `dx` and the diffusion constant D with a constant ratio $\sqrt{D}/\mathtt{dx}$ (Fig. S12). How the discretization relates to real units is discussed in the Supplementary Section S22 below.

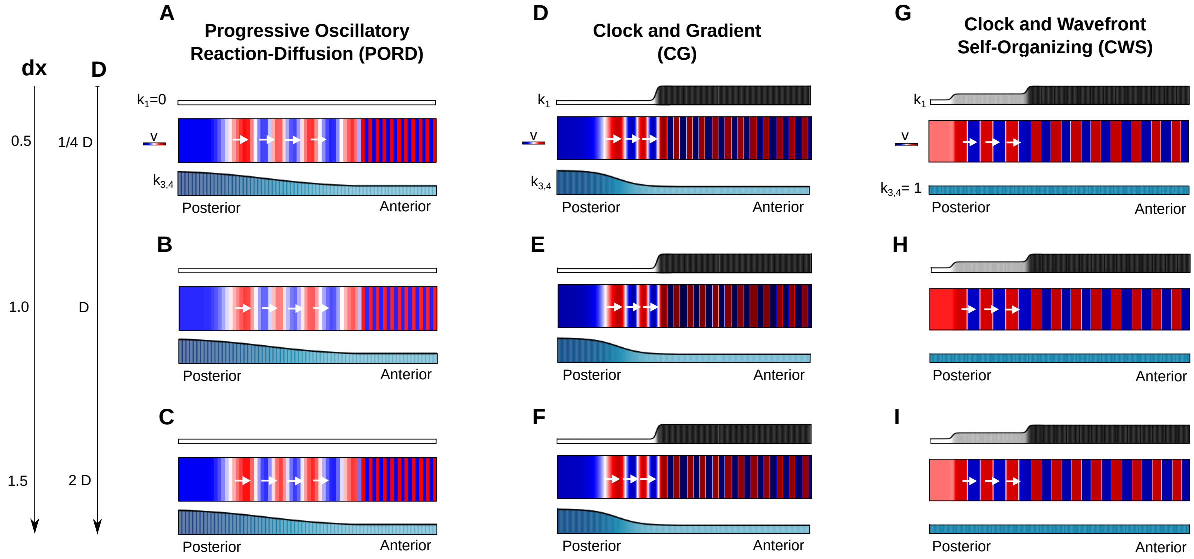

Figure S12: **Somitogenesis models implemented in the Sevilletor framework perform equivalently with re-scaled values of spatial discretisation and diffusion constant** D. The models are robust to changes with proportional values of `dx` and D with a ratio $\sqrt{D}/\mathtt{dx}$.
A) The Sevilletor implementation of the PORD model with $D = 1$.
B) The Sevilletor implementation of the CG model with $D = 0.3$.
C) The CWS model with $D = 0.3$.
All parameters are shown in Tables S3 and S4 in supplementary Section S23.

Development • Supplementary information

S9 The original PORD model by Cotterell et al. (2015)

In the original PORD model by Cotterell et al. (2015) oscillations arrest in opposite phases as observed in the lateral inhibition patterning regime of the Sevilletor model, as shown by the two cell simulation of the PORD equations (S12) and (S13) in Fig. S13A. The cell starting in (0,0) makes one single loop around the steady state, before freezing at the bottom left corner while the other cell freezes at the top right corner. This is a non-symmetric phase space, while in our PORD implementation (Fig. S13B) the phase space and the path of the cells are symmetric, starting from opposite sides of the steady state. This difference, together with use of a heavy side function to prevent negative values, may explain why the periodic peaks formed by the original PORD model are separated by several cells, while in our model and in other implementations of the PORD hypothesis (Kuyyamudi et al., 2022) they are separated by only one cell. Despite this difference, all PORD implementations exhibit the same underlying behavior based on the freezing of oscillations in opposite phase due to diffusion between neighboring cells.

The equations of the original PORD model by Cotterell et al. (2015) are:

$$\frac{dA}{dt} = \frac{\Phi\big(k_1 A + k_2 R + F + \beta\big)}{1 + k_1 A + k_2 R + F + \beta} - \mu A, \tag{S12}$$

$$\frac{dR}{dt} = \frac{k_3 A}{1 + k_3 A} + D_R \nabla^2 R - \mu R, \tag{S13}$$

$\Phi(x) = x \cdot H(x)$, where $H(x)$ is a Heavyside function: $H(x) = 1$ for $x > 0$ and $H(x) = 0$ for $x \leq 0$. The parameters used in Figure S13A are the following: $k_1 = 1.56$, $k_2 = -2.28$, $k_3 = 0.099$, $\beta = 0.5$, $\mu = 0.05$, $F = 1$. $D_R = 0.008$.

The Sevilletor equations for the lateral inhibition behavior used to implement the PORD model are (equations (1) and (2) repeated for convenience):

$$\frac{du}{dt} = k_1 u - k_3 v - u^3 + D\nabla^2 u,$$

$$\frac{dv}{dt} = k_2 v + k_4 u - v^3.$$

With $k_1 = 0.0, k_2 = 1$ and $D = 1.0$. For the example shown in Fig. S13B we set the parameters of the frequency gradient `FG` to $k_3 = k_4 = 1$.

Development • Supplementary information

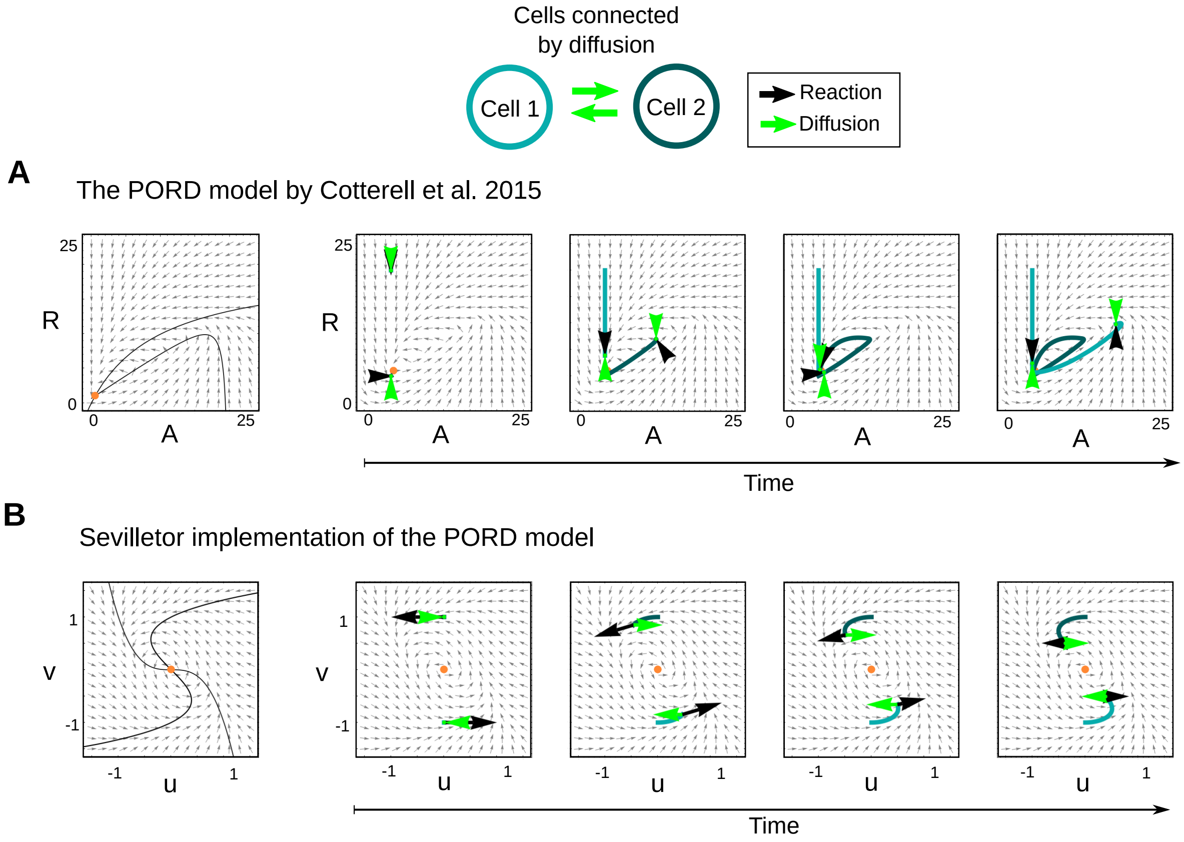

Fig. S13: **Two cell simulations of the original PORD model by Cotterell et al. (2015) and the Sevilletor implementation. A)** The original PORD model by Cotterell et al. (2015) and **B)** our implementation in the Sevilletor framework. See parameters above in Section S9. Left: Phase spaces with nullclines (black lines) and unstable steady states (orange). Right: The time series show the path of 2 cells with initial values: A) $(A, R) = (0, 20)$ and $(0, 0)$, B) $(u, v) = (0, 1)$ and $(0, -1)$. In both cases the cells freeze out of phase when reaching a point where changes promoted by reactions (black arrows) are counterbalanced by diffusion (green arrows), freezing cells in opposite state in both models.

In the original PORD model the change of frequency along the anterior-posterior axis is created with a gradient of the strength of the parameter F which enhances A. In the Sevilletor implementation of the PORD model we have implemented the frequency gradient as a graded modulation `FG` of the strength of the negative feedback between the two reactants (k_3 and k_4). As has been shown in Pantoja-Hernández et al. (2021), we also found that both the original PORD equations and the Sevilletor implementation are fragile to noise, as shown in Fig. S14.

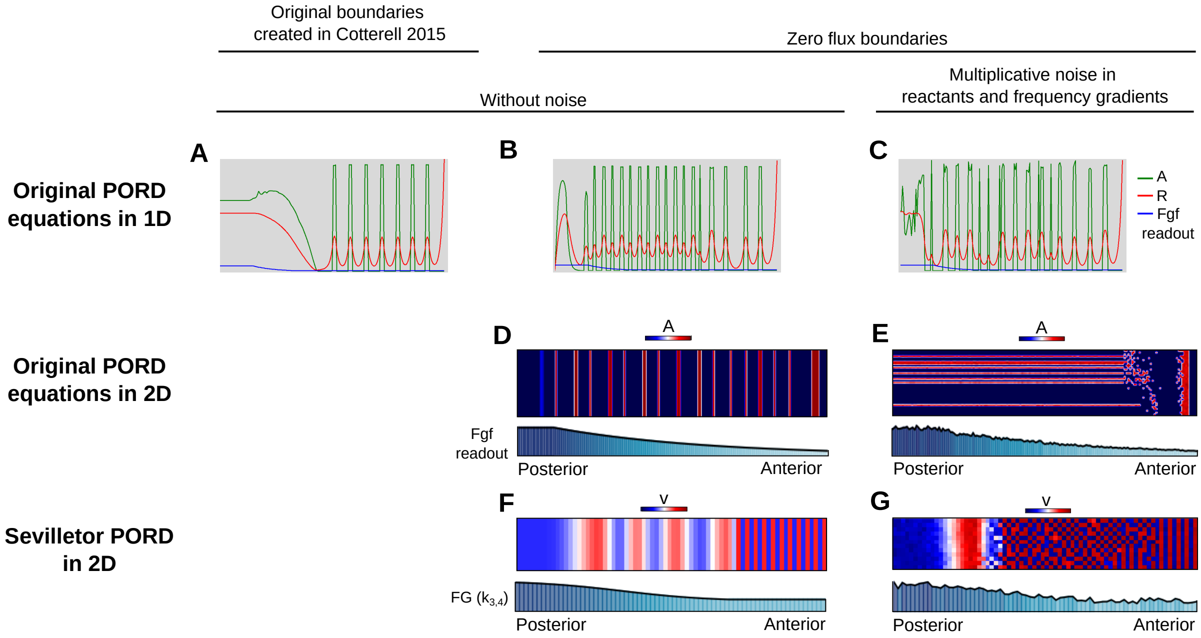

Fig. S14: **Noise in the PORD model creates a disrupted pattern.**
A-C) The original PORD model by Cotterell et al. (2015) in 1D (equations (S12) and (S13)). The parameters used are the following: $k_1 = 1.56$, $k_2 = -2.28$, $k_3 = 0.099$, $\beta = 0.5$, $\mu = 0.05$, F is a gradient from 1 to 0 and $D_R = 1.0$. An initial peak of R is placed at the most anterior line.
A) A regular striped pattern is formed with the original boundary conditions created by Cotterell et al. (2015) and without noise.
B) A striped pattern with variations in somite width is formed with zero flux boundary conditions and without noise.
C) An irregular striped pattern is formed with zero flux boundary conditions and with 5% multiplicative noise in the concentrations of A and R and 0.5% multiplicative noise in the gradient of Fgf readout (F).
D-E) The original PORD equations by Cotterell et al. (2015) in 2D (equations (S12) and (S13)).
D) A striped pattern with variations in somite width is formed with zero flux boundary conditions and without noise.
E) A disrupted pattern is formed with zero flux boundary conditions and with 5% multiplicative noise in the concentrations of A and R and 0.5% multiplicative noise in the gradient of Fgf readout.
F-G) The PORD model in the Sevilletor framework in 2D (Movie 6).
F) A regular striped pattern is formed with zero flux boundary conditions and without noise.
G) A disrupted pattern is formed with zero flux boundary conditions and with 5% multiplicative noise in the concentrations of u and v and 0.5% multiplicative noise in the frequency gradient `FG` (gradient of k_3 and k_4).

Development • Supplementary information

S10 Wnt patterns in models of virtual tails

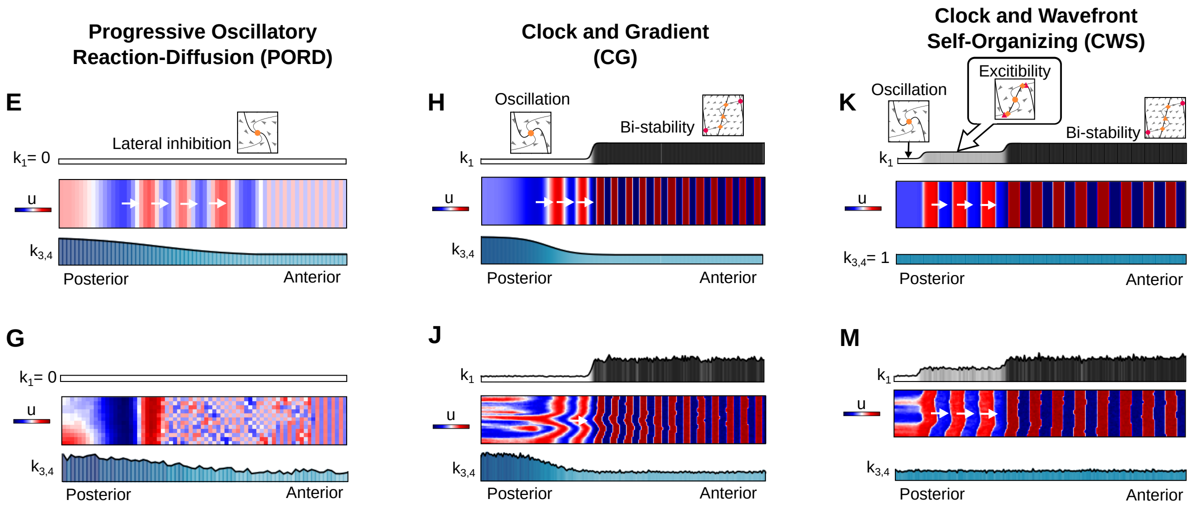

Fig. S15: **Somitogenesis models in Fig. 4 shown for u.** See the caption of Fig. 4.

Development • Supplementary information

S11 Fluctuating noise and cell movements

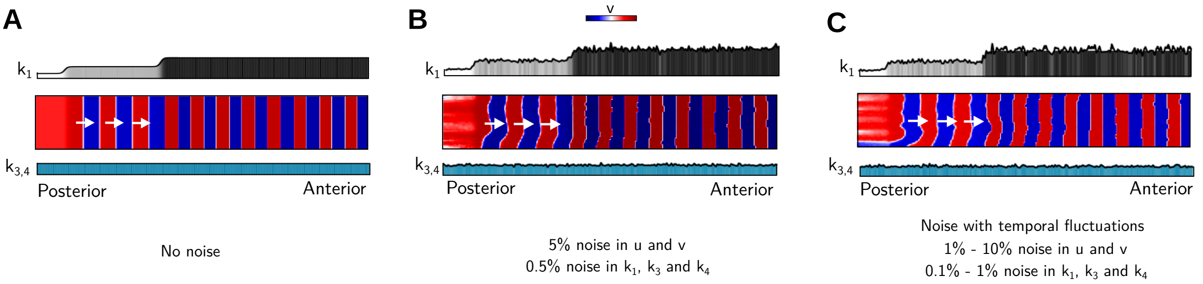

Fig. S16: **The CWS model is robust to fluctuating noise.** See the Methods Section for a detailed description of noise implementation. The patterns are shown for v.
A) The CWS model shown without noise.
B) The CWS model is robust to multiplicative noise in phase values and parameters. 5% multiplicative noise is added to u and v and 0.5% multiplicative noise is added to k_1, k_3 and k_4 (Movie 8).
C) Temporal fluctuations in the noise is introduced by generating noise with varying average amplitudes between 1-10% for u and v and 0.1-1.0% for k_1, k_3 and k_4. All parameters are shown in Tables S3 and S4 in supplementary Section S23.

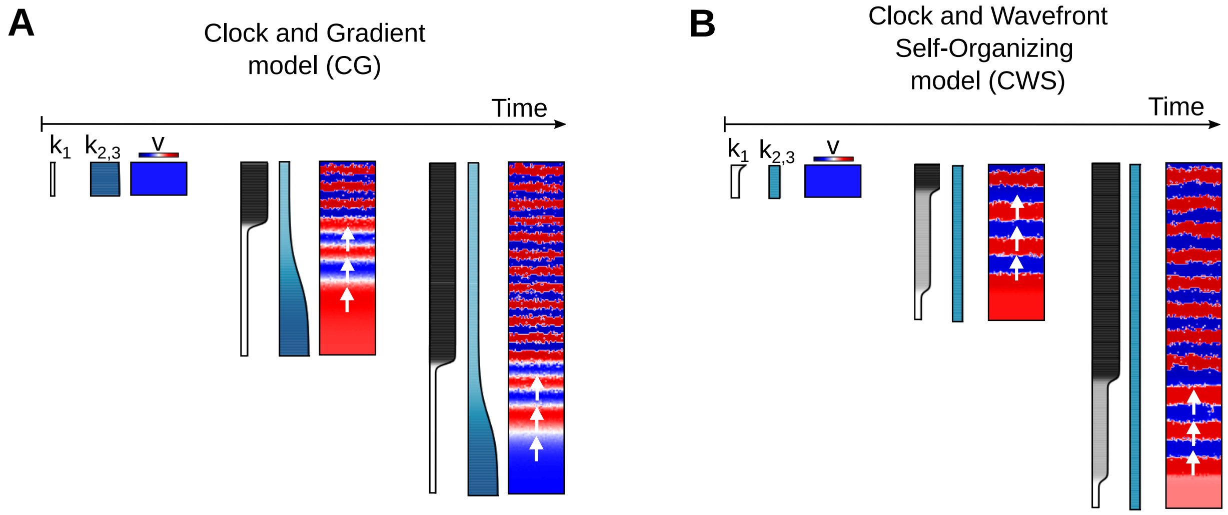

Fig. S17: **Sevilletor implementations of the CG and CWS models are robust to cell movement.** Movement is introduced as a swap of positions of pairs of neighboring cells with a probability $P_{swap} = 0.005$ for each cell at each time step. This results in about 500 swaps per time step in both models. Both the CG and the CWS are generally robust to cell movements in all three behaviors: oscillations (CG/CWS), excitability (CWS) and bistability (CG/CWS). However, as seen in the timelines, the oscillatory regions are less affected by the movement of the cells than the bistable regions. This is due to the fact that the cells can easily re-synchronize around an unstable steady state, while in a bistable regime the cells need to destabilize neighboring cells with noisy values of u and v to re-establish the organized pattern of straight lines. This fits with the experimental observations in Mongera et al. (2018), where cells have a more fluid-like behavior in the most posterior tailbud (MPZ) and a solid-like behavior in the rest of the PSM in Zebrafish. All parameters are shown in Tables S3 and S4 in supplementary Section S23.

S12 Varying tail width and the size of the tip

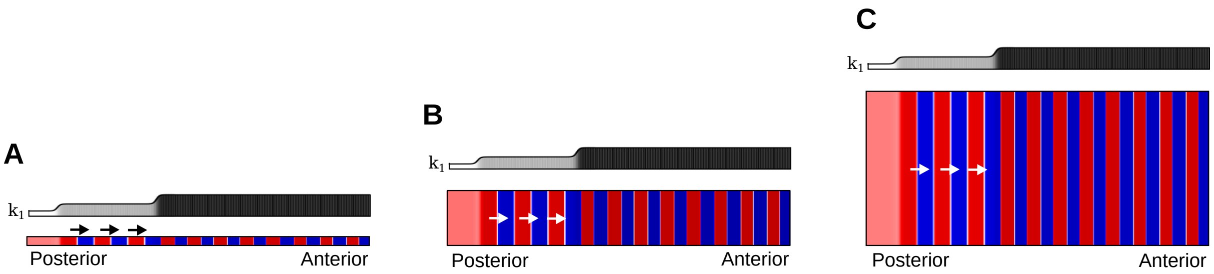

Fig. S18: **The CWS model is robust to changes in tail width**. The somitogenesis patterning of the CWS model does not depend on the width of the tail.
A) $L_y = 5$. **B)** $L_y = 35$. **C)** $L_y = 100$.
All parameters are shown in Tables S3 and S4 in supplementary Section S23.

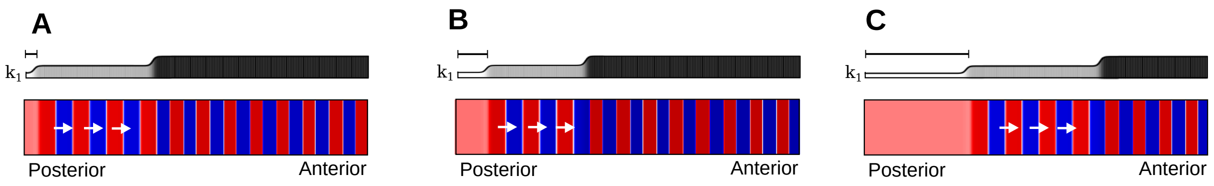

Fig. S19: **The CWS model is robust to changes in the length of the tip**. Phase waves are propagated in the excitable part of the tail, independent of the length of the oscillatory region in the tip. The simulations in (A) and (C) are perturbed from the standard CWS model in (B) by shifting the transition point from oscillatory to bistable, k_1: $1 \to 2.3$, and correspondingly the transition from the excitable to bistable, k_1: $2.3 \to 4$, to maintain the excitable area.
A) k_1: $1 \to 2.3$ at $x = 5$ and k_1: $2.3 \to 4$ at $x = 80$.
B) k_1: $1 \to 2.3$ at $x = 25$ and k_1: $2.3 \to 4$ at $x = 85$.
C) k_1: $1 \to 2.3$ at $x = 80$ and k_1: $2.3 \to 4$ at $x = 150$.
All parameters are shown in Tables S3 and S4 in supplementary Section S23.

Development • Supplementary information

S13 Dissection of the tailbud in the CWS model

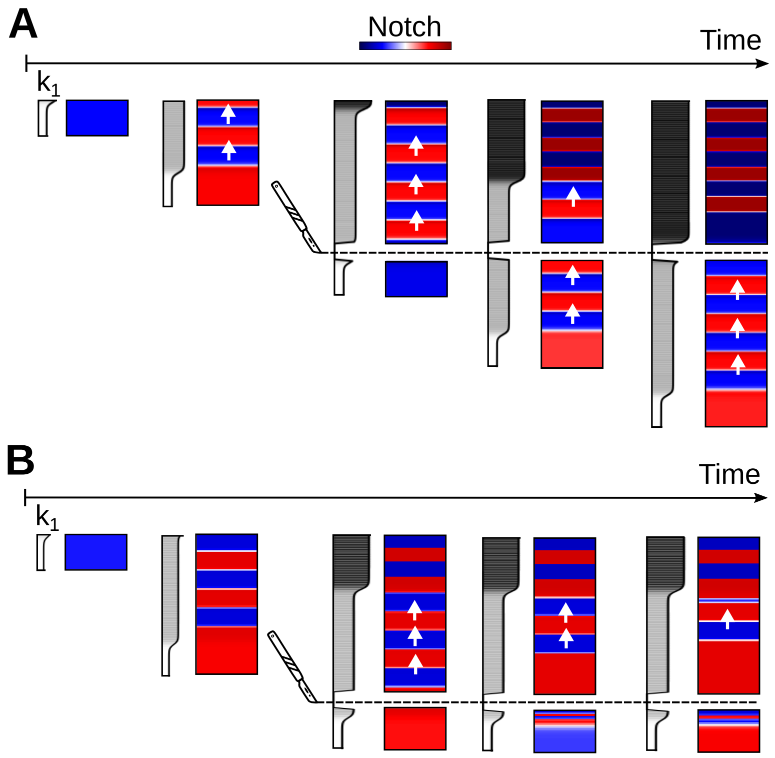

Fig. S20: **Virtual dissection of the tailbud in the CWS model (Movie 10)**. Time series showing the effect of tailbud dissection in the Clock and Wavefront Self-Organizing model.
A) Following the separation of the posterior region, the tailbud continues to grow and to generate new phase waves that travel anteriorly. In the anterior dissected part, preexisting phase waves persist and propagate anteriorly, driven by the excitable behavior in the intermediate region (light gray section), until they stop when entering the bistable region (dark gray section). These findings align with experimental observations reported in Özelçi et al. (2022). Importantly, in the anterior dissected part, the propagation of straight waves without rotational movements occurs independently of the homogeneous oscillations at the posterior tailbud tip.
B) The same propagation of pre-existing phase waves above the cut is seen when posterior progression of the bistable region is prevented. Even in the absence of growth the dissected tail bud keeps generating new phase waves. All parameters are shown in Tables S3 and S4 in supplementary Section S23.

Development • Supplementary information

S14 Extended posterior regions and gradients

Upon the constitutive stabilization of beta-catenin "beta-cat-lox(ex3)" in an experiment in Aulehla et al. (2008), high levels of nuclear beta-catenin are detected throughout the whole presomitic mesoderm (PSM) without an appreciable posterior-to-anterior protein gradient. This leads to anteriorly expanded high levels of Wnt and Fgf signaling, as shown by the quantification of Pea3 expression, a direct target of Fgf signaling (Roehl and Nüsslein-Volhard, 2001), and the direct target of Wnt signaling Axin2 (Jho et al., 2002) from Fig. 2a,a'e,e' in Aulehla et al. (2008). In both cases, posterior signaling gradients exhibit ectopic high levels that decrease abruptly at the anterior side (red lines), in contrast to the gentle decrease observed in controls (blue lines). These results are shown in Fig. S21. More phase waves were observed in tails with high levels of Pea3 and Axin2 than in the wild type.

Development • Supplementary information

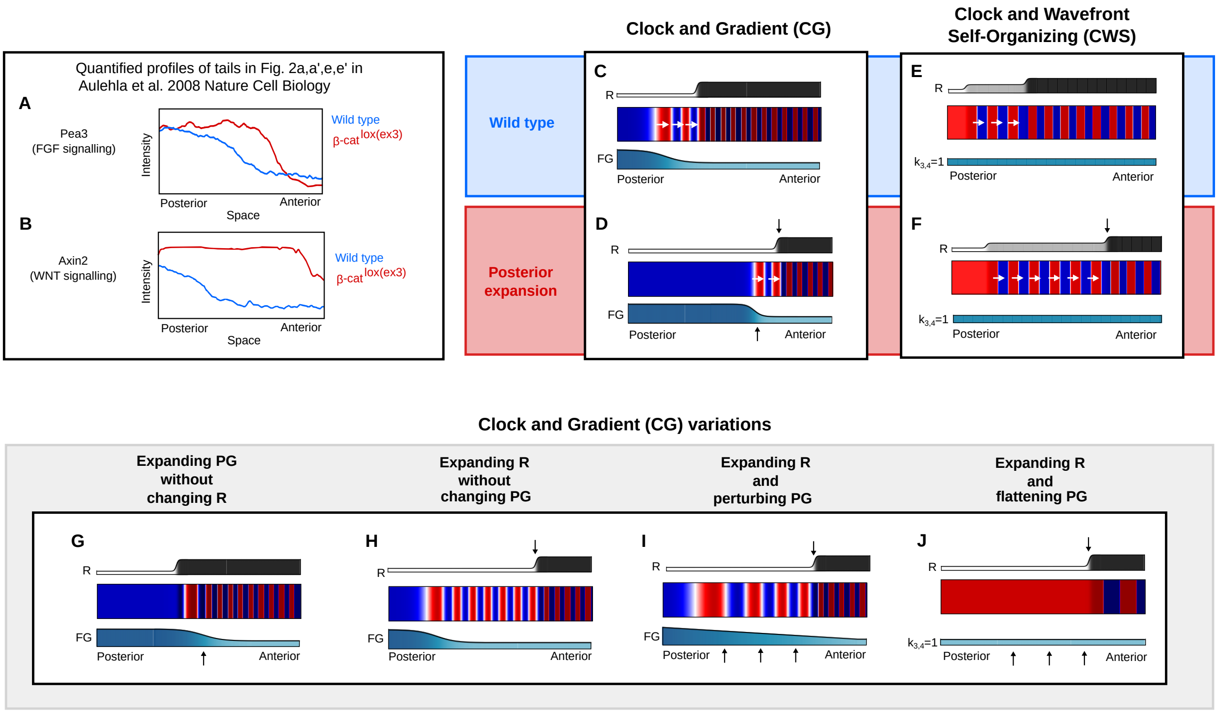

Fig. S21: **Expansion of posterior signals in Aulehla et al. 2008 Nature Cell Biology, and variations in the positions of R and FG in the CG and CWS models.**

A-B) Quantified profiles of the signals of Pea3 and Axin2 along the tails shown in Fig. 2a,a',e,e' in Aulehla et al. (2008) Nature Cell Biology, measured by converting the original images to an inverted grayscale image and measuring the intensity with straight profiles running from the posterior tip to the anterior side of the tail, done in Fiji (ImageJ).

C-D) Expanding R and FG anteriorly in the Sevilletor implementation of the CG model (D) to mimic the expansion and saturation of posterior signals observed in Aulehla et al. (2008) gives rise to a smaller section of phase waves with fewer waves in comparison to the wild type simulation (C).

E-F) Posteriorly expanding R in the CWS model (F) gives rise to a larger excitable section of the tail where an increased number of phase waves are formed with respect to the wild type simulation (E), in agreement with the experiment presented in Aulehla et al. (2008).

G-J) Variations of simulations by de-coupling R and FG in the Sevilletor implementation of the CG model.

G) When the graded modulation by FG is completely moved towards the anterior bistable region defined by R, the CG model does not form any phase waves.

H) Moving the bistable region (black) defined by R to the anterior side and leaving FG unchanged gives rise to multiple phase waves. Providing a possible scenario to interpret the phenotype observed in Aulehla et al. (2008) according to the CG model.

I) Moving the bistable region (black) defined by R to the anterior side and considering a linearly decreasing FG also gives rise to multiple phase waves.

J) No frequency gradient with a constant spatial value of k_3 and k_4 promoted by a flat FG predicts loss of phase waves. This case is equivalent to a basic Clock and Wavefront (CW) model. This differs from the CWS model (F) where multiple phase waves can be formed even with a constant spatial value of k_3 and k_4. All parameters are shown in Tables S3 and S4 in supplementary Section S23.

Development • Supplementary information

S15 Variation of diffusion parameters in the CWS model

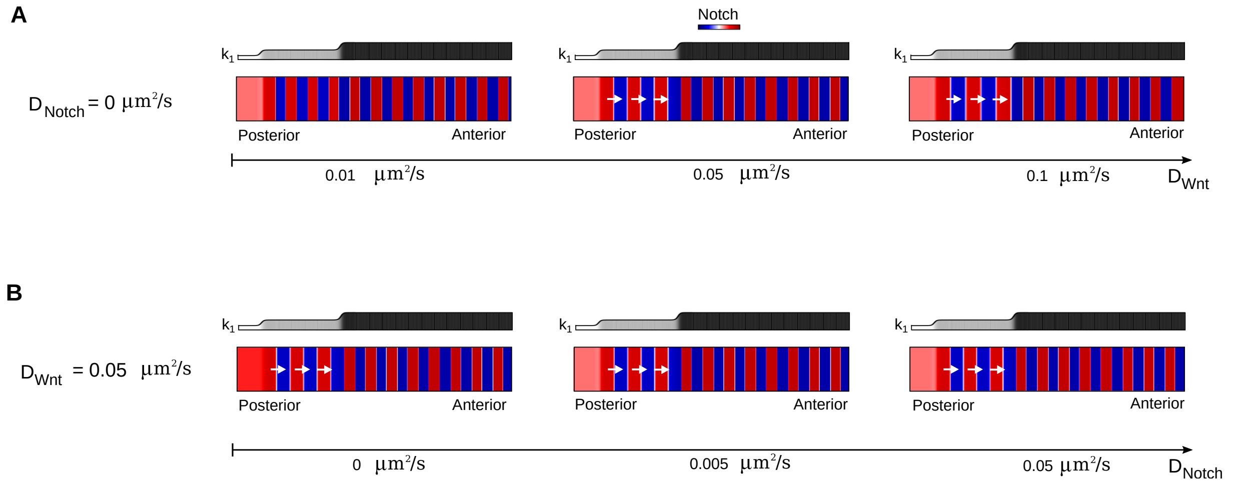

Fig. S22: **Including diffusion of Notch to the CWS model does not affect the overall dynamics**. Interpreting u as Wnt and v as Notch in the CWS model, the diffusion constant of Wnt is estimated in Section S22 to be $\mathtt{D_{Wnt}} = 0.048\ \mu\text{m}^2/\text{sec}$.
A) The CWS model is robust to increasing the diffusion of Wnt, however $\mathtt{D_{Wnt}} \leq 0.01\ \mu\text{m}$ is too low to excite the cells in the middle region to create phase waves, as shown by the lack of white arrows, while somites are still formed.
B) Including diffusion of both reactants does not change the phase waves and somite patterning of the CWS model. All parameters are shown in Tables S3 and S4 in supplementary Section S23.

Development • Supplementary information

S16 Reversed negative feedback between Wnt and Notch in the CWS

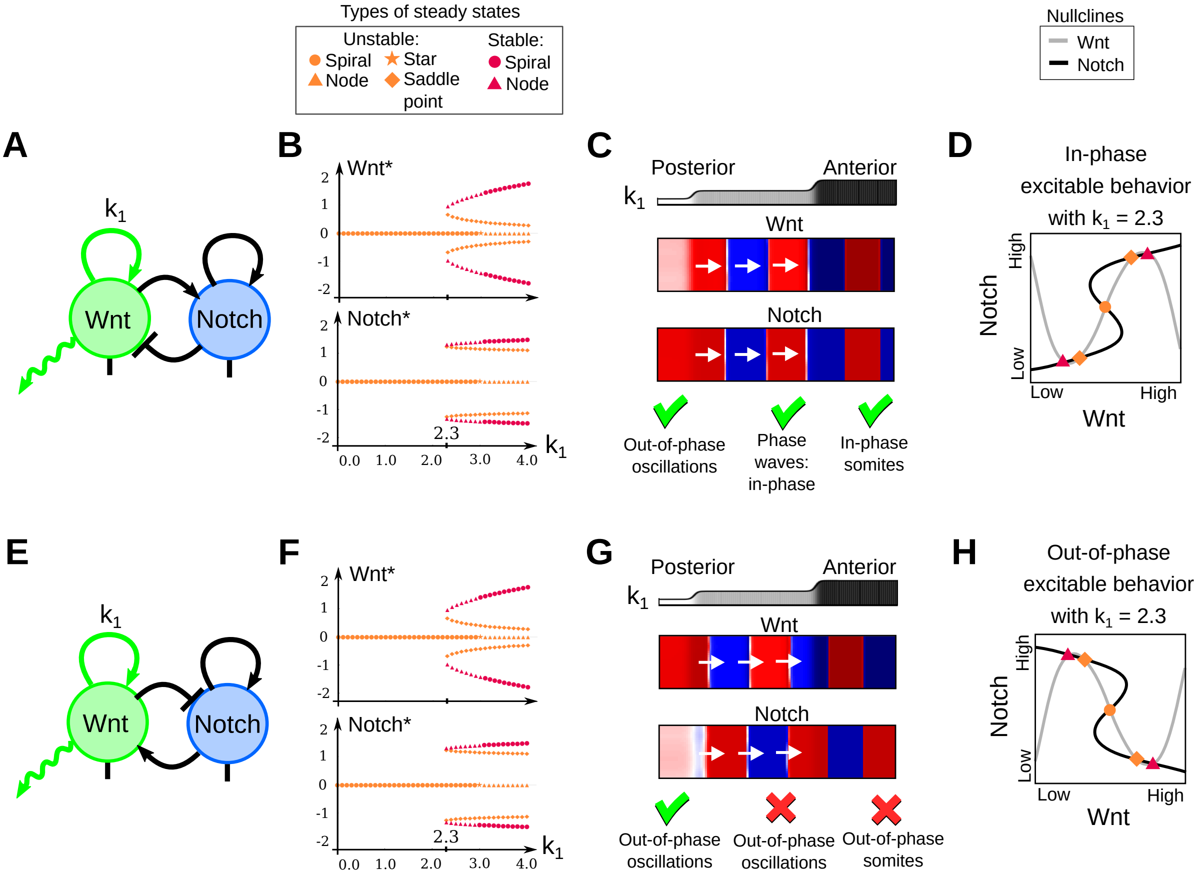

Fig. S23: **Alternative implementation of the CWS model when the signs of the negative feedback loop between Wnt and Notch are inverted.**
A,E) Network diagrams with a standard (A) or reversed (E) feedback between Wnt (u) and Notch (v).
B,F) The bifurcation diagrams of Wnt and Notch associated with the parameter k_1.
C,G) Somitogenesis simulations of the CWS model with a representation of the modulation of k_1 in the model (top line) and Wnt and Notch signaling patterns (second and third lines). Check marks show whether the different implementations of the model can reproduce the Wnt and Notch relative phases observed at the posterior, middle and anterior part of the tail in experiments (Aulehla et al., 2003; Sonnen et al., 2018).
D,H) Phase spaces for the excitable behavior with $k_1 = 2.3$.
A-D) The standard implementation of the model presented in Fig. 4 with a positive interaction from Wnt to Notch and an inhibition from Notch to Wnt. The CWS model can generate realistic patterns with the correct relative phase between Notch and Wnt at the posterior, middle and anterior part of the tail (three green marks), in agreement with experimental data (Aulehla et al., 2003; Sonnen et al., 2018).
E-H) When the incoherent negative feedback between Notch and Wnt is inverted, the CWS model cannot reproduce the relative phase change between Notch and Wnt observed in experiments. The excitable phase waves are out-of-phase when the negative feedback is inverted because the inversion causes a left-right flip of the phase space as shown in panel H, where Notch is high when Wnt is low and vice versa. All parameters are shown in Tables S3 and S4 in supplementary Section S23.

Development • Supplementary information

S17 Anterior-posterior phase shift of Wnt and Notch oscillations

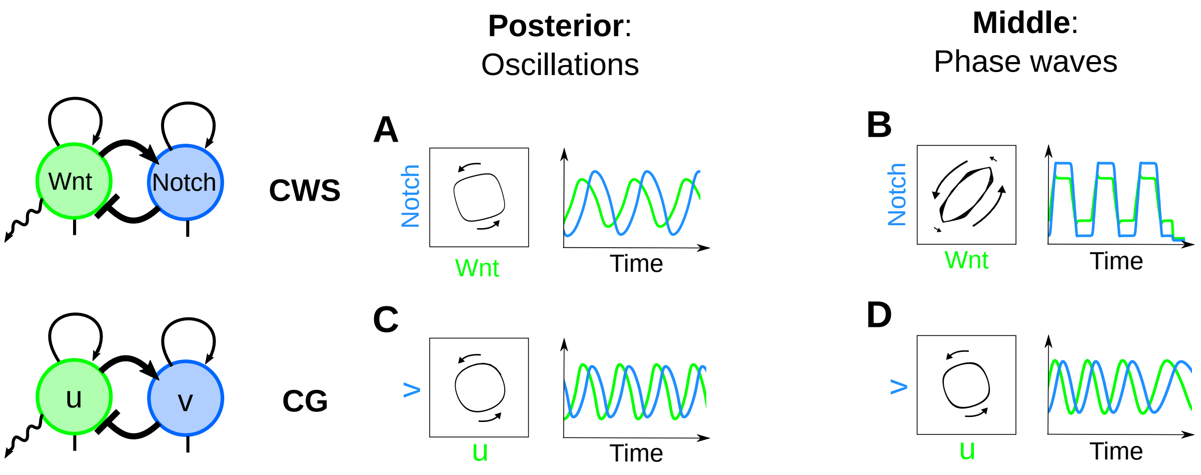

Fig. S24: **Description of the dynamics of phase change of Wnt and Notch in the Clock and Wavefront Self-Organizing (CWS) model.**
A-B) The change in phase of Wnt and Notch in the CWS from having a phase-shift in the oscillatory regime to being in-phase in the excitable regime comes from the shapes of the limit cycles in phase space for $k_1 = 1$ (A) and $k_1 = 2.3$ (B).
A) As cells oscillate around this tilted square, there are times where Wnt is high and Notch is low (upper left corner) and where Wnt is low and Notch is high (lower right corner), which explains why Wnt and Notch oscillate with a phase shift.
B) The phase waves in the excitable part of the tail of the CWS model show in-phase oscillations of Wnt and Notch. The in-phase oscillation is driven by the limit cycle that emerges from the excitation of two bistale states, where both Wnt and Notch are high (upper right corner) or low (lower left corner). The resulting limit cycle has a narrow shape that lies along the diagonal of the phase space, where Wnt and Notch are in phase. This limit cycle exhibits a narrower shape along the diagonal than the two cell simulation shown in Fig. 3D on the right, because in the tail cells experience the cumulative effect of diffusion by a larger number of cells. As a result the cells are pushed more rapidly towards the opposite steady state. Additionally, the permanence time of cells around the stable states is higher, further contributing to in-phase oscillations.
C-D) The oscillations along the anterior-posterior axis in the Sevilletor implementation of the Clock and Gradient (CG) model changes frequency due to a modulation of the parameters k_3 and k_4. Studying the oscillations in the most posterior cells, and in the middle part of the tail reveal that the limit cycle in the phase spaces are very similar and the oscillations in both places have a phase shift.

Development • Supplementary information

S18 Perturbing period of oscillations and tail growth in the CWS

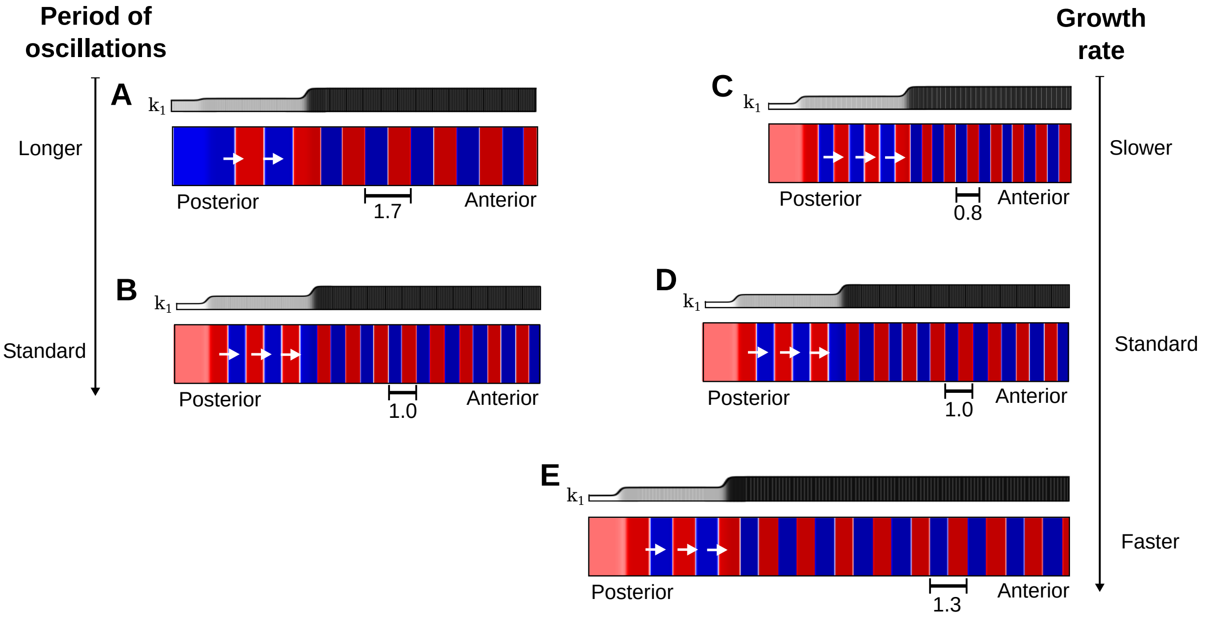

Fig. S25: **Perturbations affect the size of somites in the CWS in agreement with experiments.**
A-B) Longer period of oscillations creates larger somites (Herrgen et al., 2010). The period of oscillations is perturbed in (A) by increasing the posterior level of k_1 from 1 to 2:
A) $\Delta k_{1,\text{posterior}} = 0.3$. **B)** $\Delta k_{1,\text{posterior}} = 1.3$.
C-E) Slower progression, implemented as slower growth of the tail, leads to compressed somites (Goudevenou et al., 2011). Perturbation of the speed of progression of the growth of the tail is implemented by varying the time interval between posterior cell divisions:
C) 250. **D)** 150. **E)** 150. All parameters are shown in Tables S3 and S4 in supplementary Section S23.

Development • Supplementary information

S19 Hexagonal cells

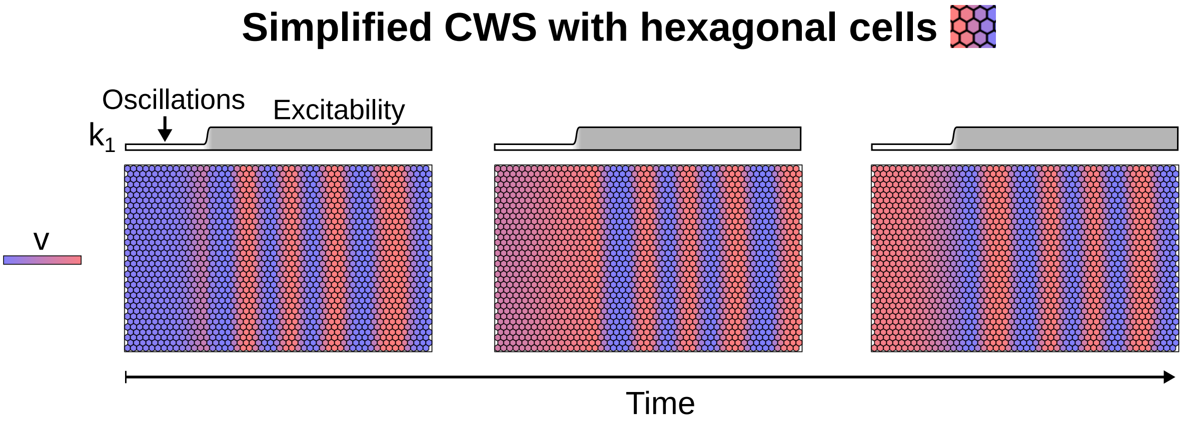

Fig. S26: **The posterior part of the tail with the CWS model on a grid with hexagonal cells shown without growth**. The simulation illustrates that waves still propagate on a straight line from the oscillatory posterior part of the tail through the excitable region of the CWS in a simplified model without growth and without a bistable anterior r egion. The rest of the parameters are as in the standard CWS model shown in Table S3 in supplementary Section S23, with $k_1 = 1$ in the posterior (white) and $k_1 = 2.3$ in the middle section (gray). The simulation was run in the software Morpheus (Starruß et al., 2014) using an hexagonal grid with $L_x = 50$ and $L_y = 35$ and node length=1 (`dx`=1).

Development • Supplementary information

S20 Detailed description of explant simulations and experiments

Ex-vivo cultures of mouse presomitic mesoderm (PSM) offer v aluable i nsights t o e xplore the coordination of oscillations during somitogenesis. An explant is formed by dissecting a portion of the embryonic tail, creating a circular quasi-monolayer culture in-vitro (Tsiairis and Aulehla, 2016) (Fig.5F). In this section, we present a detailed description of the virtual explant simulations in Fig. 5 that mirror experimental conditions.

Explants have been generated using alternative protocols that dissected either the middle (Lauschke et al., 2013) or the posterior part of the tail (Hubaud et al., 2017). In both cases, sequential signaling waves resembling somitogenesis propagated from the inner to the outer part of the explants. However, in the former case, oscillations ceased after a few cycles concomitantly with segment boundary formation (Lauschke et al., 2013), while in the second case, they persisted for up to twenty cycles over two days (Hubaud et al., 2017). The mechanisms underlying wave self-organization in explants remain unclear. In the former case, evidence suggests that cells may re-establish posterior signaling gradients (Lauschke et al., 2013), while in the latter, homogeneous distribution of signals and their targets indicates no contribution to spatial modulation (Hubaud et al., 2017). Discrepancies may arise from specific culture conditions, like removing the ectoderm and supplementing media with activators and inhibitors of BMP, FGF, WNT, RA, and ROCK, as performed in the second case.

Since the properties inherited or re-established by cells in the explants are unknown, we conducted virtual explant simulations with decreasing degrees of information (Fig.5H-M). In all simulations, cells inherit the dynamical behavior `R` that is characteristic of each model (k_1). However, they can retain the positional information provided by phase values (u and v concentration) and re-established gradients `FG` (k_3 and k_4), only the phase values, or neither. We generate explants of the PORD, CG and CWS models. To introduce a minimal degree of cell mixing similar to real explants, we assumed that cells switch positions randomly with their neighbors at $t = 0$. In addition, we vary the size of the posterior region that is projected from the tail (Fig. S30), we included explants with only the middle portion of the tail (Fig. 5I,K,M), we simulate explants where all cells are mixed together (Fig. S28), and where the center of the explant is ablated (Fig. S29). All parameters are shown in Tables S6 and S7 in supplementary Section S23.

S20.1 Wnt patterns in models of virtual explants

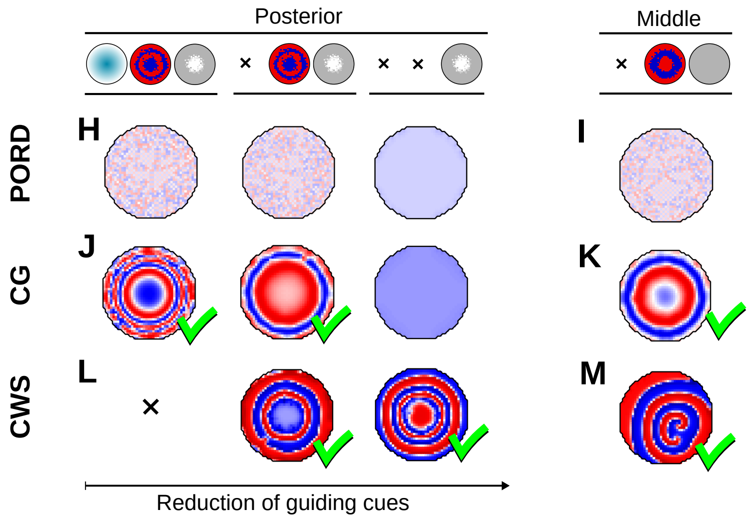

Fig. S27: **Somitogenesis explant models in Fig. 5 shown for u.** See the caption of Fig. 5.

Development • Supplementary information

S20.2 Rotating wave patterns observed in mixed explants

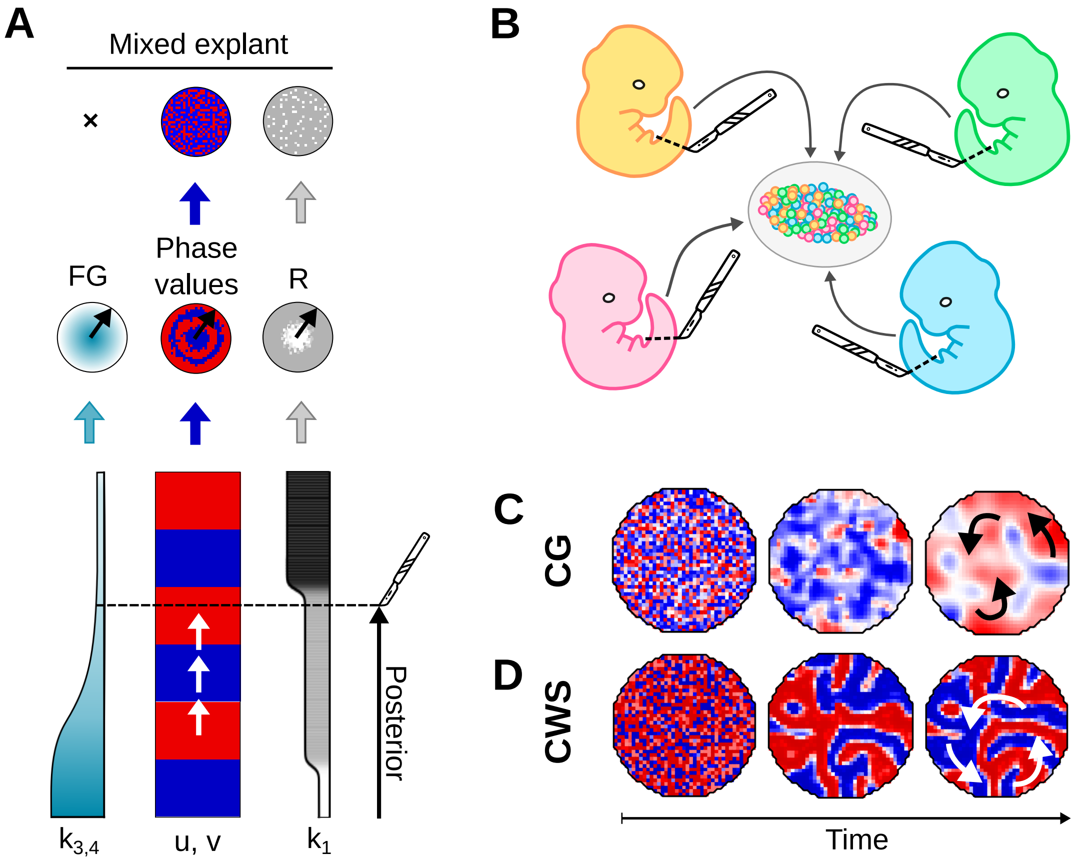

Fig. S28: **The Sevilletor implementations of the CG and CWS models recapitulate the rotating wave behavior observed in mixed explant experiments shown in Movie S3 presented in Hubaud et al. (2017)**. The corresponding simulations in the Sevilletor implementations are shown in Movie 13.
A) Virtual mixed explants of the CG and CWS model are performed by projecting a portion of the tail radially and by randomizing the positions of the cells to mimic experiments where cells are mixed from different tails. The randomized cells in the explant retain the dynamical behavior (R) and phase values (u and v) from the tail.
B) Illustration of experiments where explants are obtained by mixing cells from different tails.
C) Mixed explants in the Sevilletor implementation of the CG model shows local synchronizations between cells that promote rotating patterns (black arrows), ultimately synchronizing into homogeneous oscillations as in type IIIo systems.
D) Mixed explants of the CWS model generate periodic phase waves with a sustained rotating pattern (white arrows). All parameters are shown in Tables S6 and S7 in supplementary Section S23.

Development • Supplementary information

S20.3 Ablating the center of explants from the CWS model

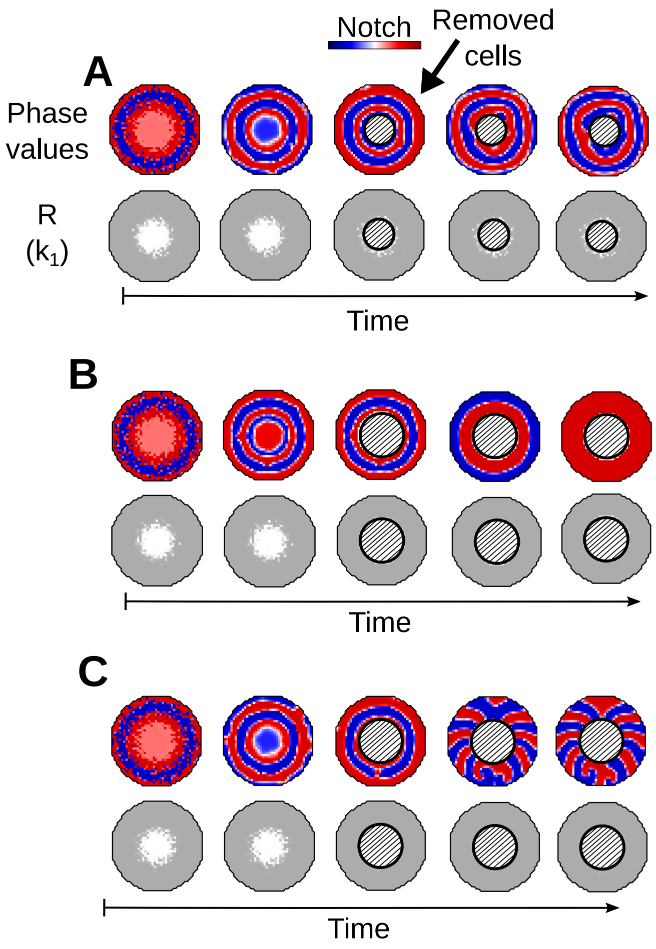

Fig. S29: **Phase waves keep propagating after ablating the center of explants derived from the Clock and Wavefront Self-Organizing model**. Time series of Notch (v) in explants made from the posterior side of the tail (Fig. 5G) of the CWS model after ablation of the center cell population where cells are in an oscillatory state with $k_1 = 1$ (white section in R).
A) When the vast majority of cells in the center is ablated (white cells in the bottom row), CWS explants keep propagating target patterns. The presence of few residual central cells (residual white cells in the bottom row) is sufficient to always sustain target patterns.
B-C) If a larger central portion is ablated in the CWS explants, the circular target patterns keep propagating initially, but depending on phase values they can either dissipate into homogeneous patterns (B) or evolve into periodic phase wave patterns that exhibit rotations (C). This is similar to the behavior observed in explants obtained solely with cells derived from the middle part of the tail (Fig. 5M). In all cases, the cells in the outer layer form phase waves in a self-organizing manner independently of global frequency gradients. All parameters are shown in Tables S6 and S7 in supplementary Section S23.

Development • Supplementary information

S20.4 Varying the position of the cut in explants

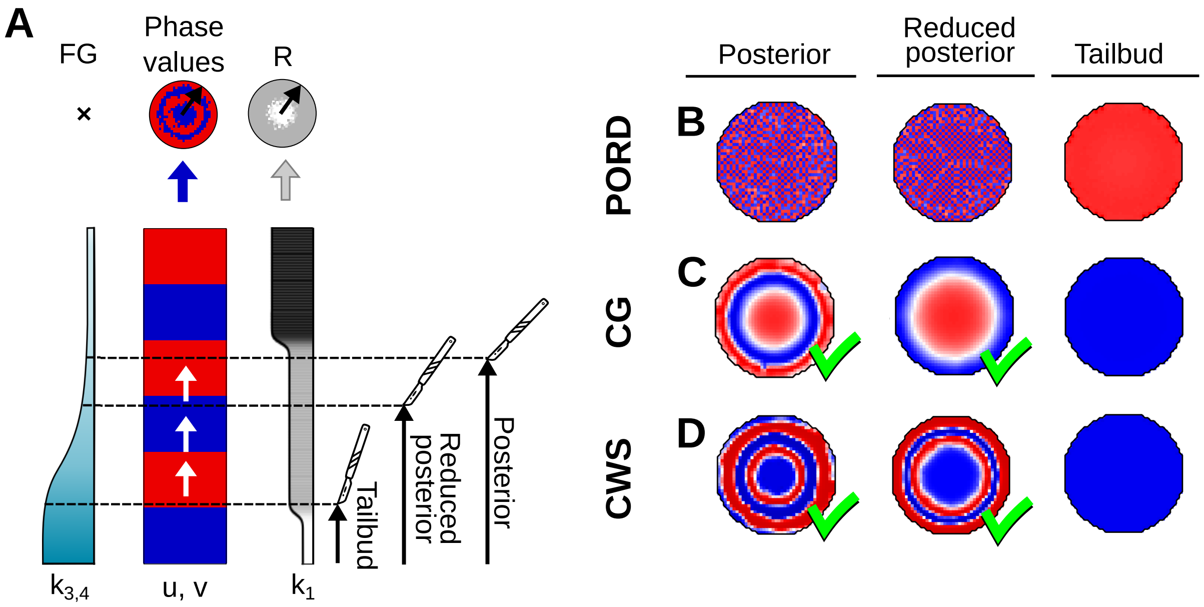

Fig.S30: **Varying the position of the cut in explants.**
A) Illustration showing three different sections of the tail used to create virtual explants in the Sevilletor implementations of the PORD, CG and CWS model. Explants retain the phase values (u and v concentrations).
B-D) The explants generate the same patterning behaviors as shown in Fig. 5H,J,L, except the case where only the posterior tailbud is cut, where all the models generate homogeneous oscillations. All parameters are shown in Tables S6 and S7 in supplementary Section S23.

Development • Supplementary information

S20.5 Possible outcomes of middle tail explants in CWS model

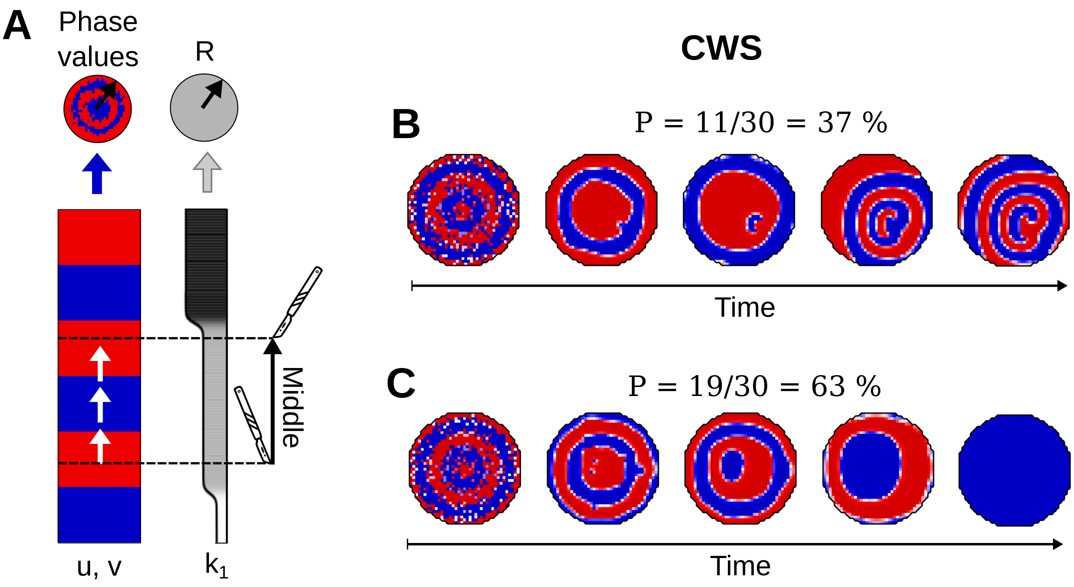

Fig. S31: **Variability in virtual middle explants of the Clock and Wavefront Self-Organizing model.**
A) Illustration of the part of the tail used to create an explant of the Clock and Wavefront Self-Organizing model that only includes cells with excitable behavior in the middle of the tail.
B-C) Summary of 30 simulations of the CWS model performed where the variability across simulations depends on the stochastic shuffling between cells performed when generating the virtual explants. Explants obtained from the middle section of the tail (without the tip) give rise to two possible outcomes: circular wave patterns with a single center made from two spirals with opposite rotation directions (Movie 12) in 37% of simulations (11/30), or a set of concentric waves that dissipates into a homogeneous pattern in 63% of the cases (19/30). This shows that when the pacemaker population from the center of the explant is not included, self-organizing target patterns can arise solely from the initial phase values inherited from the tail, but that their evolution depends on the specific initial distribution of phase values. Values are shown for v.
All parameters are shown in Tables S6 and S7 in supplementary Section S23.

Development • Supplementary information

S21 The excitable regime of the CWS model predict out of phase oscillations of neighboring cells

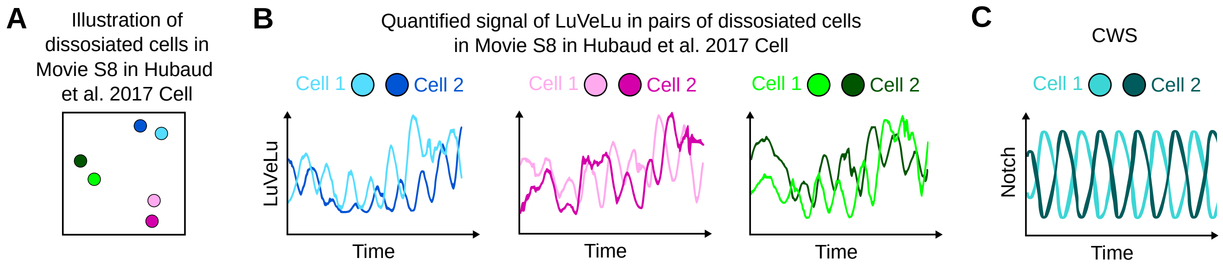

Fig. S32: **Pairs of cells with out-of-phase oscillations of LuVeLu**.
A) Illustration of dissociated cells seeded on a glass dish coated with fibronectin and treated with latrunculin A in the right panel in Movie S8 in Hubaud et al. (2017).
B) Pairs of cells in the right panel of Movie S8 from Hubaud et al. (2017) show out of phase oscillations of LuVeLu (marking Notch) in neighboring cells. The LuVeLu oscillations in individual dissociated cells is measured in Fiji (ImageJ) as the integrated density of the LuVeLu reporter for each cell over time using the Plot Z-Axis Profile function on a grayscale version of the video.
C) A simulation of a pair of neighboring cells with excitable behavior in the CWS model shows that Notch (v) oscillates out of phase between the two cells.

Development • Supplementary information

S22 Units in the CWS model

The corresponding physical units of space and time of the somitogenesis simulation in the Clock and Wavefront Self-organizing model can be estimated in the following way. The average width of a mouse somite is about 200 μm (Tam, 1981), and a mouse embryonic stem cell has a diameter of approximately 7-17 μm (Pillarisetti et al., 2009), giving the estimate that a somite is about 20 cells wide. The width of a somite in the simulation is a similar number of about 17 cells. So in the simulation the diameter of a cell is 1 space unit $\texttt{dx}$; 1 $\texttt{dx} \sim 12$ μm.

A pre-pattern of a somite is formed every 2 to 3 hours in mice (Sonnen et al., 2018; Matsuda et al., 2020), so 8 somites would be formed after about 16-24 h. The CWS can form 8 somites in 607500 time steps of duration 0.002$\texttt{dt}$ with appropriately scaled variables (factor 15) and without normalizing all reaction variables with k_2. Setting $\texttt{dt}$=1min, the estimated duration of the simulation is $\texttt{T} = 0.002\ \texttt{dt} \times 607500$ time steps $= 20.25$ h.

A verification of the estimate of the time and space relation with experimental measurements is found by calculating the corresponding diffusion constant of Wnt $\texttt{D}_{\texttt{Wnt}} = 0.02\ \texttt{dx}^2/\texttt{dt} = 0.048$ μm^2/sec, which is in the same order of magnitude as the diffusion constant measured of Wg in Drosophila, the equivalent to Wnt in mice, which is 0.05 μm^2/s (Kicheva et al., 2007).

Development • Supplementary information

S23 Parameters

Table S1. Standard parameters for the square simulations in Fig. 2E (right column) and Movie 1.

	**Lateral inhibition**	**Rotating phase waves**	**Excitable**	**Homogeneous**	**Bistable**
`dx`	1	1	1	1	1
`dt`	0.005	0.005	0.005	0.005	0.005
D (D_u)	0.3	0.3	0.3	0.3	0.3
D_v	0	0	0	0	0
L_x	100	100	100	100	100
L_y	100	100	100	100	100
(u, v) at $t = 0$	$(0, 0)$	$(0, 0)$	$(0, 0)$	$(0, 0)$	$(0, 0)$
Noise in u and v at $t = 0$	mean=0 std=0.1	mean=0 std=0.1	mean=0 std=0.1	mean=0 std=0.1	mean=0 std=0.1
k_1	0.0	1.0	2.3	3.0	4.0
k_2	1	1	1	1	1
k_3	1	1	1	1	1
k_4	1	1	1	1	1
c	0	0	0	0	0
Boundary conditions	Zero flux	Zero flux	Zero flux	Zero flux	Zero flux

Table S2. Perturbed parameters for the square simulations.

	**Variable**	**Perturbation**
Fig. 2E (left column)	Noise in u and v at $t = 0$	0
Fig. 3Q	k_1	Linear gradient in y from $0 \to 4$
	k_2	Linear gradient in x from $0 \to 4$
Fig. S1B	c	2
Fig. S6B	Boundary conditions	Periodic
Fig. S10	D	Sweep from $0.3 \to 1.0$
	k_1	Sweep from $2.3 \to 2.6$
Fig. S7B	(u, v) at $t = 0$	$(-1, -1)$
Fig. S11	D	Sweep from $0.075 \to 0.675$
	`dx`	Sweep from $0.5 \to 1.5$
	$L_x = L_y$	100×`dx`

Development • Supplementary information

Table S3. Standard parameters for the Sevilletor implementation of the somitogenesis models in Fig. 4D,E,H,K.

	**CW**	**PORD**	**CG**	**CWS**
`dx`	1	1	1	1
`dt`	0.002	0.002	0.002	0.002
D (D_u)	0	1.0	0.3	0.3
D_v	0	0	0	0
L_y	35	12	35	35
L_x at $t = 0$	20	50	20	20
u at $t = 0$	-1	0	-1	-1
v at $t = 0$	-1	-1 (except $v = 1$ in the most anterior row)	-1	-1
Posterior cell division every n'th dt	200	1700	100	200
k_1	(see `R`)	0	(see `R`)	(see `R`)
k_2	1	1	1	1
k_3	1	(see `FG`)	(see `FG`)	1
k_4	1	(see `FG`)	(see `FG`)	1
$a_{\mathtt{R}}$	1	-	1	1
$\Delta k_{1,\text{posterior}}$	-	-	-	1.3
$\Delta k_{1,\text{anterior}}$	3	-	3	1.7
a_{FG}	-	0.1	0.1	-
$\Delta k_{3,4}$	-	0.3	2	-
Summary for `R`: Posterior to anterior (k_1)	k_1: $1 \to 4$ at $x = 80$	-	k_1: $1 \to 4$ at $x = 80$	k_1: $1 \to 2.3 \to 4$ at $x = 20$ and 80
Summary for `FG`: Posterior to anterior (k_3 and k_4)	-	$k_{3,4}$: $1.3 \to 1$ with middle value at $x = 25$	$k_{3,4}$: $3 \to 1$ with middle value at $x = 50$	-
Boundary conditions	Zero flux	Zero flux	Zero flux	Zero flux

Development • Supplementary information

Table S4. Perturbed parameters for somitogenesis simulations.

	**Model(s)**	**Variable**	**Perturbation**
Fig. 4Q	CWS	R	Linear increase from $k_1 = 1 \rightarrow 4$
Fig. 4G	PORD	Multiplicative noise u, v	$\delta = 5\%$
		Multiplicative noise k_3 and k_4	$\delta = 0.5\%$
Fig. 4J	CG	Multiplicative noise u, v	$\delta = 5\%$
		Multiplicative noise k_1	$\delta = 0.5\%$
		Multiplicative noise k_3 and k_4	$\delta = 0.5\%$
Fig. 4M and Fig. S16B	CWS	Multiplicative noise u, v	$\delta = 5\%$
		Multiplicative noise k_1	$\delta = 0.5\%$
		Multiplicative noise k_3 and k_4	$\delta = 0.5\%$
Fig. S16C	CWS	Multiplicative noise in u and v	$\delta \in [1\%, 10\%]$
		Multiplicative noise in k_1	$\delta \in [0.1\%, 1.0\%]$
		Multiplicative noise in k_3 and k_4	$\delta \in [0.1\%, 1.0\%]$
Fig. S17	CG and CWS	P_{swap} per cell per dt	0.0005
Fig. S20A	CWS	Boundary conditions	Zero flux boundary introduced between $k_1 = 1$ and 2.3
Fig. S20B	CWS	Boundary conditions	Zero flux boundary introduced between $k_1 = 1$ and 2.3
		Posterior cell division every n'th dt	Never
Fig. S22A	CWS	D_u (D_{Wnt})	$[0.0625, 0.3, 0.625]$ ($[0.10, 0.05, 0.01]$ μm^2/sec)
Fig. S22B	CWS	D_v (D_{Notch})	$[0, 0.03, 0.3]$ ($[0.000, 0.005, 0.050]$ μm^2/sec)
Fig. S23B	CWS	k_3, k_4	-1
Fig. S21D	CG	R	k_1: $1 \rightarrow 4$ at $x = 150$
		FG	$k_{3,4}$: $3 \rightarrow 1$ with middle value at $x = 130$
		a_{FG}	0.25
Fig. S21F	CWS	R	k_1: $1 \rightarrow 2.3 \rightarrow 4$ at $x = 40$ and 160
Fig. S21G	CG	FG	$k_{3,4}$: $3 \rightarrow 1$ with middle value at $x = 110$
Fig. S21H	CG	R	k_1: $1 \rightarrow 4$ at $x = 150$
Fig. S21I	CG	R	k_1: $1 \rightarrow 4$ at $x = 150$
		FG	$k_{3,4}$: $3 \rightarrow 1$ linear
Fig. S21J	CG	R	k_1: $1 \rightarrow 4$ at $x = 150$
		FG	$k_{3,4} = 1$ (flat)
Fig. S18	CWS	L_y	$[5, 35, 100]$
Fig. S19	CWS	R	k_1: $1 \rightarrow 2.3$ at $x = [5, 25, 80]$
			k_1: $2.3 \rightarrow 4$ at $x = [80, 85, 150]$
Fig. S12A-C	PORD	dx	$[0.5, 1.0, 1.5]$
		D (D_u)	[0.3, 1.0, 2.1]
Fig. S12D-I	CG and CWS	dx	$[0.5, 1.0, 1.5]$
		D (D_u)	$[0.08, 0.30, 0.63]$
Fig. S25A	CWS	$\Delta k_{1,\text{posterior}}$	0.3
Fig. S25C-E	CWS	Posterior cell division every n'th dt	$[150, 200, 250]$

Development • Supplementary information

Table S5. Parameters for excitability simulations in Fig. 5A-E.

	**Fig. 5A-B)** Close proximity	**Fig. 5A-B)** Separated	**Fig. 5C-E)** Oscillating	**Fig. 5C-E)** Notch inhibition
`dx`	1 (12 μm)	5 (60 μm)	1	1
`dt`	0.002	0.002	0.002	0.002
D (D_u)	0.3/15	0.3/15	0.3	0.3
D_v	0	0	0	0
(u, v) for cell 1 at $t = 0$	$(0.5, 0)$	$(0.5, 0)$	$(0.5, 0)$	Continuing from oscillations
(u, v) for cell 2 at $t = 0$	$(-0.5, 0)$	$(-0.5, 0)$	$(-0.5, 0)$	Continuing from oscillations
k_1	2.3/15	2.3/15	2.3	2.3
k_2	1/15	1/15	1	0
k_3	1/15	1/15	1	1
k_4	1/15	1/15	1	1

Table S6. Standard parameters for the Sevilletor implementation of the somitogenesis models in explants.

	**PORD**	**CG**	**CWS**
`dx`	1	1	1
`dt`	0.002	0.002	0.002
D (D_u)	1.0	0.3	0.3
D_v	0	0	0
Radius	20	20	20
`R` (k_1)	From the tail: $k_1 = 0$	From the tail: $k_1 = 1$	From the tail: $k_1 = 1 \rightarrow 2.3$
k_2	1	1	1
Boundary conditions	Zero flux	Zero flux	Zero flux
Shuffling of cells at $t = 0$	2 rounds of switching positions with P=0.45 for each cell	2 rounds of switching positions with P=0.45 for each cell	2 rounds of switching positions with P=0.45 for each cell

Development • Supplementary information

Table S7. Perturbed parameters for somitogenesis simulations of explants.

	**Model(s)**	**Variable**	**Perturbation**
Fig. 5H	PORD	u and v at $t = 0$<br>FG ($k_{3,4}$)<br>Section of the tail	(Left and middle) From the tail. (Right) $v = u = 0$.<br>(Left) From the tail. (Middle and right) $k_{3,4} = 1.3$.<br>Whole posterior: $x = 1 \to 30$
Fig. 5J	CG	u and v at $t = 0$<br>FG ($k_{3,4}$)<br>Section of the tail	(Left and middle) From the tail. (Right) $v = u = 0$.<br>(Left) From the tail. (Middle and right) $k_{3,4} = 3$.<br>Whole posterior: $x = 1 \to 70$
Fig. 5L	CWS	u and v at $t = 0$<br>FG ($k_{3,4}$)<br>Section of the tail	(Middle) From the tail. (Right) $v = u = 0$.<br>$k_{3,4} = 1$<br>Whole posterior: $x = 1 \to 50$
Fig. 5I,K,M and Fig. S31B-C	PORD, CG and CWS	R (k_1)<br>u and v at $t = 0$<br>FG ($k_{3,4}$)<br>Section of the tail	From the tail: $k_1 = 0$ (PORD), 1 (CG), 2.3 (CWS)<br>From the tail<br>$k_{3,4} = 1.3$ (PORD), 3 (CG), 1 (CWS)<br>Middle:<br>PORD: $x = 11 \to 30$<br>CG: $x = 20 \to 70$<br>CWS: $x = 26 \to 70$
Fig. S28C	CG without diffusion	D (D_u)<br>Shuffling of cells at $t = 0$<br>u and v at $t = 0$<br>FG ($k_{3,4}$)<br>Section of the tail	0<br>Completely randomized cell positions<br>From the tail (randomized)<br>$k_{3,4} = 3$<br>Whole posterior: $x = 1 \to 70$
Fig. S28D-E	CG and CWS	Shuffling of cells at $t = 0$<br>u and v at $t = 0$<br>FG ($k_{3,4}$)<br>Section of the tail	Completely randomized cell positions<br>From the tail (randomized)<br>$k_{3,4} = 3$ (CG), 1 (CWS)<br>Whole posterior: $x = 1 \to 70$ (CG), 50 (CWS)
Fig. S29	CWS	Radius of ablation center<br>$T_{ablation}$<br>u and v at $t = 0$<br>Section of the tail	A) 7.0 B) 10.5 C) 10.0<br>A) 5500 B) 6300 C) 5500<br>From the tail<br>Whole posterior: $x = 1 \to 50$
Fig. S30B-D (first row)	PORD, CG and CWS	u and v at $t = 0$<br>FG ($k_{3,4}$)<br>Section of the tail	From the tail<br>$k_{3,4} = 1.3$ (PORD), 3 (CG), 1 (CWS)<br>Whole posterior:<br>$x = 1 \to 30$ (PORD), 70 (CG), 50 (CWS)
Fig. S30B-D (second row)	PORD, CG and CWS	u and v at $t = 0$<br>FG ($k_{3,4}$)<br>Section of the tail	From the tail<br>$k_{3,4} = 1.3$ (PORD), 3 (CG), 1 (CWS)<br>Reduced posterior:<br>$x = 1 \to 25$ (PORD), 60 (CWS), 30 (CWS)
Fig. S30B-D (third row)	PORD, CG and CWS	u and v at $t = 0$<br>FG ($k_{3,4}$)<br>Section of the tail	From the tail<br>$k_{3,4} = 1.3$ (PORD), 3 (CG), 1 (CWS)<br>Posterior tailbud: $x = 1 \to 10$

S24 Captions of Movies

Movies of two-dimensional square simulations of the Sevilletor model, two-cell simulations in phase space and somitogenesis simulations of the tail and tail explants for the Sevilletor implementations of the PORD, CG and CWS model. The number of time steps in each movie varies to illustrate the dynamics of the patterning processes. All parameters are shown in Tables S1-S7 in supplementary Section S23.

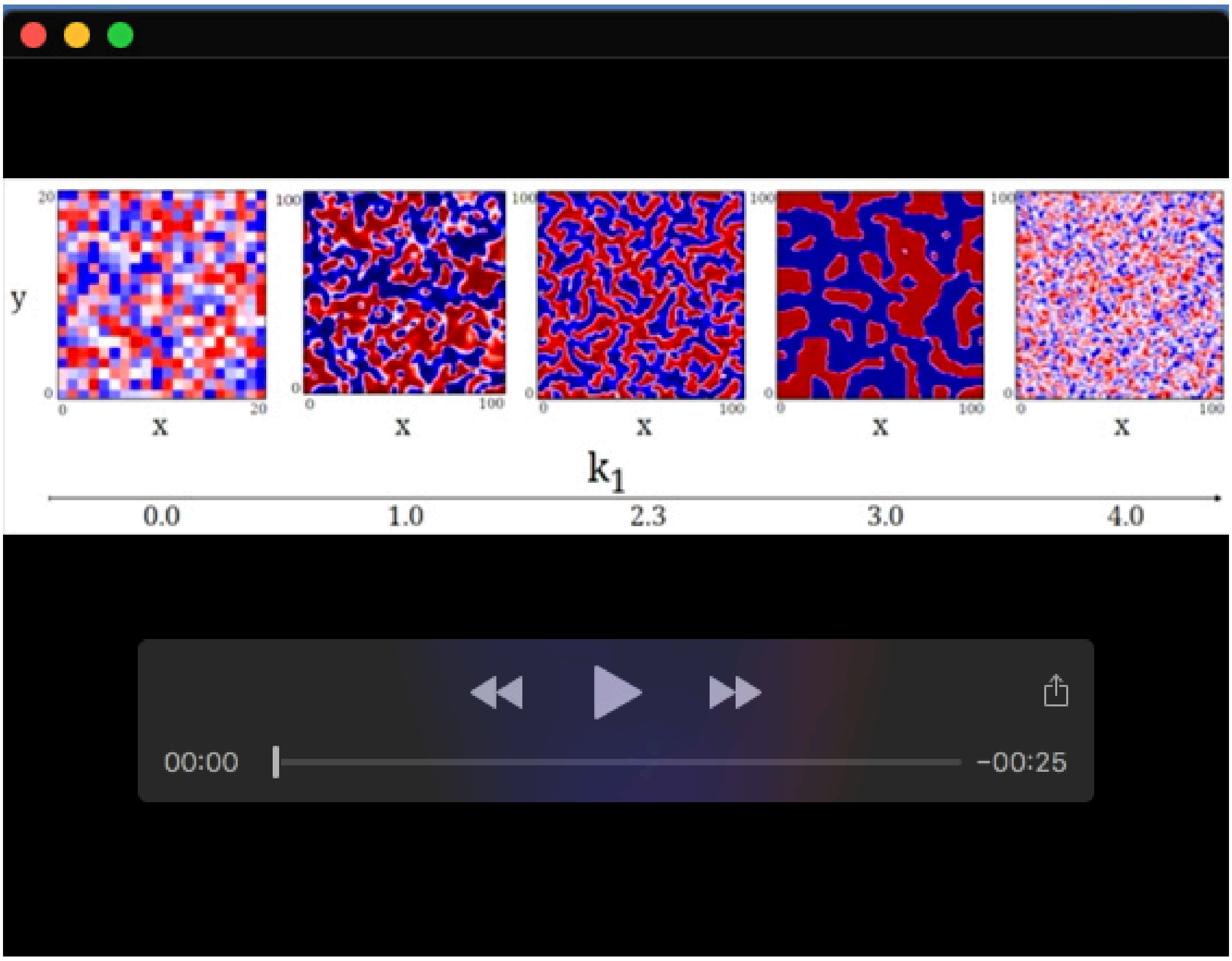

Movie 1. Self-organizing patterning behaviors of the Sevilletor model
From left to right, two dimensional simulations of the Sevilletor model started with noisy initial concentrations for increasing values of k_1, as shown in Fig. 2E. With k_1 = 0.0 the model forms a lateral inhibition chessboard pattern, with k_1 = 1.0 rotating waves, with k_1 = 2.3 excitable periodic wave patterns with spiral centers, with k_1 = 3.0 a homogeneous pattern and with k_1 = 4.0 a bi-stable salt and pepper pattern.

Development • Supplementary information

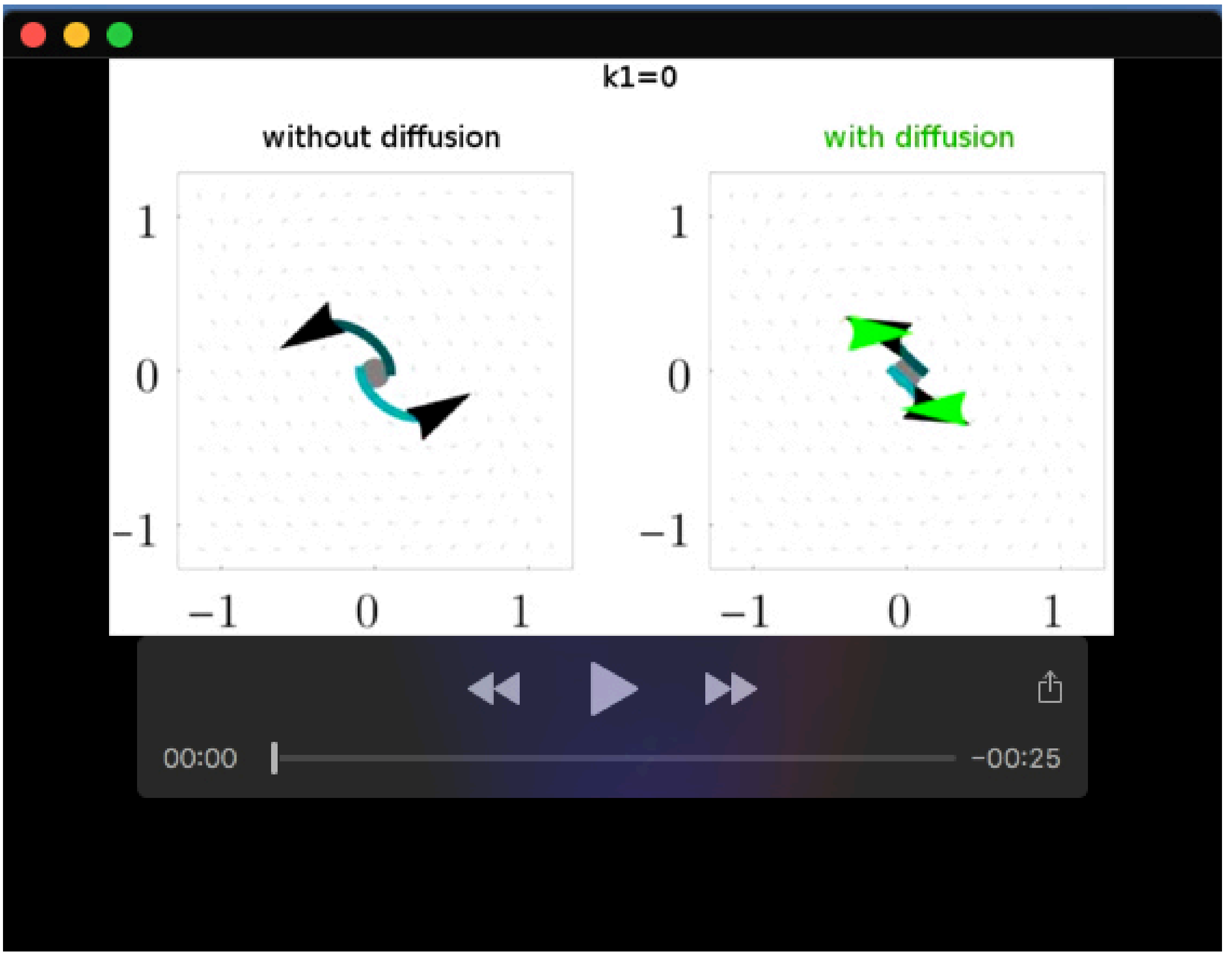

Movie 2. Phase space trajectories of two cells in lateral inhibition patterning

Two-cell simulations in phase space for the lateral inhibition regime with $k_1 = 0.0$ in the absence or presence of diffusion, as showed in Fig. 3B. The trajectories of the two cells with initial values $(u, v) = (\pm 0.1, 0.0)$ are shown in teal colored lines, the gray point shows the central unstable steady state. Without diffusion (graph on the left) the two cells oscillate by following a limit cycle around the central steady state. In the presence of diffusion (graph on the right) the two cells stop at opposite phase where the changes promoted by reaction (black arrows) are balanced by the changes promoted by diffusion (green arrows). This behavior generates chessboard patterns as shown in Fig. 2E.

Development • Supplementary information

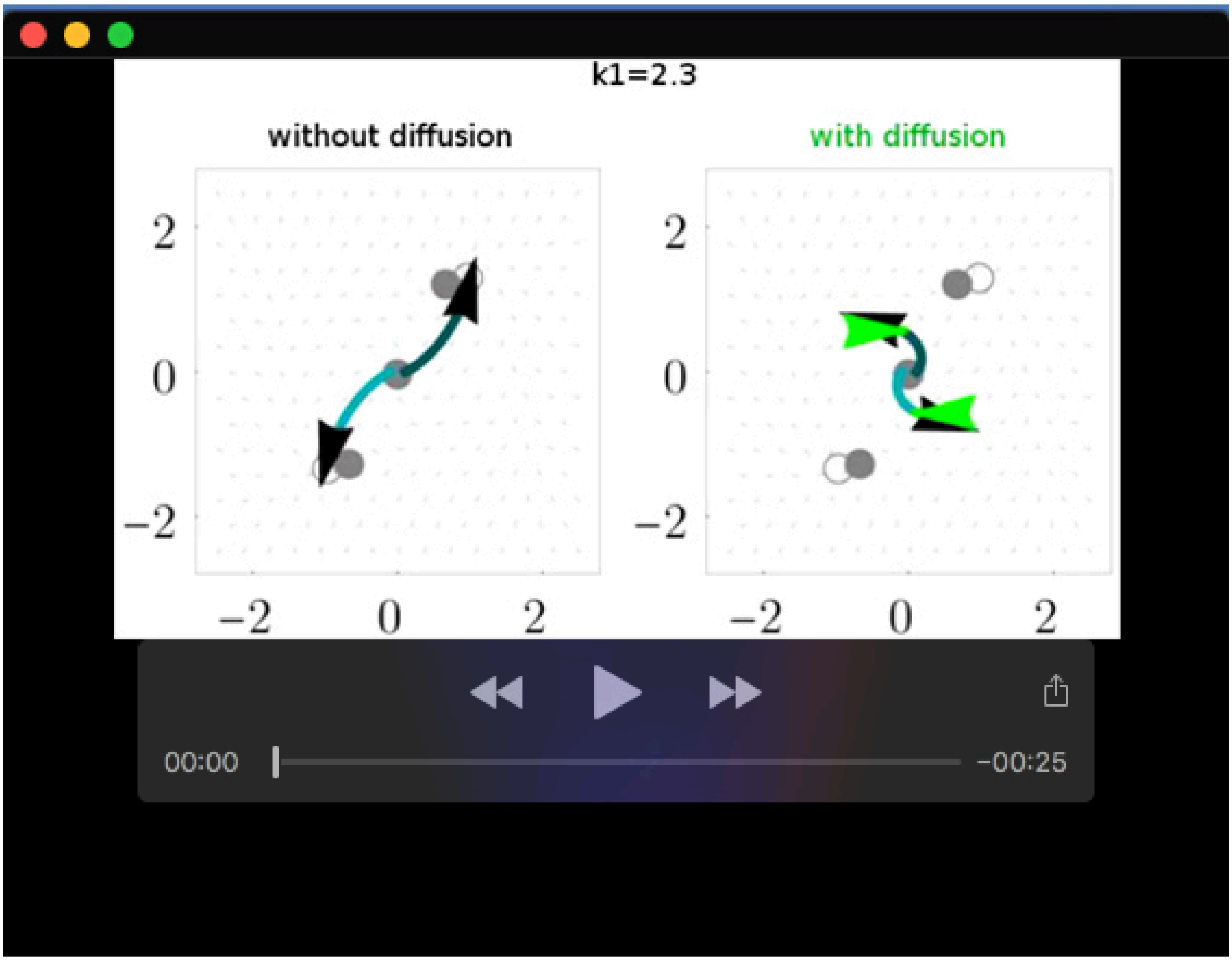

Movie 3. Phase space trajectories of two cells with excitable periodic phase wave patterning behavior
Two-cell simulations in phase space for the periodic wave regime with $k_1 = 2.3$ in the absence or presence of diffusion, as showed in Fig. 3D. The trajectories of the two cells with initial values $(u, v) = (\pm 0.1, 0.0)$ are shown in teal colored lines, the gray points are unstable steady states and white points are stable steady states. Without diffusion (graph on the left) each cell is pushed by reaction (black arrows) into one of the stable steady states. In the presence of diffusion (graph on the right), the influence of reaction (black arrows) is counterbalanced by diffusion (green arrows) which pushes each cell onto the trajectory of the other unstable gray point creating coordinated loops. This behavior generates periodic wave patterns with spiral centers from noise as shown in Fig. 2E.

Development • Supplementary information

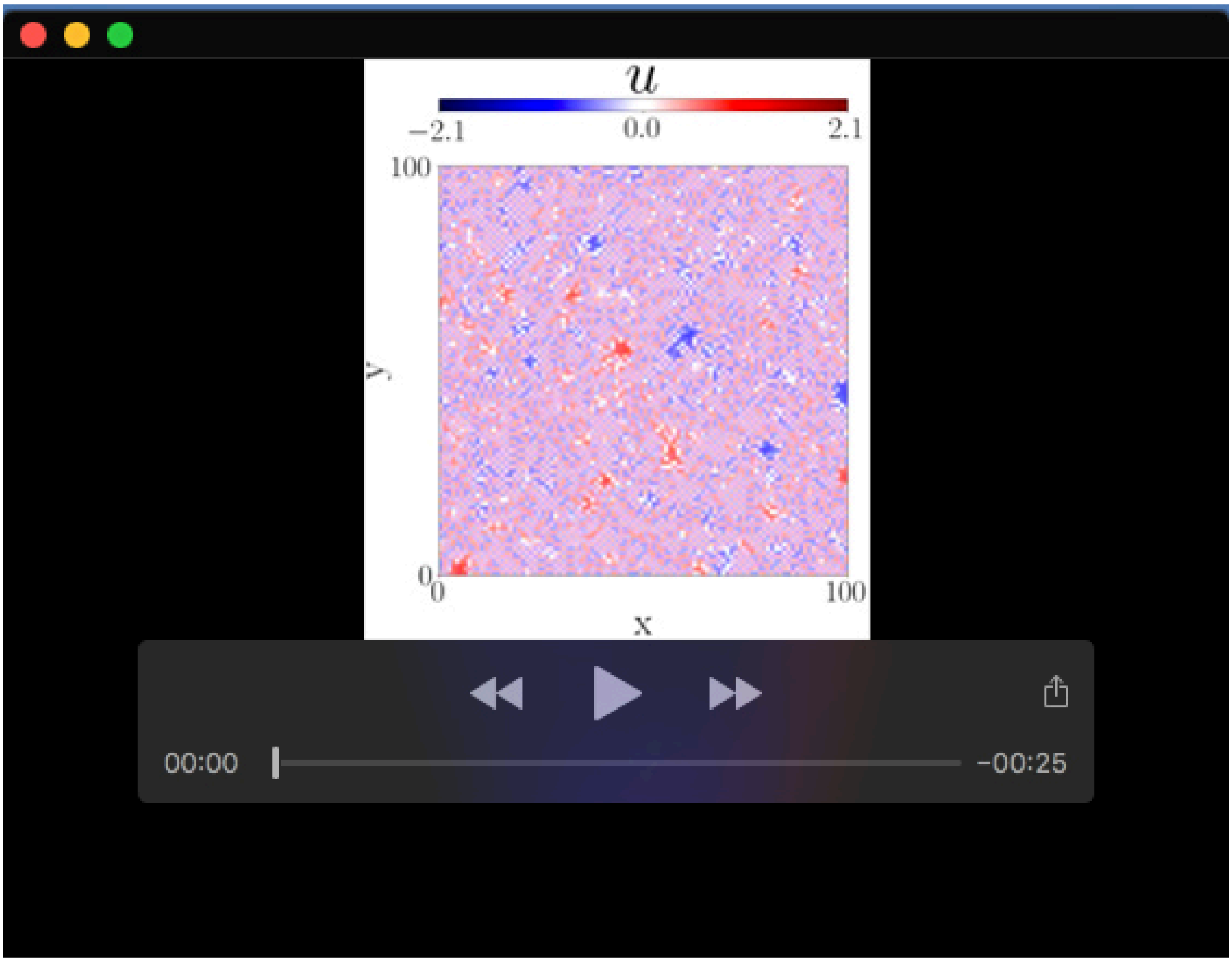

Movie 4. Changing the patterning dynamics by varying the strength of k_1

The behavior of the Sevilletor model can be easily modulated to transition between patterning regimes over time by varying the parameter k_1 in all cells. In this example, the model transitions from lateral inhibition pattern, to rotating waves, to periodic waves with spiral centers, to a bi-stable frozen spiral pattern, and back to a lateral inhibition pattern: $k_1 = 0.0 \rightarrow 1.0 \rightarrow 2.3 \rightarrow 4.0 \rightarrow 0.0$.

Development • Supplementary information

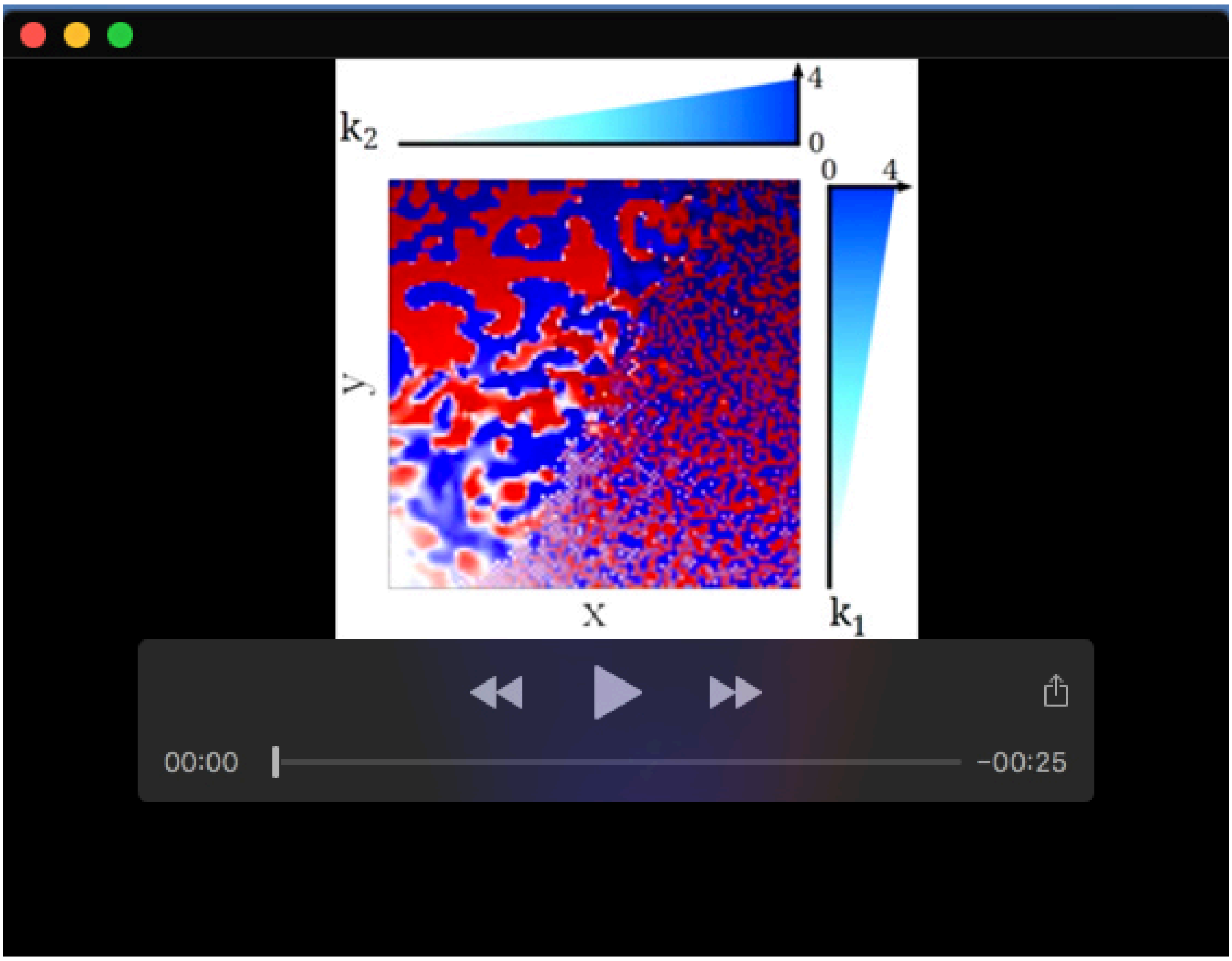

Movie 5. k_1 and k_2 determines the self-organizing patterning

Square two-dimensional simulation with noisy initial conditions and linear modulations of the parameters k_1 and k_2 along the y- and x-axis, as shown in Fig. 3H. The two parameters increase linearly from 0 to 4 promoting transitions between lateral inhibition pattern, rotating waves, periodic wave patterns, homogeneous patterns and bi-stable frozen patterns.

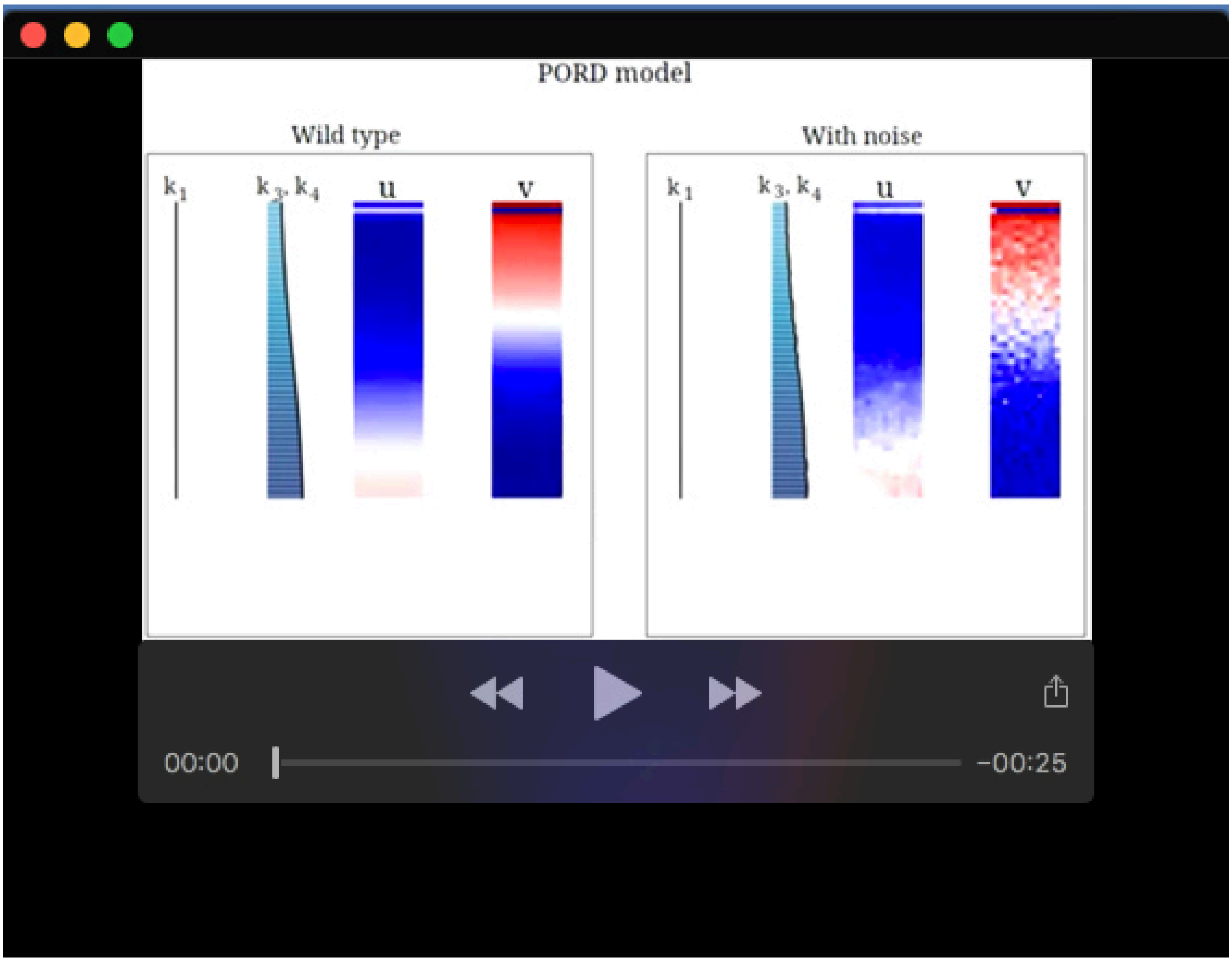

Movie 6. Sevilletor implementation of the PORD model
2D simulation of the PORD model implemented in the Sevilletor framework (Fig. 4E). Left: wild type simulation. Modulations promoted by R (k_1) and FG (k_3, k_4), and u and v signaling are shown side-by-side. All cells have $k_1 = 0.0$ and the simulation starts with a pre-patterned somite (high signaling at the top boundary). The virtual tail grows with a constant speed by addition of a line of cells at the bottom (posterior). A gradient of k_3 and k_4 changes the frequency along the x-axis to create phase waves. The model generates a periodic somite pattern by freezing cells due to a relay mechanism based on lateral inhibition. Right: Simulation with 5% multiplicative noise added to phase values (u, v) and 0.5% multiplicative noise to parameters (k_3, k_4). The PORD model is fragile to noise due to its tendency to generate lateral inhibition patterns.

Development • Supplementary information

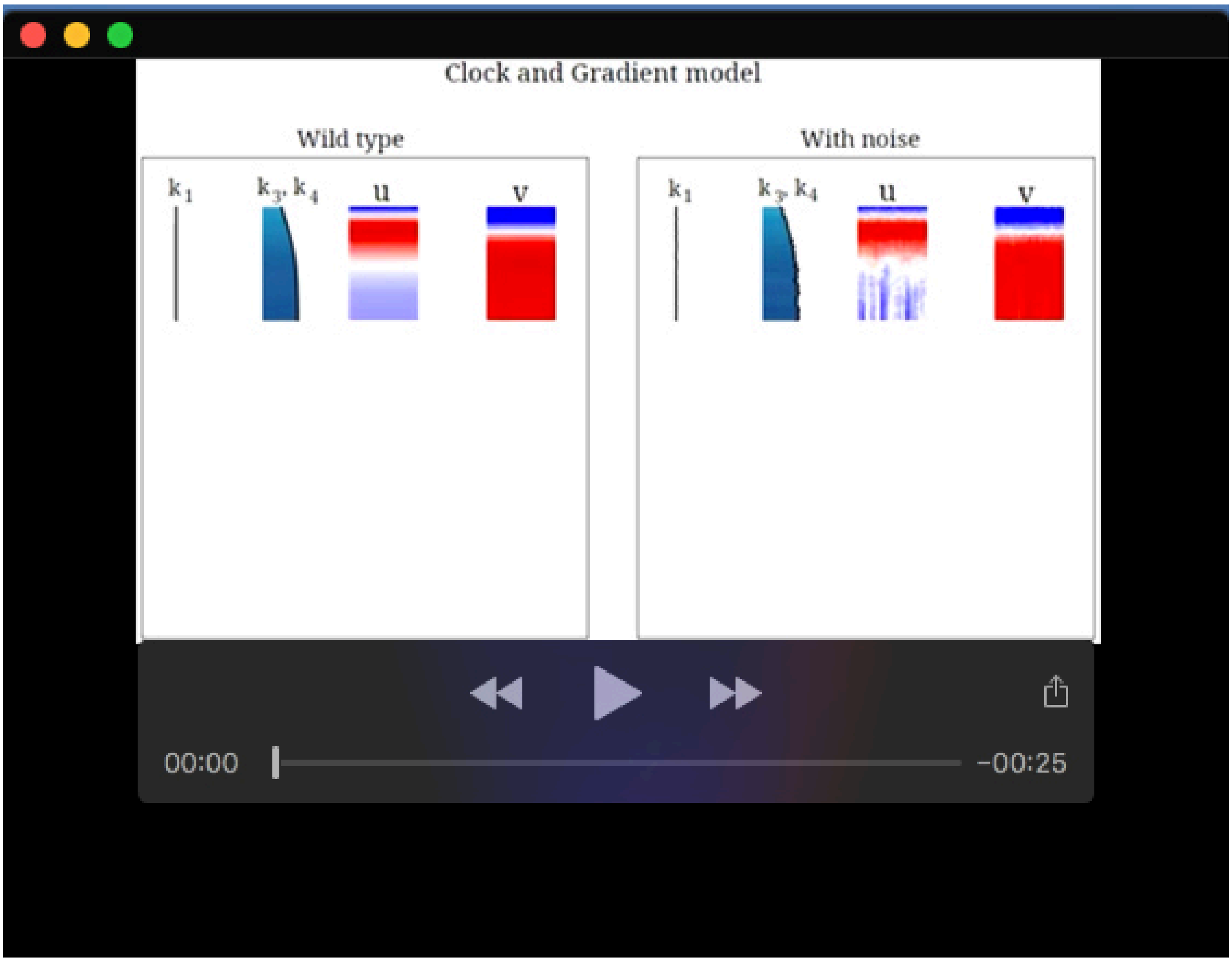

Movie 7. Sevilletor implementation of the Clock and Gradient (CG) model
2D simulation of the Clock and Gradient model implemented in the Sevilletor framework (Fig. 4H). Left: wild type simulation. Modulations promoted by R ($k1$) and FG ($k3, k4$), and u and v signaling are shown side-by-side. The virtual tail grows with a constant speed by addition of a line of cells at the bottom (posterior). The posterior side (bottom) has $k1 = 1$ that promotes out of phase oscillations of the two reactants. The anterior side (top) has a bistable behavior that promotes a progressive freezing of oscillations, which generates a periodic somite pattern with $k1 = 4$. Phase waves are promoted by a gradient of $k3$ and $k4$ that controls the frequency of oscillations. Right: Simulation with 5% multiplicative noise added to phase values (u, v) and 0.5% multiplicative noise to parameters ($k1, k3, k4$). The model is robust to noise but as time progresses, the noise that accumulates in the frequency gradient begins to disorganize the somite pattern.

Development • Supplementary information

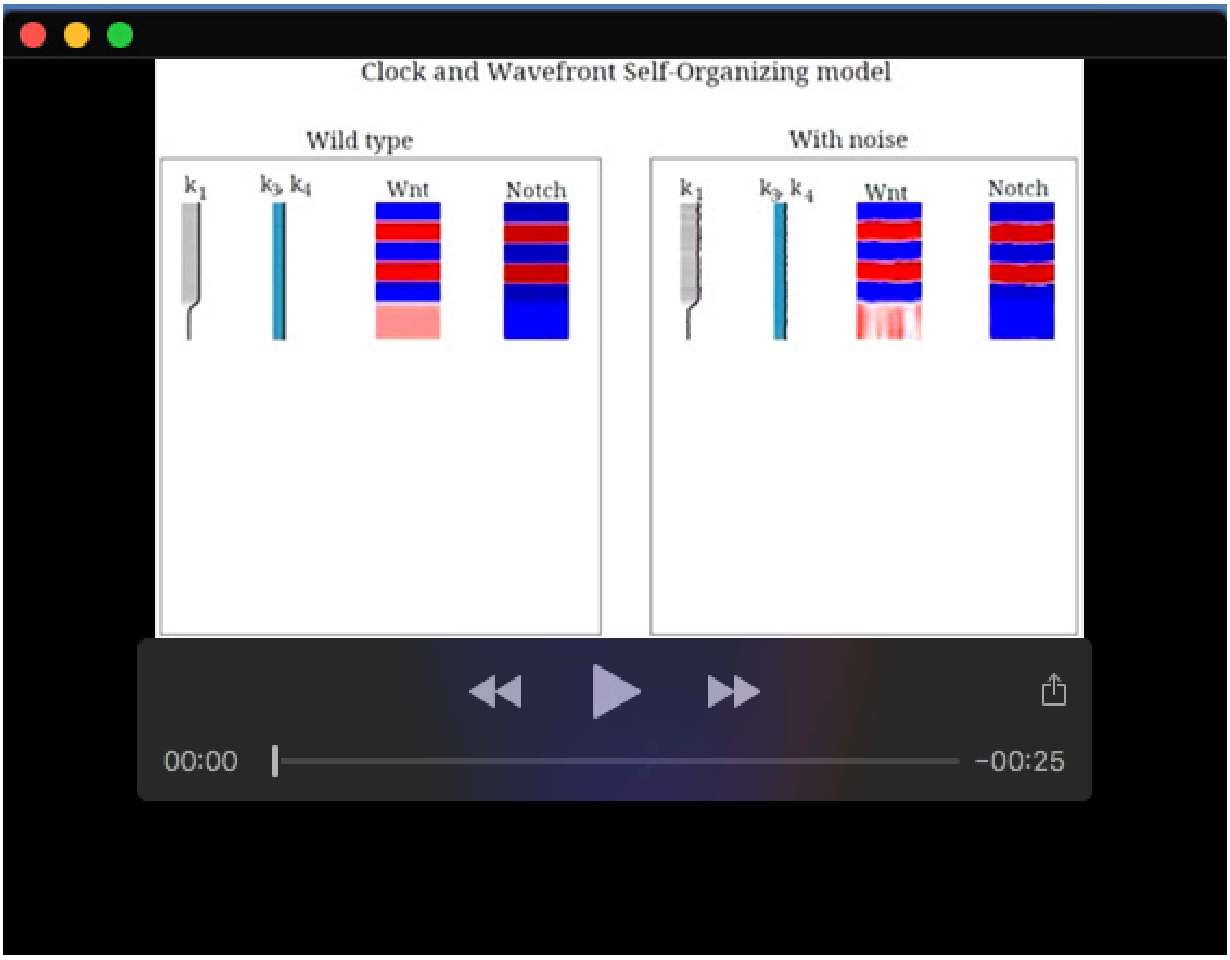

Movie 8. The Clock and Wavefront Self-Organizing (CWS) model recapitulates somitogenesis patterning behavior

Somitogenesis simulations of the novel Clock and Wavefront Self-Organizing model implemented within the Sevilletor framework (Fig. 4K). Left: wild type simulation. Modulations promoted by R (k_1), Wnt (u) and Notch (v) signaling are shown side-by-side. The virtual tail grows with a constant speed by addition of a line of cells at the bottom (posterior). The posterior side (bottom) has $k_1 = 1$ that promotes out of phase oscillations of Wnt and Notch. The anterior side (top) is in a bistable behavior that forms somites with $k_1 = 4$. In the middle part of the tail, intermediate values of $k_1 = 2.3$ promote in-phase oscillations of Wnt and Notch that propagate in the form of waves due to an excitable behavior, showed in the simulation by an overlapping region of high Wnt and Notch that moves anteriorly before freezing. Right: Simulation with 5% multiplicative noise added to phase values (u, v) and 0.5% multiplicative noise to parameters (k_1, k_3, k_4). The excitable behavior and the independence from a frequency gradient makes the model robust to noise.

Development • Supplementary information

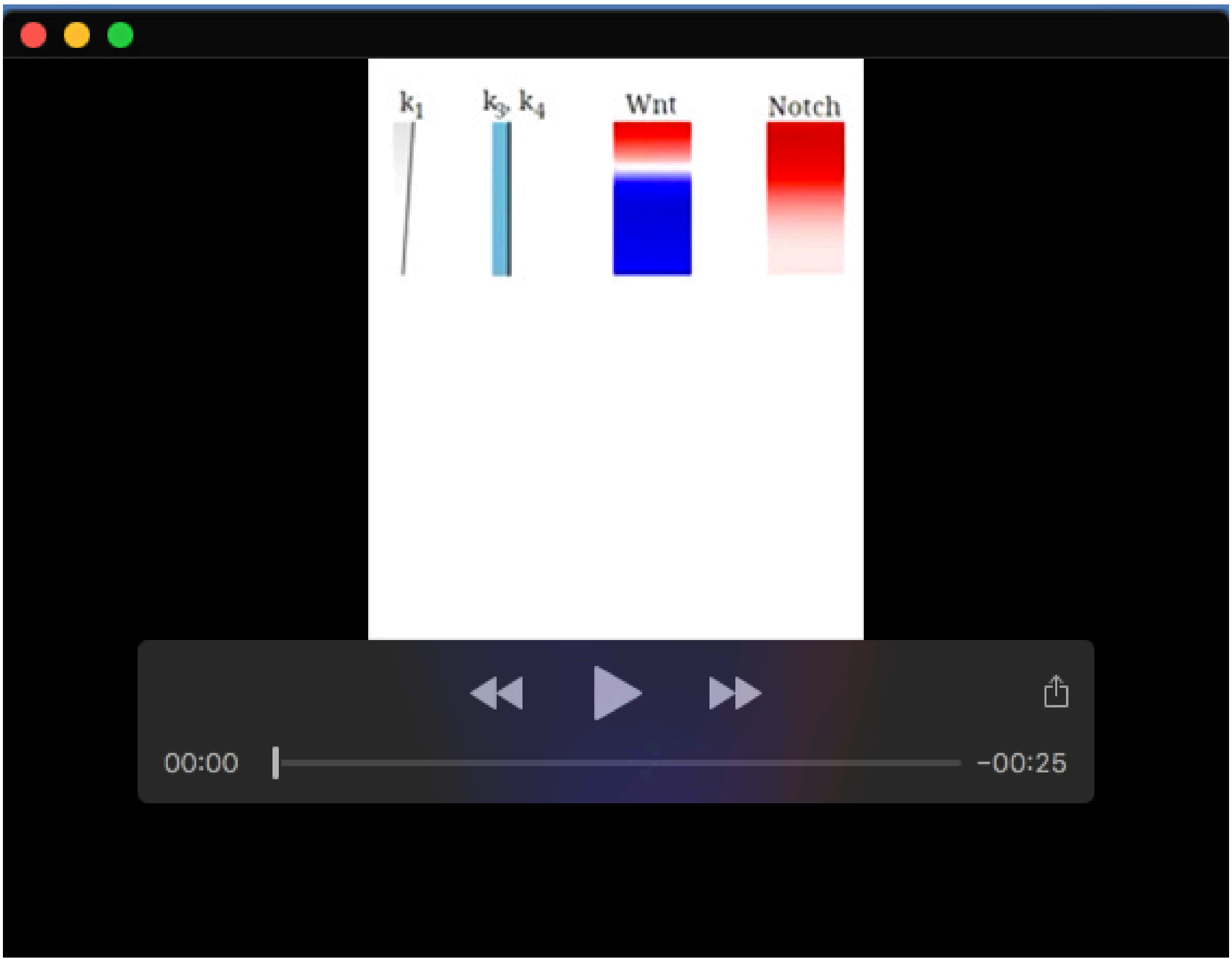

Movie 9. The Clock and Wavefront Self-Organizing model can capture the changes in phase wave width observed *in-vivo*

The CWS model can be adjusted to incorporate a linear graded modulation of R (k_1) instead of a step-wise change to capture the changes in phase wave width observed in-vivo (Fig. 4Q). Wnt waves slow down as they move anteriorly, while Notch waves propagate at the same speed catching up to the Wnt waves and ending up in phase.

Development • Supplementary information

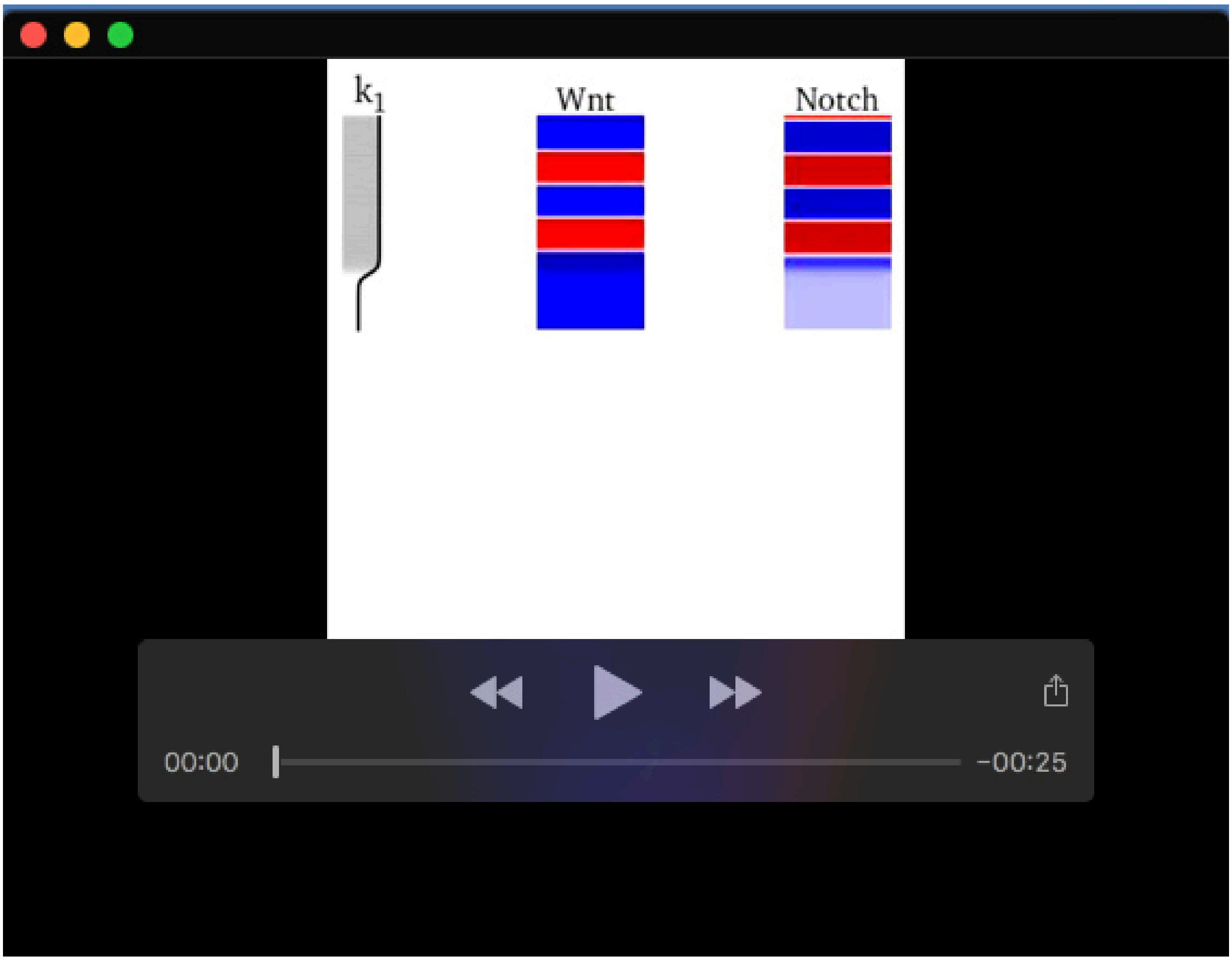

Movie 10. Virtual dissection of the tailbud in the CWS model

Movie showing the effect of tailbud removal in a simulation using the Clock and Wavefront Self-Organizing model (Fig. S20A). Following the separation of the posterior region, the tailbud continues to grow and to generate new phase waves that travel anteriorly. In the anterior dissected part, preexisting phase waves persist and propagate anteriorly, driven by the excitable behavior in the intermediate region (light gray section), until they stop when entering the bistable region (dark gray section). These findings align with experimental observations reported in Özelçi et al. (2022). Importantly, in the anterior dissected part, the propagation of straight waves without rotational movements occurs independently of the homogeneous oscillations at the posterior tailbud tip.

Development • Supplementary information

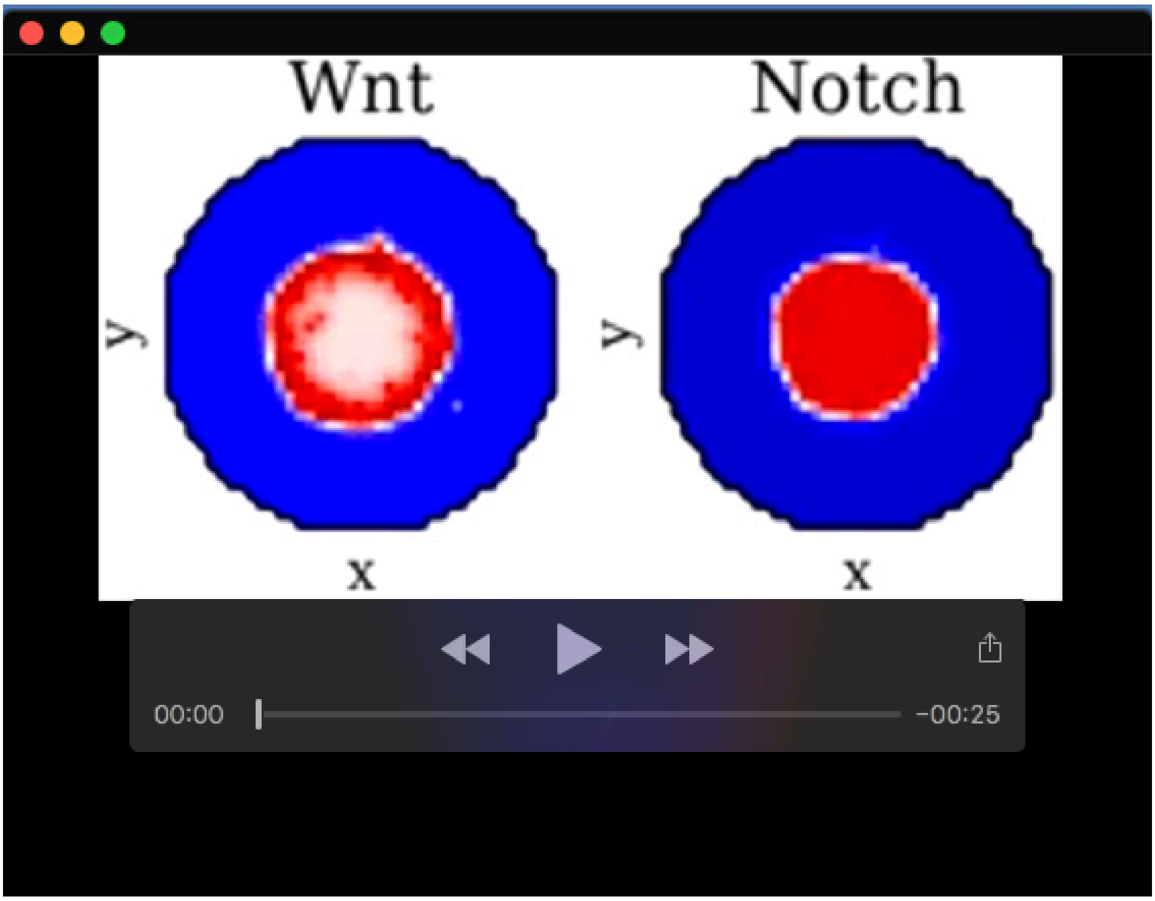

Movie 11. Explant of the Clock and Wavefront Self-Organizing model creates circular waves without a phase value pre-pattern

Simulated explant obtained from the whole posterior part of the tail in the Clock and Wavefront Self-Organizing model (Fig. 5L to the right). Simulated Wnt (u) and Notch (v) signaling are shown side-by-side. The virtual explant is generated by making a radial projection along the anterior-posterior axis for the whole posterior part of the tail, with cells in the center in an oscillatory regime (k_1 = 1.0) and outermost cells in an excitable regime (k_1 = 2.3). The cells initially have low values of u and v. In agreement with experiment (Hubaud et al., 2017), oscillations at the center of the explant propagate towards the periphery.

Development • Supplementary information

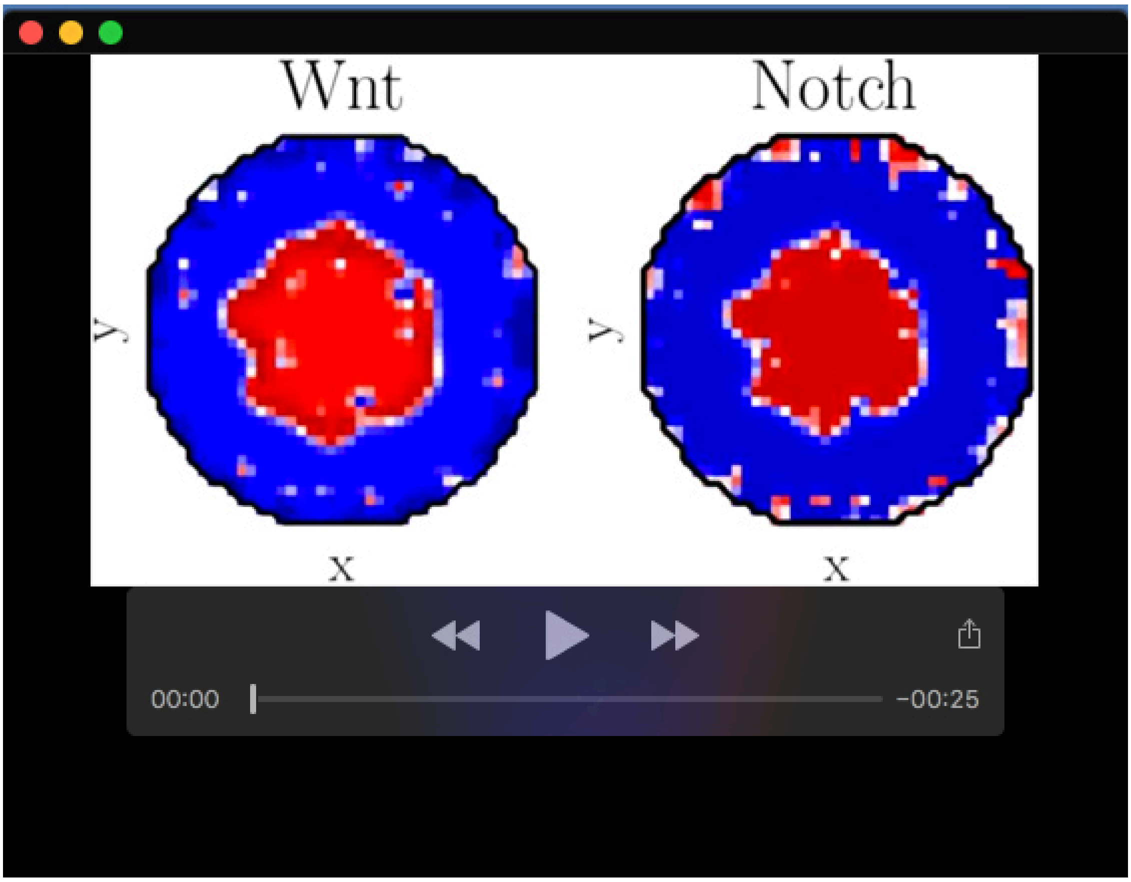

Movie 12. Explant of the Clock and Wavefront Self-Organizing model creates circular waves with excitable behavior

Simulated explant obtained from the middle part of the tail in the Clock and Wavefront Self-Organizing model (Fig. 5M). The simulation recapitulates the circular Notch signaling waves that propagate from the center of the colony towards the periphery observed in experiments (Lauschke et al., 2013). Simulated Wnt (u) and Notch (v) signaling are shown side-by-side. The virtual explant is generated by making a radial projection of average values along the anterior-posterior axis for the middle part of the tail with cells in an excitable regime (k_1 = 2.3) that generate circular wave patterns due to two spiral centers that rotate in opposite directions. This type of pattern depends on initial conditions and arise in 37 % (11/30) of simulations (Fig. S31).

Development • Supplementary information

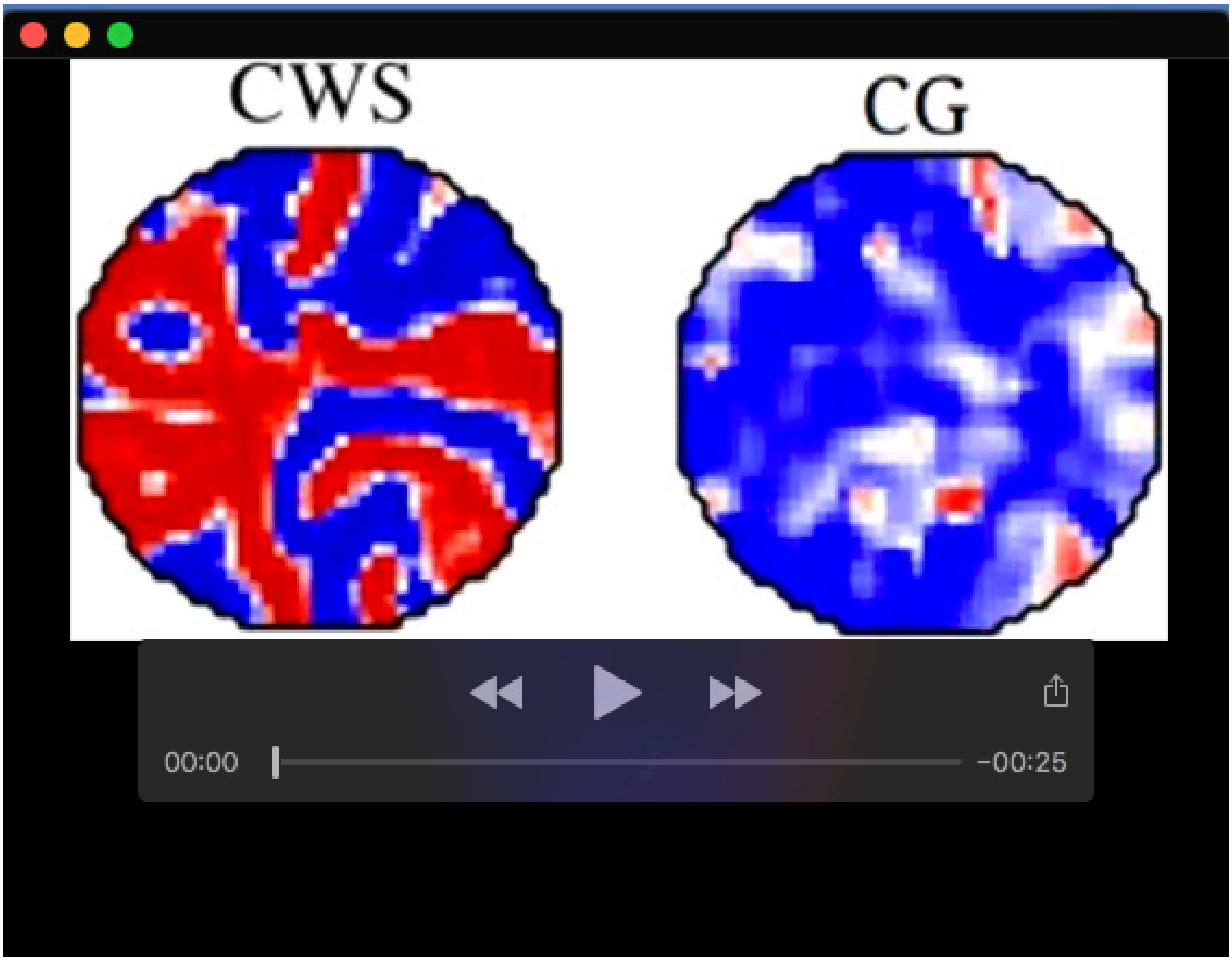

Movie 13. Waves in mixed cell explants

Mixed explants from the Sevilletor implementations of the CWS and CG model where u/Wnt and v/Notch signaling are shown side-by-side (Fig. S28). Mixed explants of the Clock and Wavefront Self-Organizing (CWS) model generate periodic phase waves with a sustained rotating pattern due to the excitable behavior of the cells and local synchronization. The Clock and Gradient (CG) model allows local synchronizations of oscillatory behavior between cells to promote rotating patterns.

References

Aranson, I. S. and Kramer, L. (2002). The world of the complex ginzburg-landau equation. *Reviews of Modern Physics* **74**.

Aulehla, A., Wehrle, C., Brand-Saberi, B., Kemler, R., Gossler, A., Kanzler, B. and Herrmann, B. G. (2003). Wnt3a plays a major role in the segmentation clock controlling somitogenesis. *Developmental Cell* **4**, 395–406.

Aulehla, A., Wiegraebe, W., Baubet, V., Wahl, M. B., Deng, C., Taketo, M., Lewandoski, M. and Pourquié, O. (2008). A beta-catenin gradient links the clock and wavefront systems in mouse embryo segmentation. *Nature Cell Biology* **10**, 186–193.

Ay, A., Knierer, S., Sperlea, A., Holland, J. and Özbudak, E. M. (2013). Short-lived her proteins drive robust synchronized oscillations in the zebrafish segmentation clock. *Development* **140**, 3244–3253.

Breakspear, M., Heitmann, S. and Daffertshofer, A. (2010). Generative models of cortical oscillations: Neurobiological implications of the kuramoto model. *Frontiers in Human Neuroscience* **4**.

Cotterell, J., Robert-Moreno, A. and Sharpe, J. (2015). A local, self-organizing reaction-diffusion model can explain somite patterning in embryos. *Cell Systems* **1**, 257–269.

Cross, M. C. and Hohenberg, P. C. (1993). Pattern formation outside of equilibrium. *Reviews of Modern Physics* **65**, 851.

Field, R. J. and Noyes, R. M. (1974). Oscillations in chemical systems. iv. limit cycle behavior in a model of a real chemical reaction. *The Journal of Chemical Physics* **60**, 1877–1884.

FitzHugh, R. (1961). Impulses and physiological states in theoretical models of nerve membrane. *Biophysical Journal* **1**, 445–466.

François, P., Hakim, V. and Siggia, E. D. (2007). Deriving structure from evolution: Metazoan segmentation. *Molecular Systems Biology* **3**, 154.

Goudevenou, K., Martin, P., Yeh, Y. J., Jones, P. and Sablitzky, F. (2011). Def6 is required for convergent extension movements during zebrafish gastrulation downstream of wnt5b signaling. *PLoS ONE* **6**.

Herrgen, L., Ares, S., Morelli, L. G., Schröter, C., Jülicher, F. and Oates, A. C. (2010). Intercellular coupling regulates the period of the segmentation clock. *Current Biology* **20**, 1244–1253.

Hubaud, A., Regev, I., Mahadevan, L. and Pourquié, O. (2017). Excitable dynamics and yap-dependent mechanical cues drive the segmentation clock. *Cell* **171**, 668–682.e11.

Jho, E.-H., Zhang, T., Domon, C., Joo, C.-K. and Freund, C. (2002). Wnt/beta-catenin/tcf signaling induces the transcription of axin2, a negative regulator of the signaling pathway. *Molecular and Cellular Biology* **22**, 1172–1183.

Jutras-Dubé, L., El-Sherif, E. and François, P. (2020). Geometric models for robust encoding of dynamical information into embryonic patterns. *eLife* **9**, e55778.

Jörg, D. J., Oates, A. C. and Jülicher, F. (2016). Sequential pattern formation governed by signaling gradients. *Physical Biology* **13**.

Kicheva, A., Pantazis, P., Bollenbach, T., Kalaidzidis, Y., Bitting, T., Jülicher, T. and González-Gaitán, M. (2007). Kinetics of morphogen gradient formation. *Science* **315**.

Development • Supplementary information

Kuyyamudi, C., Menon, S. N. and Sinha, S. (2022). Morphogen-regulated contact-mediated signaling between cells can drive the transitions underlying body segmentation in vertebrates. *Physical Biology* **19**, 16001.

Lauschke, V. M., Tsiairis, C. D., François, P. and Aulehla, A. (2013). Scaling of embryonic patterning based on phase-gradient encoding. *Nature* **493**, 101–105.

Lewis, J. (2003). Autoinhibition with transcriptional delay: A simple mechanism for the zebrafish somitogenesis oscillator. *Current Biology* **13**, 1398–1408.

Matsuda, M., Hayashi, H., Garcia-Ojalvo, J., Yoshioka-Kobayashi, K., Kageyama, R., Yamanaka, Y., Ikeya, M., Toguchida, J., Alev, C. and Ebisuya, M. (2020). Species-specific segmentation clock periods are due to differential biochemical reaction speeds. *Science* **369**, 1450–1455.

Mongera, A., Rowghanian, P., Gustafson, H. J., Shelton, E., Kealhofer, D. A., Carn, E. K., Serwane, F., Lucio, A. A., Giammona, J. and Campàs, O. (2018). A fluid-to-solid jamming transition underlies vertebrate body axis elongation. *Nature* **561**, 401–405.

Morelli, L. G., Ares, S., Herrgen, L., Schröter, C., Jülicher, F. and Oates, A. C. (2009). Delayed coupling theory of vertebrate segmentation. *HFSP Journal* **3**, 55–66.

Novák, B. and Tyson, J. J. (2008). Design principles of biochemical oscillators. *Nature Reviews Molecular Cell Biology* **9**, 981–991.

Pantoja-Hernández, J., Breña-Medina, V. F. and Santillán, M. (2021). Hybrid reaction-diffusion and clock-and-wavefront model for the arrest of oscillations in the somitogenesis segmentation clock. *Chaos: An Interdisciplinary Journal of Nonlinear Science* **31**, 063107.

Pillarisetti, A., Ladjal, H., Ferreira, A., Keefer, C. and Desai, J. (2009). Mechanical characterization of mouse embryonic stem cells. 2009 Annual International Conference of the IEEE Engineering in Medicine and Biology Society.

Prigogine, I. and Lefever, R. (1968). Symmetry breaking instabilities in dissipative systems. ii. *The Journal of Chemical Physics* **48**, 1695–1700.

Prigone, I. (1978). Time, structure and fluctuations. *Science* **201**, 777–785.

Roehl, H. and Nüsslein-Volhard, C. (2001). Zebrafish pea3 and erm are general targets of fgf8 signaling. *Current Biology* **11**.

Shepelev, I. A. and Vadivasova, T. E. (2019). Variety of spatio-temporal regimes in a 2d lattice of coupled bistable fitzhugh-nagumo oscillators. formation mechanisms of spiral and double-well chimeras. *Communications in Nonlinear Science and Numerical Simulation* **79**, 104925.

Sonnen, K. F., Lauschke, V. M., Uraji, J., Falk, H. J., Petersen, Y., Funk, M. C., Beaupeux, M., François, P., Merten, C. A. and Aulehla, A. (2018). Modulation of phase shift between wnt and notch signaling oscillations controls mesoderm segmentation. *Cell* **172**, 1079–1090.e12.

Starruß, J., Back, W. D., Brusch, L. and Deutsch, A. (2014). Morpheus: A user-friendly modeling environment for multiscale and multicellular systems biology. *Bioinformatics* **30**, 1331–1332.

Takashima, Y., Ohtsuka, T., González, A., Miyachi, H. and Kageyama, R. (2011). Intronic delay is essential for oscillatory expression in the segmentation clock. *PNAS* **108**, 3300–3305.

Tam, P. P. L. (1981). The control of somitogenesis in mouse embryos. *Development* **65**, 103–128.

Development • Supplementary information

Torabi, R. and Davidsen, J. (2019). Pattern formation in reaction-diffusion systems in the presence of non-markovian diffusion. *Phys. Rev. E* **100**, 052217.

Tsiairis, C. D. and Aulehla, A. (2016). Self-organization of embryonic genetic oscillators into spatiotemporal wave patterns. *Cell* **164**, 656–667. Somitogenesis.

Tyson, J. J. and Keener, J. P. (1988). Singular perturbation theory of traveling waves in excitable media (a review)*. *Physica D* **32**, 327–361.

Uriu, K., Liao, B. K., Oates, A. C. and Morelli, L. G. (2021). From local resynchronization to global pattern recovery in the zebrafish segmentation clock. *eLife* **10**.

Wang, S., Garcia-Ojalvo, J. and Elowitz, M. B. (2022). Periodic spatial patterning with a single morphogen. *Cell Systems* **13**, 1033–1047.

Zhabotinsky, A. M. and Zaikin, A. N. (1973). Autowave processes in a distributed chemical system. *Journal of Theoretical Biology* **40**, 45–61.

Özelçi, E., Mailand, E., Rüegg, M., Oates, A. C. and Sakar, M. S. (2022). Deconstructing body axis morphogenesis in zebrafish embryos using robot-assisted tissue micromanipulation. *Nature Communications* **13**.
