# Supplementary material for: The Clock and Wavefront Self-Organizing model recreates the dynamics of mouse somitogenesis in vivo and in vitro
Source: Development. 2024 May 16;151(10):dev202606. doi: 10.1242/dev.202606 (PMC11165719; doi:10.1242/dev.202606)
Supplement: Supplementary file 1 [file develop-151-202606_review_history.pdf]

The Clock and Wavefront Self-Organizing model recreates the dynamics of mouse somitogenesis *in vivo* and *in vitro*

Julie Klepstad and Luciano Marcon
DOI: 10.1242/dev.202606

Editor: James Briscoe

Review timeline

Original submission:	14 December 2023
Editorial decision:	15 February 2024
First revision received:	22 March 2024
Accepted:	15 April 2024

Original submission

First decision letter

MS ID#: DEVELOP/2023/202606

MS TITLE: A Clock and Wavefront Self-Organizing Model Recreates the Dynamics of Mouse Somitogenesis in-vivo and in-vitro

AUTHORS: Julie Klepstad and Luciano Marcon

Apologies for the delay in getting back to you - one of the referees had several deadlines that delayed their response. I have now received all the referees' reports on the above manuscript, and have reached a decision. The referees' comments are appended below, or you can access them online: please go to BenchPress and click on the 'Manuscripts with Decisions' queue in the Author Area.

As you will see, the referees express considerable interest in your work, but have some significant criticisms and recommend a substantial revision of your manuscript before we can consider publication. Although each of the referees has a long list of points and questions, these are mostly requests for clarifications or suggestions to strengthen your arguments. The reports appear complementary and should be a helpful guide for revising your work. Reviewer 1 has a series of detailed questions that seek to clarify and understand model behaviour better. I would draw your attention to Reviewer 3's comments about the similarity to CGLE and encourage you to adjust your text accordingly. I also think Reviewer 2's suggestions to discuss the implication of your model for experimental testing is helpful.

If you are able to revise the manuscript along the lines suggested, I will be happy to receive a revised version of the manuscript. Your revised paper will be re-reviewed by one or more of the original referees, and acceptance of your manuscript will depend on your addressing satisfactorily the reviewers' major concerns. Please also note that Development will normally permit only one round of major revision. If it would be helpful, you are welcome to contact us to discuss your revision in greater detail. Please send us a point-by-point response indicating your plans for addressing the referees’ comments, and we will look over this and provide further guidance.

Please attend to all of the reviewers' comments and ensure that you clearly highlight all changes made in the revised manuscript. Please avoid using 'Tracked changes' in Word files as these are lost in PDF conversion. I should be grateful if you would also provide a point-by-point response detailing

© 2024. Published by The Company of Biologists under the terms of the Creative Commons Attribution License (https://creativecommons.org/licenses/by/4.0/).

how you have dealt with the points raised by the reviewers in the 'Response to Reviewers' box. If you do not agree with any of their criticisms or suggestions please explain clearly why this is so.

Reviewer 1

Advance summary and potential significance to field

In this work, Klepstad and Marcon present a compelling theoretical case for the integral role of cell-cell coupling in driving somitogenesis. They propose that an excitable self-organised mode of signalling upstream of the wavefront drives travelling phase-waves of signalling/gene-expression, and that anterior-posterior patterned parameters of this underlying signalling network lead to spatially organised dynamical modes: oscillation in the posterior, stable cell fate in the anterior (somite formation), and phase waves in between.

A key achievement that comprises the first half of the paper is the establishment of the “Sevilletor” model, a novel minimal reaction-diffusion (RD) framework that can generate five qualitatively different dynamical regimes. Dynamical systems theory analysis on this minimal model is exemplary, particularly in providing insights and intuition into how diffusion (or more generically, cell-cell coupling) can drive dynamical bifurcations; instabilities that are novel and expand the dynamical repertoire of RD models beyond the Turing model playbook.

This minimal RD framework allowed the authors to recast two existing models of somitogenesis (PORD and CG) as mechanisms that are structurally analogous but parametrically different, showing the broad scope of the Sevillator owing to its richness in dynamical regimes. This type of detailed work in mapping disparate mechanisms to a common mathematical framework is sadly not common in the field of theoretical developmental patterning, and the careful characterisation performed by the authors demonstrates the major benefits of such work: allowing for like-for-like comparison; and relating alternative mechanisms to different dynamical regimes of a similar system.

The authors then present an alternative model for somitogenesis, the Clock and Wavefront Self-Organising model (CWS). In contrast to the CG model, which they argue is comprised of a posterior oscillating (the clock) and an anterior bistable regime (the wavefront), the CWS model contains a third excitable regime in between. This excitable regime naturally gives phase waves driven by local self-organisation, as opposed to global gradients setting anterior-posterior phase differences in the CG model.

Comments for the author

The proposed alternative (CWS) model is an interesting extension of the CG model but I think that a few additional numerical experiments should be done to help:

(a) characterise better and provide a clearer intuition for how each of the mechanisms work; (b) how they differ; and (c) how tissue structure/size/growth inter-relates with the underlying dynamics to facilitate somitogenesis.

Gaining intuition for how the “cogs” of the model work together
----
While the Sevillator is beautifully minimal, its application to somitogenesis is rather more complex. This application involves the establishment of different spatially bounded parametric regimes, which are distinct among the different classes of model. A common underpinning model makes these comparisons tractable but the jump from the unbounded parametrically uniform simulations of Figs 1-3 and the complex set up in Fig 4 needs a bit more elaboration, so readers understand how the moving parts interact. It would be useful to devise some numerical experiments to understand how these spatially bounded dynamical regimes interact to give rise to the spatiotemporal phenomenology. Below I pose some questions and suggestions that could aid this connection from a generic minimal model to a more complex model of tissue patterning:

1. In CWS, the axis of phase wave propagation is orthogonal to the AP axis whereas in the unbounded case or in explant simulations, one gets circular or more exotic patterns. Is this due to

© 2024. Published by The Company of Biologists under the terms of the Creative Commons Attribution License (https://creativecommons.org/licenses/by/4.0/).

(a) boundary conditions of the tissue in the excitable regime (i.e. top-bottom in the plots in Fig 4); (b) the spatial profile of the transition from k1-low to k1-intermediate prescribed; (c) that of k1-intermediate to k1-high; or (d) something else. This could be addressed by: (a) enlarging the top-bottom axis of the rectangular grids and repeating simulations to a scale beyond the intrinsic wavelength of the excitable waves; and (b) slanting the spatial profiles of the k1-transition regions.

2. What determines the directionality of the phase-waves? If the oscillatory posterior is removed in rectangular simulations, does this directionality break-down? Or is posterior-growth somehow sufficient? Does the positioning of the bistable regime matter?

3. How does the choice of the sizes of the different regions of k1 influence the resultant pattern? A systematic sweep of sizes would provide some intuition (I appreciate this is done partially already in the posterior-extended mutant).
In the case of CG, how does the relative positioning of the transition point in k1 vs k3/4 influence the resultant pattern?

4. If k1 is interpreted to be controlled by some upstream signal, one could make the argument that the spatial shape of k1(x) is unrealistic. Given the excitable-bistable bifurcation, does a more reasonable spatial distribution (e.g. linear i.e. that of Fig S12, or exponential) lead to stable somite position above the bifurcation. Marking the bifurcation values in Movie 9 and Fig S12 would be useful here, and some quantification of somite positional stability would be good.

Noise
-----
1. In relation to noise arguments, Movie 8 suggests that while new cells are added at the posterior, new values of k1/k3/k4 are added at the anterior. Hence a given cell file will effectively sample the noise distribution over time, as the parameter distribution "moves" posteriorly. What happens if the amount of noise is fixed for a given absolute x? What happens if there are temporal fluctuations?

2. How robust is the model to cell movement, given especially posterior PSM cells undergo some degree of cell movement (at least in Zebrafish, 10.1038/s41586-018-0479-2 Fig. 3i)? Are there differences in robustness in different dynamical regimes? This may aid your noise arguments further.

Spatial scales

Only one of the species is diffusible in the model. In recent work (https://doi.org/10.1016/j.cels.2022.11.001) which considers a more canonical Turing network where only the repressor is diffusible, spatial discretisation is shown to be important in driving the patterning (where the spatial bins are assumed to be pseudo-cells, like in the work presented here). How does the choice of spatial discretisation affect the dynamics of patterning, especially in the CWS vs CG, but also PORD? Specifically: (a) if diffusion and the size of the spatial bins are re-scaled, does the model perform equivalently?; (b) do anterior-posterior travelling waves require spatial bins that are arranged perpendicular and orthogonal to this axis, i.e. would a hexagonal or a more random (e.g. off-hexagonal Voronoi) spatial discretisation scheme also work? If my intuition holds the excitable regime could help buffer this effective noise or conflicting structure in cell-cell coupling to allow for travelling waves regardless; and given the arrangement of cells in the PSM is non-epithelial and indeed locally irregular, it would be important to show how these models 'cope' with a more realistic tissue structure.

Arguments for CWS over CG

I think that while the CWS is conceptually appealing, the authors could try and make a clearer case for some qualitative differences in the two (or three) models.
It seems that there are some key arguments, for which I have some potential concerns/suggestions:

1. More travelling waves can be observed in the posterior-expanded mutant. (Given the nature of the referenced experimental perturbation, where many things could be going on, I think while the

© 2024. Published by The Company of Biologists under the terms of the Creative Commons Attribution License (https://creativecommons.org/licenses/by/4.0/).

explanation by the authors is suggestive, I would not hang the argument for discriminating CG vs CWS exclusively on this).

2. Fig 4 gives the impression that only the CWS can give the “phase-difference-gradient” between u/v (Wnt/Notch). Is this indeed true? Looking at the Movies, it seems that CG can also do this. What would be helpful here is (a) some systematic comparison of phase-difference-gradients among the different models, given some in the field believe this is a very important part of the phenomenology (doi.org/10.1038/nature11804 and 10.1016/j.cell.2018.01.026); and (b) specifically plotting phase vs x for u and v for each of the models so readers can do a side-by-side comparison.

It would aid the reader to have a summary near the end of the results section that re-iterates and details: (a) what are the mechanistic differences among the models; and (b) how do their predictions to various perturbations differ?
Some further discussion qualifying differences in experimental predictions from the different models, given potential caveats, would be helpful too.

Some other minor comments to aid understanding
----
1. Fig 5G: “Gradient” and “Dynamical behaviour” I believe is referring to k3/4 vs k1. Some annotation of this in the figure would aid the reader. Likewise, in the text, phase-values are referred to as being “inherited” from the embryo; I found this wording a bit hard to follow. Some textual description of the three ingredients and their relation to parameters and specific implementation would be helpful here (e.g. inheriting phase-values = initial condition). The authors do a better job in the supplementary text at describing this, so integrating some of this text into the main manuscript could be helpful. More generally, being careful to have a consistent terminology (e.g. ‘gradient’, PG, k3/4) throughout the text will help aid the transition to the explant explorations.

2. Some annotation of the simulation ‘domain’ in Fig 4 could aid understanding.
This would include some diagrammatic description of (a) where R and PG are, and their relation to k1/3/4; (b) how boundary conditions are implemented; (c) how growth is implemented (e.g. are posterior cells duplicated, vs ‘blank’ cells added).

3. Lines 582-590: in CWS, Wnt and Notch are out of phase in the posterior and in-phase in the middle. Can you elaborate on why this occurs: to me this is not an intuitive result, and given interest in phase-difference-gradients, this could be an important point for the uptake of this theoretical work in subsequent experimental studies.

4. Line 463-5: the wording here is quite unclear. Can this be recast in simpler terms to provide some intuition. The numerical experiments suggested above may bolster what I think the authors are trying to argue here as well.

Additional (non-essential) extensions that may aid the argument
----
1. In Movie 8 and 9, the gradient of k1 is not scaled to embryo size. This may indeed be reasonable, however there have been some suggestions of a scaled gradient (10.1242/dev.161257). How does scaling the entire gradient, or e.g. the oscillatory-to-excitable part affect the results?

2. If I understand the work correctly, the transition from oscillation/excitable phase-waves (CG vs CWS) to somite formation is due to change in bifurcation parameter (aka the wavefront). The spatial (and temporal) position of the bifurcation then locks in cell fate/establishes the somites. Does the speed of the progression of the (k1>bistablity bifurcation) vs speed of oscillations affect somite size/regularity of size? A phase diagram tuning each of these could be useful. Commenting on this in relation to doi:10.1126/science.1253089 could be helpful.

3. In figure S5, diffusion is shown to alter the spatial profile of waves in the unbounded case. How does the choice of diffusion coefficient influence (a) the spatial and (b) the temporal profile of phase waves in general, and in the ‘in-silico PSM’ (where the excitable regime is bounded by different regimes on each side).

© 2024. Published by The Company of Biologists under the terms of the Creative Commons Attribution License (https://creativecommons.org/licenses/by/4.0/).

4. When scrambling initial state (dissociated and re-aggregated explants), only in CWS do circular wave patterns emerge. Ultimately, this can be explained alternatively by internal establishment of long-range gradients (which could even be a purely passive process, given the spatial profile of an upstream diffusible signal that controls k3/4, e.g. FGF, as could be expected from uniform production diffusion, and leakage of the morphogen out of the perimeter of the circular explant).

Reviewer 2

Advance summary and potential significance to field

In their manuscript “A Clock and Wavefront Self-Organizing Model Recreates the Dynamics of Mouse Somitogenesis in-vivo and in-vitro”, Klepstad and Marcon present a new modelling framework for self-organizing dynamic processes in tissues. It is based on the combination of a reaction-diffusion system with negative feedback and longer range gradients, which they term the Sevillator. After an initial characterization and parameter testing, the model is used to investigate the process of somitogenesis, using experimental data from mouse embryos as baseline. This model can recapitulate certain experimental findings that other models could not, such as the clock and gradient (CG) model and progressive oscillatory reaction diffusion (PORD) model. This is a very well written manuscript that brings together several recent experimental findings in a new model and poses very interesting predictions that can be tested in future experiments.

Comments for the author

I have read the manuscript with a main focus on the biology and will not judge the mathematical implementation of the model, even though it makes sense to me.

There are a few points that should be clarified on the one hand or added to increase the usefulness for developmental biologists on the other hand.

- Several times throughout the manuscript, the authors present predictions of their model, some of which do not fit to available experimental data. The authors should follow up upon these further. These have two-fold relevance:
o On the one hand, the authors can speculate what their findings could mean for the biological process of somitogenesis.
Their implementation of the PORD model does not recapitulate certain experimental findings from the literature, such as the occurrence of multiple phase waves in beta catenin dominant active mice. In contrast, their new CWS model can.
Another example is the experimental finding that the phase-relationship between Wnt and Notch changes from out-of-phase to in-phase along the anterior posterior axis.
It would be interesting to discuss what we can learn from these differences.
o On the other hand, their model provides very concrete predictions that should be tested experimentally in the future.
One example is the finding that Wnt has to have a positive effect on Notch and Notch a negative effect on Wnt to allow the change in Wnt Notch phase-relationship along the tissue.
The authors could discuss these predictions more explicitly and make suggestions on what should be tested to support the validity of their model and this way understand the segmentation clock better.

- The authors use explants as experimental conditions to compare their new model to the PORD and CG model. In their analysis, the authors should keep in mind that gradients do reform during 2D culture which correlates with formation of phase waves (Lauschke et al.). Only in complex culture conditions with Wnt and FGF activation and BMP inhibition, oscillation dynamics of the explant or reaggregates are changed (Hubaud et al.). However, in these latter conditions also endogenous signalling gradients are changed. In their data (Figure 5), the authors consider all possibilities. However, the discussion of the biology and interpretation of their results should be adapted.
In general, in all their interpretation of data from Hubaud et al., the authors should consider that experiments were performed in “explant medium (dissection medium with 0.1mM of β-mercaptoethanol, Chir-99021 (Sigma) 3 μM, LDN-193189 (Sigma/Stemgent) 200nM, BMS-493 (Sigma)

© 2024. Published by The Company of Biologists under the terms of the Creative Commons Attribution License (https://creativecommons.org/licenses/by/4.0/).

2.5 µM, mFGF4 (R&D) 50ng/mL, heparin (Sigma) 1 µg/mL and Y-27632 (P212121/Tocris/Selleckchem) 10 µM)", while in Lauschke et al. explants were cultured DMEM F12 without any small molecules.

- The authors should be careful when interpreting experimental findings that inhibition of Notch signalling "arrests oscillations" (line 635, Figure 5C). The reporter Luvelu is not only a reporter for oscillations but also a direct target gene of Notch signalling. However, in the framework of their interpretation their data makes sense.

- There are a few aspects mentioned in the introduction that should be rewritten or added. These mostly do not have an impact on the model or the results section, but should be changed.
o Line 45: Add Takashima et. al PNAS 2011 as reference for delayed feedback.
o Line 46 and following: "The specific mechanism responsible for the spatial synchronization of oscillations, however, remains a subject of debate." Is not entirely correct, as there is evidence that Notch signalling is responsible for the synchronization between cells. I think the authors mean the tissue-wide pattern. That should be rewritten to make clear. In addition, references should be added to the two possibilities mentioned in lines 48 and 50.
o Line 80 etc.: "Moreover, experimental evidence shows that posterior signaling gradients of Wnt and Fgf allow cells to oscillate at the posterior tip of the tail." Is a too strong statement. There is indeed a correlation between gradients and oscillations. Even though there is experimental evidence that gradients influence the waves, as the authors also discuss in the paper, how exactly they are linked and whether the gradients are necessary for the oscillations still has to be investigated. The authors should rephrase it.
o Lines 123 and following: it has been shown in both explant cultures and reaggregates that signalling gradients are re-established (Lauschke et al. 2013, Tsiairis et al. 2015). In fact, in reaggregates this correlates with the transition from in-phase oscillations to waves. I agree with the authors' conclusion that PSM cells have the capacitiy to self-organize, however this cannot be separated from the spatial gradients. This aspect also has implications for their modelling data here.

- Besides changes in the phase shift between Wnt and Notch signalling, there is also an increase in oscillation amplitude from posterior to anterior. It would be interesting to investigate how amplitude changes along the tissue depending on AP position. However, this might be beyond the scope of this study, which I cannot judge properly.

Minor:
- In lines 818 following the authors discuss oscillations in single cells. In this discussion, the authors should also mention the possible influence of cellular mechanics on signalling oscillations. As the authors write, when single mouse cells are cultured on BSA, they oscillate, which cells don't do when adherent.

- The authors discuss that there Sevillator can be used to model other aspects in biology/ developmental biology. It would be useful if the authors could expand on this further and give examples.

- Fig. 2C: Symbols are too small to discern properly. The same is true for Fig. 4B, where also the legend is missing.

- Fig. 5G-N: It might be useful to restructure this whole part of the figure to indicate, what the input conditions (Fig.5D) and the results of each model were (Fig. 5 H-J). Right now, the figure is difficult to read, in my opinion.

<u>Reviewer 3</u>

Advance summary and potential significance to field

The manuscript "A Clock and Wavefront Self-Organizing Model Recreates the Dynamics of Mouse Somitogenesis in-vivo and in-vitro" by Klepstad and Marcon presents a minimal dynamical system model, based on two coupled partial differential equations, that they have termed the Sevilletor.

© 2024. Published by The Company of Biologists under the terms of the Creative Commons Attribution License (https://creativecommons.org/licenses/by/4.0/).

The model is remarkable, because tuning very few parameters it is able to produce very different dynamical regimes relevant for segmentation clock studies: fixed points, bistability, relaxation and excitable oscillations. This is the beauty and the strength of this model: it allows to very cleanly transition between this different dynamical regimes, and spatial variations of the model parameters can produce a wealth of dynamics reminiscent of observations in different settings of segmentation clock studies. I therefore think that this setting can be a powerful tool to gain understanding on the underlying mechanisms of segmentation clock dynamics in different organisms and settings, and therefore recommend publication.

However, I have two main comments about the origin of the model and its relation with previous research, and the tone that the manuscript has in some paragraphs.

Comments for the author

Eqs. 1-2 are very closely related to the complex Ginzburg-Landau equation (CGLE) with broken gauge invariance, see Aranson and Kramer for a review of the state of the art in 2002. The CGLE with broken gauge invariance is in general of an excitable nature, and it shows transitions from oscillations to bistability (actually, varying the parameter gamma in Eq. 86 of Aranson and Kramer is quite equivalent to varying the difference between k1 and k2 in the current manuscript).

It has very rich dynamics, and I would be utterly surprised if the dynamical behaviors described in the manuscript have not been observed or described in the CGLE framework, for which an extensive literature exists. This connection should be made in the manuscript, and, importantly, I am convinced that assertions like that of line 239 ("This excitable self-organizing patterning behavior has not been described previously") do not hold. Moreover, pattern formation in excitable media has been a very intensive field of research at least since the 80s, where all kinds of imaginable phenomena have been observed. Already in 1988 Tyson and Keener wrote a review (with 999 citations to this day) where they discuss the generic properties of systems of equations of the form:
u_t = D1*Delta u + f(u,v)
v_t = D2*Delta v + g(u,v),
which includes Eqs. 1-2 of this manuscript. A vast literature exists studying this type of models, some of them very close to the model proposed. However, a casual reader of the manuscript would not be aware of this. In line 166 the authors recognized that the phenomena in their model has been observed before, but the single reference to a low-profile recent work (43) does not convey the huge amount of work existent in this field. Another reference is made in the methods, (44), and together with some citations to founding papers of out-of-equilibrium studies that is all. I understand that the goal here is not the review the field of pattern formation in excitable systems, but a clear presentation of the background from which this model is formulated should be given.

The authors have chosen to call "models" the different dynamical regimes that the Sevilletor equations can reach when certain values of the parameters or their spatial dependence are implemented. This can be somehow confusing, specially since specific implementations of this "models" studied in the manuscript do not cover the whole space of phenomena that the family of published works related to these regimes can show, nor can some of the conclusions extracted here from Sevilletor simulations be generalized so easily as sometimes implied. Also, the intention of extending segmentation clock models to describe latest experimental developments like in vitro explants and somitoids is commendable, but the tone (previous efforts being wrong, when in fact they were proposed for more restricted settings; observations of the model being firsts, when references to the rich physics literature studying these kinds of dynamical systems are missing) can definitely be improved. Seasoned theoreticians familiar with segmentation clock models and a broad knowledge of dynamical systems would be able to see through this tone what is really going on, a real danger exists of misleading experimentalists not so familiar with technical aspects. With a little editing here and there, this issue can be easily corrected.

Some more small comments:

Line 97: "In essence, in the CG model, both the arrest of oscillations and the formation of phase waves are under the control of global signals".
This is a subtle point, as discussed in most of the references that propose this model. In them, a tissue-wide frequency profile is proposed, true, but the origin of this profile is not a part of the

© 2024. Published by The Company of Biologists under the terms of the Creative Commons Attribution License (https://creativecommons.org/licenses/by/4.0/).

model. Global signals, e.g., signaling gradients are usually discussed, but other possibilities, as self-organization are also consistent with these theoretical formulations. Note that these models were formulated for the geometry of the PSM, and were never intended to discuss in vitro or organoid experiments. Efforts tackling these new systems, as the manuscript under consideration, are necessary.
The time derivatives in Eqs. 1-2 should be partial derivatives, at least if the Laplacian has to be read in the continuous sense.
The value D=0.3 is used, but only mentioned in the methods. It would be useful to mention it also in the caption of Figure 2, where values are given for other parameters. The sizes of squares in this figure and Figure 3 are given in methods but it would also be helpful to have them in the captions.

Lines 2356 and 2357 in Methods: there is a typo. u(x,y,t+1) should be u(x,y,t+dt), and v(x,y,t+1) should be v(x,y,t+dt). It would also be nice to say here that the values of dt used at particular simulations will be given below.
Parameters: if the time step used for the somitogenesis models is the same dt=0.002, why is the frequency of adding a new row of cells during the somitogenesis simulation in a virtual tail different for each model?

Line 404: “However, applying noise to the posterior gradient PG causes somite pattern disorganization over time (Figure 4J and Figure S9)”.
The “noise” in different models is difficult to compare. According to the supplement, 0.5% noise is added to the parameters k1, k3, and k4 every time a new row of cells is added on the posterior side. But in absolute value, this implies wider differences for model implementations that use higher values of the constants, and therefore not only the results of noise, but what could be considered comparable noise situations, is very subtle. I recommend a more nuanced and careful discussion.

Line 406: “Finally, the model predicts a loss of phase waves in mutants with posterior signal expansion and flattening, contrary to experiments [3] (Figure 4I)”. This is a misleading representation of what the Clock and Gradient model can do. Reference [3] studies mutant beta-catenindel(ex3)/+ mice embryos, the conclusion, expressed in the title, is that a beta-catenin gradient links the clock and wavefront systems in mouse embryo segmentation. Certainly then, in the framework of the Clock and Gradient model, the effect of the beta-catenindel(ex3)/+ mutation would not be limited to an expansion of the PSM: a change of the shape of the gradient would be certainly expected. These are phenomenological models, and therefore it is difficult to find phenomena that they cannot reproduce. The simulation of figure 4I shows an expanded PSM with a very flat gradient, abruptly decaying in the anterior PSM. A gradual decay from posterior to anterior would reproduce the phase wave observed in the experiments scheme in Figure 3i of [3]. Regarding this mistake in model interpretation, I would like to stress again that a wonderful thing about the model proposed is its power to reproduce different mechanisms in a single framework, but with great power comes great responsibility and care has to be taken before reaching conclusions.

Line 482: “providing a possible explanation for the multiple phase waves observed upon expansion and flattening posterior gradients in mouse mutants (Figure 4L Movie 8) [3]”. This is true, and nice, but recall the previous comment that other model formulations can also reproduce this observation, and that it is because a flawed formulation that this was not recapitulated in the manuscript.

Line 495: “the somite pattern formed by the CWS is also robust to noise applied to parameter values. Indeed, the model can be implemented with any values of $0 < k1 \leq 2.3$ for the posterior oscillatory region and any values of $k1 > 4$ in the anterior region”. As discussed above, I believe this comparison is not straightforward, and recommend a more nuanced discussion. Note also “same amount of noise” in line 502.

Typo: Ref. 21 is incomplete.

References

© 2024. Published by The Company of Biologists under the terms of the Creative Commons Attribution License (https://creativecommons.org/licenses/by/4.0/).

Aranson, I. S., & Kramer, L. (2002). The world of the complex Ginzburg-Landau equation. Reviews of modern physics, 74(1), 99.
Tyson, J. J., & Keener, J. P. (1988). Singular perturbation theory of traveling waves in excitable media (a review). Physica D: Nonlinear Phenomena, 32(3), 327-361.

First revision

Author response to reviewers' comments

Dear Dr. Briscoe

We are extremely grateful to the Development editorial board and the three reviewers for having received such high quality and detailed comments. We believe that the feedback has contributed to a substantial improvement of the manuscript. It has been our pleasure to revise the manuscript following the advice and constructive criticisms of reviewers.

Specifically, we have added a more detailed discussion of the biological relevance and we now include more predictions that can be tested experimentally as suggested by reviewer 2. As highlighted by reviewer 3, we have provided a more comprehensive discussion of seminal theoretical works such as the CGLE in the field of patterning dynamics and how they relate to the Sevilletor model. According to the comments of reviewer 1, we have also done significant amounts of work to better clarify and understand how the different components of the models work, and how the underlying dynamics create the global patterning behaviors. The numerical simulations and analyses requested by reviewer 1 are now presented in Figures S3, S4, S11, S12, S14, S16, S17, S18, S19, S20, S21, S22, S24, S25, S26 and S32.

We have also edited the manuscript, figures, supplementary materials and movies to comply with the formatting guidelines, and we have reduced the length of the manuscript to 7700 words as the editorial administrator said would be accepted at this stage of the revision process. We submit the manuscript as a tex file with all source files and packages needed to compile the document included in the folder. Please let us know if there are any further changes needed and we will be happy to provide them.

Overall, we believe the manuscript has markedly improved, both in presenting and discussing theoretical results, and in contextualizing our hypothesis with published experimental data. We're excited about the prospect of communicating our study in Development and we are confident that our study will contribute to the ongoing exciting debate about the possible theoretical explanations of somitogenesis.

Thank you once again for your support and for your remarkable work with the Development journal. Below we present a point by point response to reviewers.
We look forward to hearing from you again.

Julie Klepstad and Luciano Marcon

Reviewer 1 Advance Summary and Potential Significance to Field:
In this work, Klepstad and Marcon present a compelling theoretical case for the integral role of cell-cell coupling in driving somitogenesis. They propose that an excitable self-organised mode of signalling upstream of the wavefront drives travelling phase-waves of signalling/gene-expression, and that anterior-posterior patterned parameters of this underlying signalling network lead to spatially organised dynamical modes: oscillation in the posterior, stable cell fate in the anterior (somite formation), and phase waves in between.

A key achievement that comprises the first half of the paper is the establishment of the "Sevilletor" model, a novel minimal reaction-diffusion (RD) framework that can generate five

© 2024. Published by The Company of Biologists under the terms of the Creative Commons Attribution License (https://creativecommons.org/licenses/by/4.0/).

qualitatively different dynamical regimes. Dynamical systems theory analysis on this minimal model is exemplary, particularly in providing insights and intuition into how diffusion (or more generically, cell-cell coupling) can drive dynamical bifurcations; instabilities that are novel and expand the dynamical repertoire of RD models beyond the Turing model playbook.

This minimal RD framework allowed the authors to recast two existing models of somitogenesis (PORD and CG) as mechanisms that are structurally analogous but parametrically different, showing the broad scope of the Sevillator owing to its richness in dynamical regimes. This type of detailed work in mapping disparate mechanisms to a common mathematical framework is sadly not common in the field of theoretical developmental patterning, and the careful characterisation performed by the authors demonstrates the major benefits of such work: allowing for like-for-like comparison; and relating alternative mechanisms to different dynamical regimes of a similar system.

The authors then present an alternative model for somitogenesis, the Clock and Wavefront Self-Organising model (CWS). In contrast to the CG model, which they argue is comprised of a posterior oscillating (the clock) and an anterior bistable regime (the wavefront), the CWS model contains a third excitable regime in between. This excitable regime naturally gives phase waves driven by local self-organisation, as opposed to global gradients setting anterior-posterior phase differences in the CG model.

We want to express our gratitude to the reviewer for writing such a comprehensive summary of our manuscript and we are delighted to see that they appreciate our work and that they have gained a profound understanding of it.

Reviewer 1 Comments for the Author:
The proposed alternative (CWS) model is an interesting extension of the CG model, but I think that a few additional numerical experiments should be done to help: (a) characterise better and provide a clearer intuition for how each of the mechanisms work; (b) how they differ; and (c) how tissue structure/size/growth inter-relates with the underlying dynamics to facilitate somitogenesis.

We completely agree with the reviewer that it was necessary to bridge the gap between the dynamical systems and somitogenesis parts of the paper. In the following pages we will describe how we have followed the reviewers comments and suggestions to change the text and add more numerical experiments to better explain how the different models work. In particular, we improved the Result and Discussion sections and we expanded Figure 4 by adding a time course of the Clock and Wavefront (CW) model to illustrate how growth is included in the simulations. Additionally, we show how the parameters k1/k3/k4 relate to the regionalization (R) and frequency gradient (FG, previously called posterior gradient PG). We also include the phase spaces from Figure 2F that characterize the behaviors in the different zones defined by R and FG in the plots where the models are presented in Figure 4E,H,K.

Finally, following all the reviewer comments we have added Figures S11, S12, S14, S16, S17, S18, S19, S20, S21, S22, S24, S25 and S26 to show how the tissue size, growth, cell movement, changes in the proportions of the different regions, diffusion and spatial discretization affect the models.

Gaining intuition for how the “cogs” of the model work together
----
While the Sevillator is beautifully minimal, its application to somitogenesis is rather more complex. This application involves the establishment of different spatially bounded parametric regimes, which are distinct among the different classes of model. A common underpinning model makes these comparisons tractable, but the jump from the unbounded parametrically uniform simulations of Figs 1-3 and the complex set up in Fig 4 needs a bit more elaboration, so readers understand how the moving parts interact. It would be useful to devise some numerical experiments to understand how these spatially bounded dynamical regimes interact to give rise to the spatiotemporal phenomenology. Below I pose some questions and suggestions that could aid this connection from a generic minimal model to a more complex model of tissue patterning:

© 2024. Published by The Company of Biologists under the terms of the Creative Commons Attribution License (https://creativecommons.org/licenses/by/4.0/).

1. In CWS, the axis of phase wave propagation is orthogonal to the AP axis, whereas in the unbounded case or in explant simulations, one gets circular or more exotic patterns. Is this due to (a) boundary conditions of the tissue in the excitable regime (i.e. top-bottom in the plots in Fig 4); (b) the spatial profile of the transition from k1-low to k1-intermediate prescribed; (c) that of k1-intermediate to k1-high; or (d) something else.

This could be addressed by: (a) enlarging the top-bottom axis of the rectangular grids and repeating simulations, to a scale beyond the intrinsic wavelength of the excitable waves; and (b) slanting the spatial profiles of the k1-transition regions.

We thank the reviewer for bringing up this important point, which allowed us to clarify the reason for the propagation of the waves along the AP axis. This is due both to the boundary of the neighboring posterior region with k1=1, belonging to the (b) case, and to the initial phase values that belong to the (d) case. We expand on each case below:

(a) In Figure S18 we show that varying the width of the tail relative to the length: from very thin (almost 1D) to very thick (several times the wavelength) does not affect the patterning behavior of the CWS model.

(b) Starting from noisy initial conditions, the periodic waves generated by the Sevilletor equation for k1=2.3 propagate in any direction (as shown in Figure 2E). In contrast, within the intermediate excitable region of the CWS model (k1=2.3), the propagation of waves is guided by homogeneous oscillations at the posterior tailbud (k1=1), which act as a pacemaker promoting waves that propagate orthogonally across the AP axis (lines 383-400).

This guiding influence does not depend on the specific slope of the transition from k1=1 to k1=2.3 and does not have to be abrupt. Indeed, in Figure 4Q, we present a version of the CWS model with a linearly increasing value of k1 (Movie 9), demonstrating that the CWS model functions effectively even with a graded change of k1. Moreover, we find that the shape of this transition along the y-axis (from top to bottom) does not need to be perfectly straight to propagate straight waves along the anterior-posterior axis. Indeed, we still observe straight wave propagation even in the presence of random cell movement (Figure S17) and in simulations with a hexagonal lattice (Figure S26). However, it is indeed the overall shape and position of the pacemaker population that determines the shape of the waves. This can be seen in the explant of the CWS model shown in Movie 11 and Figure 5L, where a central rounded population of pacemaker cells promotes circular waves that propagate outwards.

(c) As in the case of the transition from k1=1 to k1=2.3, the transition from k1=2.3 to k1=4 can be gradual with noise along the y-axis without affecting the orthogonal wave propagation (Figure 4Q, Movie 9, Figure S17 and Figure S26).

(d) In general, the shape of the waves formed in the excitable region is also affected by the phase value distribution. In explants obtained only with the middle section of the tail, which does not include the pacemaker, the waves can still propagate in a circular manner following the distribution of phase values inherited from the tail (Movie 12, Figure 5M). This can also be seen in explants where the central pacemaker population is ablated (Figure S29). However, in both scenarios, the absence of guiding cues results in greater variability in wave patterns, which is primarily determined by the specific spatial distribution of phase values. We observe either circular waves that emanate from spiral-like centers and propagate indefinitely, or waves that emanate from the center and dissipate into a homogeneous pattern (Figure S31).

2. What determines the directionality of the phase-waves? If the oscillatory posterior is removed in rectangular simulations, does this directionality break-down? Or is posterior-growth somehow sufficient? Does the positioning of the bistable regime matter?

Similar to the case discussed above, the directionality of the phase waves in the excitable region is guided by the oscillation in the posterior region. However, once a pattern is formed, the spatial distribution of phase values is sufficient to keep propagating the waves anteriorly. In the previous version of the manuscript in Figure S14A, we already showed that the phase waves keep

© 2024. Published by The Company of Biologists under the terms of the Creative Commons Attribution License (https://creativecommons.org/licenses/by/4.0/).

propagating in the same direction even after the posterior oscillatory region is dissected. This happens due to distribution of phase values and in the absence of growth in the anterior dissected region. In the new version of the manuscript, we added another simulation where we stopped the growth of the posterior dissected part and the progression of the bistable region, shown in Figure S20B. In this case, we can still observe that phase waves propagate anteriorly thanks to the initial distribution of phase values. Finally, we observe that the positioning of the transition from oscillatory to excitable regime does not affect the directionality of the waves and the somitogenesis pattern, as we show in Figure S19 varying the size of the posterior oscillatory region.

3. How does the choice of the sizes of the different regions of k1 influence the resultant pattern? A systematic sweep of sizes would provide some intuition (I appreciate this is done partially already in the posterior-extended mutant). In the case of CG, how does the relative positioning of the transition point in k1 vs k3/4 influence the resultant pattern?

As mentioned above, varying the transition point from oscillatory to excitable in the CWS model does not affect the directionality of the waves and the somitogenesis pattern, as we now show in Figure S19. Moving the transition point from excitable to bistable in the CWS gives rise to a larger region where multiple phase waves are formed, as we now show in Figure S21F.

Following the comments of the reviewers we now perform a comprehensive sweep of different R (k1) and FG (k3/k4) in the CG model (Figure S21D,G-J). Here we show that changing the relative positions of the transition points in R and FG will lead to a loss of phase waves when FG is extended posteriorly while R is unchanged (Figure S21G), and in the opposite case more phase waves are formed (Figure S21H). We also show that the CG can generate multiple phase waves with a linearly decreasing FG and an expanded R region (Figure S21I), but that it forms no waves with a flat FG profile (Figure S21J). This highlights that CG models require a frequency gradient to form phase waves.

4. If k1 is interpreted to be controlled by some upstream signal, one could make the argument that the spatial shape of k1(x) is unrealistic. Given the excitable-bistable bifurcation, does a more reasonable spatial distribution (e.g. linear, i.e. that of Fig S12, or exponential) lead to stable somite position above the bifurcation. Marking the bifurcation values in Movie 9 and Fig S12 would be useful here, and some quantification of somite positional stability would be good.

The function R that controls changes in k1 within the CG and CWS presented in Figure 4H,K represent simple regionalizations of the tail. These types of regionalizations are common during development and can be established by interpreting a threshold of an upstream signal. We now show that in the CWS model, these regions do not require specific slopes or sizes for the overall dynamics to form. As it can be appreciated comparing Figure 4K (Movie 8) and Figure 4Q (previously Figure S12, Movie 9), even with a dramatic difference from a step-like distribution of k1 to a linear distribution, the stability of the somite pattern is not affected. We also thank the reviewer for the suggestion of marking the transitions between the regions of different behaviors in the CWS model with a linear change in k1, as this has made the simulation more easy to compare with the CWS with a step-wise change in k1.

Noise
-----
1. In relation to noise arguments, Movie 8 suggests that while new cells are added at the posterior, new values of k1/k3/k4 are added at the anterior. Hence a given cell file will effectively sample the noise distribution over time, as the parameter distribution "moves" posteriorly. What happens if the amount of noise is fixed for a given absolute x? What happens if there are temporal fluctuations?

That is correct. As the tail grows, new cells are added posteriorly, while R and FG (k1/k3/k4) effectively "grow" on the anterior side. A given cell file will pass by different values of R and FG where the accumulated noise will differ, but on average the same amount of noise is uniformly distributed in space. To make sure that all cell files have an (on average) equal amount of accumulated noise in the parameters k1/k3/k4, we add noise to the complete arrays of the

© 2024. Published by The Company of Biologists under the terms of the Creative Commons Attribution License (https://creativecommons.org/licenses/by/4.0/).

parameters (x=1 to L(t=final)) during the whole simulation. In other words, the anterior parts of R and FG that are not yet visible in the simulations are also accumulating multiplicative noise, to make sure that the effect of noise does not decrease as cells move anteriorly.

A simulation where the amount of noise is fixed for a given absolute file gives equivalent results (data not shown). We have also performed numerical simulations by adding temporal fluctuations in the amplitude of the noise in Figure S16 and with cell movement in Figure S17. Both simulations show that the CWS model has a general good robustness to noise even when cells move or experience bursts of noise.

The amplitude of the noise with temporal fluctuations is sampled from a uniform distribution between 1% to 10% for phase values, and 0.1% to 1.0% for parameters k1/k3/k4. We have now added a section called "Noise" in the Methods Section where we describe in detail how noise is added to the different models. We have also added Tables 1-7 in the Methods Section where all the parameters used in every simulation presented in the manuscript and the supplementary are presented, including the parameters used for adding noise.

2. How robust is the model to cell movement, given especially posterior PSM cells undergo some degree of cell movement (at least in Zebrafish, 10.1038/s41586-018-0479-2 Fig. 3i)? Are there differences in robustness in different dynamical regimes? This may aid your noise arguments further.

We thank the reviewer for this great suggestion which we have implemented for the CG and CWS model in Figure S17, where the movement of cells is simulated as a swap between the positions of two neighboring cells. At each timestep, each cell will switch positions with a random neighbor with a probability Pswap = 0.005. This results in about 500 swaps per dt in both models. The result is that both the CG and the CWS are generally robust to cell movements in all three behaviors: oscillations, excitability and bistability. However, as seen in the timelines presented in Figure S17, the oscillatory regions are less affected by the movement of the cells than the bistable regions. This is due to the fact that the cells can easily re-synchronize around an unstable steady state, while in a bistable regime the cells need to destabilize neighboring cells with noisy values of u and v to re-establish the organized pattern of straight lines. This relates nicely with the experimental observations in Mongera et al. 2018 (https://doi.org/10.1038/s41586-018-0479-2), where cells have a more fluid-like behavior in the most posterior tailbud (MPZ) and a solid-like behavior in the rest of the PSM in Zebrafish. This explanation is included in the caption of Figure S17 and we discuss these results in relation to robustness to noise in lines 406 and 416.

Spatial scales

Only one of the species is diffusible in the model. In recent work (https://doi.org/10.1016/j.cels.2022.11.001) which considers a more canonical Turing network where only the repressor is diffusible, spatial discretisation is shown to be important in driving the patterning (where the spatial bins are assumed to be pseudo-cells, like in the work presented here). How does the choice of spatial discretisation affect the dynamics of patterning, especially in the CWS vs CG, but also PORD? Specifically: (a) if diffusion and the size of the spatial bins are re-scaled, does the model perform equivalently?; (b) do anterior-posterior travelling waves require spatial bins that are arranged perpendicular and orthogonal to this axis, i.e. would a hexagonal or a more random (e.g. off-hexagonal Voronoi) spatial discretisation scheme also work? If my intuition holds, the excitable regime could help buffer this effective noise or conflicting structure in cell-cell coupling to allow for travelling waves regardless; and given the arrangement of cells in the PSM is non-epithelial and indeed locally irregular, it would be important to show how these models 'cope' with a more realistic tissue structure.

This is a very interesting topic to discuss that we thank the reviewer for bringing up. We have added Section S8 with Figures S11 and S12 to present these findings to the readers.

(a) Yes, indeed our simulations are made on a discrete grid that consists of pseudo-cells, where as shown in Wang et al. 2022 (https://doi.org/10.1016/j.cels.2022.11.001), how space is discretized affects the outcome of the simulations. In Figure S11 we perform a sweep of the diffusion constant D and the diameter of the cells dx for square simulations with excitable

© 2024. Published by The Company of Biologists under the terms of the Creative Commons Attribution License (https://creativecommons.org/licenses/by/4.0/).

(k1=2.3) or lateral inhibition (k1=0) behaviors. Here we see that both behaviors are robust to re-scaling both D and dx (simulations of the diagonal from top left to bottom right). In addition, we observe that the excitable behavior is more robust to variations in the ratio between D and dx than the lateral inhibition pattern. Indeed, the excitable behavior requires only dx to be sufficiently small relative to D to allow for excitation, while the lateral inhibition case requires a more specific balance between dx and D to stop oscillations in chessboard pattern.

In the simulations of the tails in Figure S12 we show that all three models (PORD/CG/CWS) perform equivalently when D and dx are re-scaled when the ratio between the two is kept constant at sqrt(D)/dx. I.e, bigger cells need stronger diffusion to perform the same behaviors.

(b) As the reviewer says, in a more realistic tissue structure the self-organizational properties of the patterning behaviors in the CG and CWS compensate for perturbations in the specific shape of the discretization of the lattice. This is illustrated in Figure S26 where we show that phase waves will form and propagate as usual in the CWS model in a 2D grid of hexagonal cells. The model is also robust to other realistic perturbations, such as when noise is included in the form of cell movement (Figure S17). In the new figures we have also shown that perturbations in the specific model properties set by us does not disrupt the overall patterning behaviors of the CWS:

- Proportions of the tail size (Figure S18)
- Size of the oscillatory tailbud (Figure S19)
- Growth rate (Figure S25)
- Shape of R (k1) (Figure 4Q and Movie 9)
- Temporal fluctuations in noise (Figure S16)
- Variations in D and addition of diffusion of v (Figure S22)
- Re-scaling of spatial discretization and D (Figure S12)

For the PORD however, changes in the tissue structure could affect the overall patterning to a higher degree, because as we have shown in the new Figure S14, both the original PORD model by Cotterell et al. 2015 (https://doi.org/10.1016/j.cels.2015.10.002) and the Sevilletor implementation of the PORD model are sensitive to noise, consistent with studies by Pantoja-Hernández et al. 2021 (https://doi.org/10.1063/5.0045460).

Arguments for CWS over CG

I think that while the CWS is conceptually appealing, the authors could try and make a clearer case for some qualitative differences in the two (or three) models.

It seems that there are some key arguments, for which I have some potential concerns/suggestions:

1. More travelling waves can be observed in the posterior-expanded mutant. (Given the nature of the referenced experimental perturbation, where many things could be going on, I think while the explanation by the authors is suggestive, I would not hang the argument for discriminating CG vs CWS exclusively on this).

We agree with the reviewer that, although the stabilization of beta catenin shown in Aulehla et al. 2008 (https://doi.org/10.1038/ncb1679) leads to the saturation and expansion of Wnt and Fgf signaling targets, there are various aspects of somitogenesis that can be affected in this mutant. Importantly, we now clarified that the main aim of our study is not to favor one model over another, but rather to employ the Sevilletor equations to illustrate how the main dynamical patterning regimes of the models can replicate distinct qualitative behaviors of the PSM. Specifically, we emphasize that the excitable regime of the CWS model can recapitulate the behavior of the mouse PSM observed in-vitro. In addition, we mention that the features of the CG and CWS models are not mutually exclusive, now mentioned in the Discussion Section in lines 626-630.

Following the reviewer's comment, we relocated the simulations with extended posterior gradients to the supplementary section (Figure S21) where we now perform a more comprehensive perturbation of R and FG in the CG and CWS model. Here, we speculate that the most direct comparison of the extended gradients of Pea3 (FGF signaling) and Axin2 (WNT

© 2024. Published by The Company of Biologists under the terms of the Creative Commons Attribution License (https://creativecommons.org/licenses/by/4.0/).

signaling) observed in Aulehla et al. (2008) would be the case where R and FG are extended anteriorly, which in the CG model shows a loss of phase waves (Figure S21D). However, we also show that under different perturbations of R and FG, the CG model can also give rise to multiple phase waves (Figure S21H-I). A key difference in qualitative behavior of the CG and the CWS model is the case without a frequency gradient, where the CG model forms no waves (Figure S21J) and the CWS can form multiple waves (Figure S21F).

2. Fig 4 gives the impression that only the CWS can give the "phase-difference-gradient" between u/v (Wnt/Notch). Is this indeed true? Looking at the Movies, it seems that CG can also do this. What would be helpful here is (a) some systematic comparison of phase-difference-gradients among the different models, given some in the field believe this is a very important part of the phenomenology (doi.org/10.1038/nature11804 and 10.1016/j.cell.2018.01.026); and (b) specifically plotting phase vs x for u and v for each of the models so readers can do a side-by-side comparison.

We agree with the reviewer that this should be more clearly presented in the manuscript so we have added plots of the average phases of u and v along the anterior-posterior axis for the PORD/CG/CWS models in Figure 4F,I,L. Here we show that for the Sevillletor implementations of the three models, only the CWS model has phase waves without a phase-shift, while the CG model has a phase shift. We show a detailed analysis of this in Figure S24, where the dynamics of the CG and CWS models are compared, showing that it is the change in the limit cycle shape promoted by excitability that allows the CWS to synchronize the phase between Wnt and Notch. This is not possible by just changing the frequency along the anterior-posterior axis, as assumed by a traditional CG model. We present a more detailed explanation on the underlying mechanisms in the answer to the reviewer's 3. question in the section below that starts with "Some other minor comments to aid understanding".

In the current manuscript, we assert that the CWS model effectively captures the change in phase along the anterior-posterior axis (lines 462-470). We do not state that the PORD and CG models cannot achieve this. Indeed, as correctly pointed out by reviewers, there may exist alternative implementations of these models that are capable of accounting for the phase change. We greatly appreciate the feedback provided by the reviewers, which has contributed to enhance our manuscript.

It would aid the reader to have a summary near the end of the results section that re-iterates and details: (a) what are the mechanistic differences among the models; and (b) how do their predictions to various perturbations differ?
Some further discussion qualifying differences in experimental predictions from the different models, given potential caveats, would be helpful too.

(a) We have added a paragraph in the Discussion (lines 660-671) that highlights the main difference between the CG and CWS models. Specifically, we say that in the CG model waves arise within an oscillatory regime promoted by frequency modulation along the anterior-posterior axis, while in the CWS model, waves emerge at the interface between oscillation and bistability. We have also expanded Figure 4 to better distinguish the differences in the three models by illustrating how the parameters k1/k3/k4 relate to the regionalization (R) and frequency gradient (FG). To show the mechanistic differences between the models we have added the phase spaces from Figure 2F that characterize the behaviors in the different zones defined by R and FG in the plots where the models are presented inFigure 4E,H,K.

(b) We have also discussed more in detail the experimental predictions arising from each model's patterning mechanisms, emphasizing their ability to predict specific observations without necessarily viewing the models as mutually exclusive. In this analysis we consider robustness to noise, ensuring that all models are exposed to comparable amounts of noise (Methods Section "Noise"). We found that our PORD implementation mirrors the original model's fragility to noise due to an underlying lateral inhibition patterning (Figure S14). The CG and CWS models exhibit comparable robustness, but the CG model is more sensitive to frequency gradient changes. Further, we explore distinct behaviors of the CG and CWS models with changes in R and FG zones (see Figure S21), highlighting the CWS model's ability to generate phase waves without frequency gradients (Figure S21F). The notable qualitative difference among the PORD, CG, and CWS models

© 2024. Published by The Company of Biologists under the terms of the Creative Commons Attribution License (https://creativecommons.org/licenses/by/4.0/).

lies in the behavior of isolated PSM cells in vitro. While PORD and CG predict oscillatory behavior, the CWS model suggests diffusion-driven excitable behavior. In Supplementary Section S3, we elaborate on the difference between the CWS model and previous excitable models. Finally, in the section titled "The excitability of the CWS model recapitulates the behavior of the mouse PSM in-vitro" in the manuscript we present two newly added novel predictions linked to the CWS model's excitable behavior (lines 518-546).

Some other minor comments to aid understanding
----

1. Fig 5G: "Gradient" and "Dynamical behaviour" I believe is referring to k3/4 vs k1. Some annotation of this in the figure would aid the reader. Likewise, in the text, phase-values are referred to as being "inherited" from the embryo; I found this wording a bit hard to follow. Some textual description of the three ingredients and their relation to parameters and specific implementation would be helpful here (e.g. inheriting phase-values = initial condition). The authors do a better job in the supplementary text at describing this, so integrating some of this text into the main manuscript could be helpful. More generally, being careful to have a consistent terminology (e.g. 'gradient', PG, k3/4) throughout the text will help aid the transition to the explant explorations.

We thank the reviewer for their comment and we have now adjusted the text and figures accordingly to make the transition from tail simulations to explants (lines 574-581), as well as the transition from dynamical systems theory to tail simulations, much easier to follow and understand. We are now consistent in our naming of the three main components of the models, and we have added labels to these three components in Figure 4D and Figure 5G:

- Phase values (u and v)
- Regions (k1)
- Frequency gradient FG (k3/k4)

2. Some annotation of the simulation 'domain' in Fig 4 could aid understanding. This would include some diagrammatic description of (a) where R and PG are, and their relation to k1/3/4; (b) how boundary conditions are implemented; (c) how growth is implemented (e.g. are posterior cells duplicated, vs 'blank' cells added).

As mentioned above, we completely agree with the reviewer that including a better description of the components of the models and how they work together has improved the readability of the manuscript.

(a) In Figure 4D we have added labels of the names, abbreviations and variables used in the simulation of the Clock and Wavefront (CW) model according to the list written in the previous comment above. In addition, in Tables 1-7 in the Methods Section we have added all parameters used for all simulations where the readers can find all the details of initial phase values of u and v, and the R and FG used, including values of k1/k3/k4 and slopes of the transitions between the values, to improve the reproducibility of the paper.

(b) In our understanding this is a two-fold subject. First, the global boundary conditions of the tail which are zero-flux which are equal for all models (Methods Section "Boundary conditions"). Second, the transition areas between sections of different behaviors within individual tails. These are shown by the shapes of the profiles of R and FG presented in Figure 4, in combination with the detailed description of the parameters used in Tables 1-7. We have also added the phase spaces that correspond to the different behaviors to each model (Figure 4E,H,K) to better illustrate how R changes the phase space along the anterior-posterior axis.

(c) In Figure 4D we have also added an illustration of how growth is included in the model, where a copy of the most posterior file is added to the posterior side, while R and FG are shifted posteriorly to follow along with the growth of the tail.

3. Lines 582-590: in CWS, Wnt and Notch are out of phase in the posterior and in-phase in the middle. Can you elaborate on why this occurs: to me this is not an intuitive result, and given interest in phase-difference-gradients, this could be an important point for the uptake of this

© 2024. Published by The Company of Biologists under the terms of the Creative Commons Attribution License (https://creativecommons.org/licenses/by/4.0/).

theoretical work in subsequent experimental studies.

We thank the reviewer for bringing up this interesting question that indeed is not at all intuitive and requires many technical details to be explained properly. That is why we had taken it out of the manuscript due to space constraints, but we are more than happy to discuss our explanation, which we now have included in supplementary Figure S24 with a brief discussion in lines 462-469. Wnt and Notch switch from having phase-shifted oscillations in the posterior region to being in-phase in the excitable regime. This change stems from changes in the shape of the limit cycles for k1=1 and k1=2.3.

In Figure S24 we show that the oscillatory behavior of the most posterior tail part in both the CG and CWS models, corresponding to the case with k1=1, exhibits a limit cycle with an approximate tilted square shape, see Figure S24A,C. As cells oscillate around this tilted square, there are instances where Wnt is high and Notch is low (upper left corner), and vice versa (lower right corner), resulting in a phase-shift of Wnt and Notch oscillations in the posterior region. Such phase shifts are common in oscillatory models characterized by delayed negative feedback between reactants (Novák and Tyson 2008, https://doi.org/10.1038/nrm2530). While the specific shape of the limit cycle may vary depending on the model and its parameters, the phase shift is an inherent property of delayed negative feedback, which is essential for sustaining oscillations. This principle is also illustrated in the minimal two-reactant model presented in Figure 1 of Casani-Galdon and Garcia-Ojalvo 2022 (https://doi.org/10.1016/j.ceb.2022.102130), where a phase shift between the reactants is observed with a minimal delay (tau) that supports sustained oscillations. In essence, oscillations with a phase shift (out of phase) are common in biochemical oscillators. Crucially, modulating the frequency of these oscillations, as promoted along the anterior-posterior axis in the CG model, leaves the shape of the limit cycle unchanged and does not alter the phase shift between reactants (Figure S24C-D).

In contrast, the limit cycle underlying phase waves in the intermediate part of the tail of the CWS model emerges from a diffusion-driven destabilization of the two stable states at k=2.3. Within this region, neighboring cells in opposite states destabilize each other repeatedly via diffusion, forming a narrow limit cycle along the diagonal from low to high Wnt and Notch values (Figure S24B). This promotes a change from phase shifted oscillations at the posterior side of the tail to in-phase oscillation in the middle part of the tail. Furthermore, as cells move through this new limit cycle they spend the majority of the time around the stable states, where Wnt and Notch are in phase, further reducing the phase shift between these two signalings. Notably, this occurs only when Wnt promotes Notch and Notch inhibits Wnt; otherwise, stable states are inverted causing out-of-phase oscillations (Figure S23). The biological support for these interactions is discussed in lines 449-461.

4. Line 463-5: the wording here is quite unclear. Can this be recast in simpler terms to provide some intuition. The numerical experiments suggested above may bolster what I think the authors are trying to argue here as well.

We agree with the reviewer that this paragraph was difficult to understand so we have re-written it to make it more clear that what we want to say is that the oscillations in the posterior tip of the tail initiate the excitable waves (lines 387-395).

Additional (non-essential) extensions that may aid the argument

----

1. In Movie 8 and 9, the gradient of k1 is not scaled to embryo size. This may indeed be reasonable, however there have been some suggestions of a scaled gradient (10.1242/dev.161257). How does scaling the entire gradient, or e.g. the oscillatory-to-excitable part affect the results?

We thank the reviewer for bringing our attention to this very interesting paper. In Ishimatsu et al. 2018 (https://doi.org/10.1242/dev.161257) they present a Clock and Scaled Gradient model where amongst many results is that the somite specification point moves faster posteriorly than the rate of the posterior growth, so effectively the gradient scales over time to create a smaller and smaller oscillatory region. Furthermore, they find that there is a 4-cycle delay between the

© 2024. Published by The Company of Biologists under the terms of the Creative Commons Attribution License (https://creativecommons.org/licenses/by/4.0/).

state of the tail at the somite specification time and when the somite is formed in Zebrafish (the 'echo effect').

In general, to show how the CWS model works for different scalings of the size of the oscillatory part of the tail, we have added Figure S19 where we see that the overall patterning behavior of the CWS model is not affected by having a small or large size of the oscillatory region relative to the excitable region. In order to replicate the results presented in Ishimatsu et al. 2018 with our implementation of the CG model, as well as the CWS model, it would be very interesting to study how dynamical scaling of the regions (R: k1) and the frequency gradient (FG: k3/k4) during the simulations would affect the patterning. This goes beyond the scope of this manuscript, but would be possible to implement in the models by decoupling the rate of the growth of the tail with the progression of the regions (R) and frequency gradient (FG), making R and FG move posteriorly slightly faster than the tail grows.

2. If I understand the work correctly, the transition from oscillation/excitable phase-waves (CG vs CWS) to somite formation is due to change in bifurcation parameter (aka the wavefront). The spatial (and temporal) position of the bifurcation then locks in cell fate/establishes the somites. Does the speed of the progression of the (k1>bistablity bifurcation) vs speed of oscillations affect somite size/regularity of size? A phase diagram tuning each of these could be useful. Commenting on this in relation to doi:10.1126/science.1253089 could be helpful.

That is exactly correct. We now show that in agreement with data, tuning the speed of the progression of the bifurcation (from excitability to bistability) and frequency of oscillations in the posterior side affects the somites in the CWS model in the following way:

- Slower progression of the bifurcation, implemented as slower growth of the tail gives compressed somites as shown in Goudevenou et al. 2011 (https://doi.org/10.1371/journal.pone.0026548)
- Slower oscillations in the posterior side gives larger somites as shown in Herrgen et al. 2010 (https://doi.org/10.1016/j.cub.2010.06.034)

We present these new simulations in Figure S25. The same perturbations in the Sevilletor implementation of the CG model will give equivalent results.

In Soroldoni et al. 2014 (https://doi.org/10.1126/science.1253089) they present a somitogenesis model with a Doppler effect with tissue shortening, i.e where the length of the posterior part of the tail reduces over time (similar to the Clock and Scaled Gradient model by Ishimatsu et al. 2018 discussed above). This is related to the perturbation suggested above (decoupling the progression of R and FG with the growth of the tail) and would definitely be interesting to do in future projects in order to further test the models up against experimental findings.

3. In figure S5, diffusion is shown to alter the spatial profile of waves in the unbounded case. How does the choice of diffusion coefficient influence (a) the spatial and (b) the temporal profile of phase waves in general, and in the 'in-silico PSM' (where the excitable regime is bounded by different regimes on each side).

In the unbounded case with phase waves shown in Figure S10 (in the current manuscript, previously Figure S5) we see that increasing D for a given value of k1 increases the wavelength of the pattern and it can also reduce the "length of the spiral arms". This is also shown in the new Figure S11A where we vary dx and D. In tail simulations, we now show that when we vary only the parameter D in the CWS model in Figure S22A, there is an equivalent patterning behavior with an increased value of D. We believe this is due to the boundaries of the excitable regions, where the waves are guided by the pacemaker on the posterior side. The period of the posterior oscillations are not changed by diffusion, so the waves that propagate in the excitable region will not be significantly different from the case with standard amount of diffusion. However, when the diffusion is too low to excite the cells in the middle section then no phase waves are formed, and the CWS model effectively acts as the Clock and Wavefront (CW) model (Figure S22A). In addition, we found that adding diffusion of v to the model did not disrupt the dynamics (Figure S22B)

As discussed above, in Figure S12 we performed simulations of the tails with re-scaled values of both D and dx, which showed equivalent results for the three models (PORD/CG/CWS) when the

© 2024. Published by The Company of Biologists under the terms of the Creative Commons Attribution License (https://creativecommons.org/licenses/by/4.0/).

ratio between the D and dx is kept constant at sqrt(D)/dx (Supplementary Section S8 and lines 417-421 in the Manuscript). This shows that D can be decreased in the CWS model, but only when the sizes of the cells are also decreased correspondingly.

4. When scrambling initial state (dissociated and re-aggregated explants), only in CWS do circular wave patterns emerge. Ultimately, this can be explained alternatively by internal establishment of long-range gradients (which could even be a purely passive process, given the spatial profile of an upstream diffusible signal that controls k3/4, e.g. FGF, as could be expected from uniform production, diffusion, and leakage of the morphogen out of the perimeter of the circular explant).

We thank the reviewer for pointing out that the previous version of the manuscript would be interpreted to say that only the CWS model forms circular waves in mixed explants. In the current version of the manuscript we make it clear that both the CG and the CWS model form rotating wave patterns from a scrambled initial state (lines 602-610, Figure S28 and Movie 13). In both cases, the waves arise from rotating centers during the synchronization of phases from the random initial phases. In this case, a re-established gradient that controls the phases is not needed to explain the emergence of the dynamical pattern formation in the CG explant. In lines 611-618 we also discuss how this pattern formation could be further investigated experimentally.

Generally it is true that graded signals in the tail such as FGF can be re-established in the explants, so we now mention that the gradient FG (k3/k4) could be re-established in the explants (lines 576-581 and Supplementary Section S20) and we have included this option for the PORD and CG explants in Figure 5H,J to the left. This new option replaces the previous simulations with an “inherited FG” (previous version of the manuscript: Figure 5H,I top rows), which had some noise due to shuffling during the creation of the explants. The new explant simulation with asmooth re-established circular gradient FG without noise can create a target pattern in the CG model in agreement with experiments (current version of the manuscript: Figure 5J to the left).

We would like to thank Reviewer 1 for the time and effort that went into considering all the ways we could strengthen and improve the manuscript and provide the readers with as much information and valuable details as possible. We are very happy to have added simulations according to their suggestions and we believe that it has significantly improved our manuscript.

***** Reviewer 2 Advance Summary and Potential Significance to Field:
In their manuscript “A Clock and Wavefront Self-Organizing Model Recreates theDynamics of Mouse Somitogenesis in-vivo and in-vitro”, Klepstad and Marcon present a new modelling framework for self-organizing dynamic processes in tissues. It is based on the combination of a reaction-diffusion system with negative feedback and longer range gradients, which they term the Sevillator. After an initial characterization and parameter testing, the model is used to investigate the process of somitogenesis, using experimental data from mouse embryos as baseline. This model can recapitulate certain experimental findings that other models could not, such as the clock and gradient (CG) model and progressive oscillatory reaction diffusion (PORD) model. This is a very well written manuscript that brings together several recent experimental findings in a new model and poses very interesting predictions that can be tested in future experiments.

Reviewer 2 Comments for the Author:
I have read the manuscript with a main focus on the biology and will not judge the mathematical implementation of the model, even though it makes sense to me.

There are a few points that should be clarified on the one hand or added to increase the usefulness for developmental biologists on the other hand.

We greatly appreciate the positive and constructive feedback provided by the reviewer. Recognizing the importance of discussing the biological relevance of our predictions, we have rephrased and expanded the manuscript.

© 2024. Published by The Company of Biologists under the terms of the Creative Commons Attribution License (https://creativecommons.org/licenses/by/4.0/).

Specifically, in response to the reviewer's comments, we included a detailed discussion of the biological studies that support the interaction between Wnt and Notch in the model. This is presented in the section about “molecular implementations of the model” and the section about “the excitability of the CWS model”. Here we also discuss the effect of the Yap pathway on the oscillation of mouse PSM cells in-vitro and its relevance within the CWS model.

This extensive exploration has led us to propose two novel testable predictions. One prediction pertains to the effect of Wnt signaling perturbation in isolated PSM cells in-vitro, which we hypothesize could recover the oscillatory behavior of cells grown in stiff substrate compensating for the effect of Yap. The other prediction is that neighboring PSM cells should oscillate out of phase in-vitro, due to the specific excitable behavior of the CWS model. In support of this prediction we have included a novel preliminary quantification of the data presented in Hubaud et al. 2017 (https://doi.org/10.1016/j.cell.2017.08.043), now featured in Figure S32.

Furthermore, we emphasized that our study's primary objective is not to favor one model over another, but rather to demonstrate how the Sevilletor equations can be used to explore the patterning capacity of different models. As now mentioned in the discussion section, the patterning regimes of different models are not necessarily mutually exclusive (lines 620-630). Within this context, we have also clarified that the main usefulness of the CWS model is its ability to recapitulate the excitability of the mouse PSM by explicitly considering the effect of cell communications and the coordination between Notch and Wnt signaling observed in mouse.

- Several times throughout the manuscript, the authors present predictions of their model, some of which do not fit to available experimental data. The authors should follow up upon these further. These have two-fold relevance:

On the one hand, the authors can speculate what their findings could mean for the biological process of somitogenesis.

Their implementation of the PORD model does not recapitulate certain experimental findings from the literature, such as the occurrence of multiple phase waves in beta catenin dominant active mice. In contrast, their new CWS model can.

Another example is the experimental finding that the phase-relationship between Wnt and Notch changes from out-of-phase to in-phase along the anterior posterior axis.

It would be interesting to discuss what we can learn from these differences.

We thank the reviewer for prompting us to present a more general discussion of the “take home messages” of our study. As we now mention in the Results and Discussion (lines 356-362 and 660-672), the key difference between the PORD, CG and the CWS model lies in how phase waves emerge. In the PORD and CG model, phase waves arise due to a frequency modulation within a pure oscillatory state, while in the CWS model they arise from the excitation of a bi-stable state. Additionally in the PORD models the oscillations stop due to a self-organizing lateral inhibition mechanism, while in the CG and CWS model they arrest due to a wavefront. We have improved the overall manuscript to clarify these differences and modified Figure 4.

As the reviewer suggests, the different patterning mechanisms of the model underlie different biological predictions. Following the comments of the other reviewers, we are now more cautious about the prediction regarding the formation of multiple phase waves observed upon stabilization of beta catenin shown in Aulehla et al. 2008 (https://doi.org/10.1038/ncb1679). This is now discussed in the supplementary Figure S21. Here, the most striking qualitative difference between the patterning behavior of the CG and the CWS model is the case without a frequency gradient, where the CG model forms no waves (Figure S21J) and the CWS can form multiple waves (Figure S21F). However, we now show the behavior of the CG model for a wider set of conditions than in the previous version of the manuscript, which could also relate to the beta catenin gain of function shown in Aulehla et al. 2008.

Additionally, we examine the response of the models to comparable noise levels applied to parameters and oscillation phase (Figure 4G,J,M), and upon cell position switching (Figure S17).

© 2024. Published by The Company of Biologists under the terms of the Creative Commons Attribution License (https://creativecommons.org/licenses/by/4.0/).

This analysis reveals that our PORD model implementation exhibits greater fragility to noise than the CG and CWS models due to underlying lateral inhibition patterning. Importantly, this fragility is evident also in the original PORD model (Figure S14) due to the same underlying lateral inhibition patterning regime, as discussed in Supplementary Section S9.

As highlighted by the reviewer, our analysis also brings predictions regarding the phase relationship between Notch and Wnt oscillations in the CG and CWS models. Specifically, in the posterior tail region of both models, Wnt and Notch oscillate with a phase shift due to the characteristic shape of the limit cycle in phase space, as discussed now in Figure S24A,C. This limit cycle shape is typical of oscillatory models featuring negative feedback between multiple reactants (Novák and Tyson 2008, https://doi.org/10.1038/nrm2530, Casani-Galdon and Garcia-Ojalvo 2022, https://doi.org/10.1016/j.ceb.2022.102130). Our theoretical examination reveals that decreasing the frequency within this oscillatory regime does not alter the limit cycle's shape, as shown in Figure S24C-D. Consequently, frequency variations along the anterior-posterior axis, like those in classical CG models, do not affect the phase shift between Wnt and Notch oscillations. On the contrary, the presence of the intermediate bi-stable excitable regime in the CWS model can naturally predict a switch into in-phase oscillation of Wnt and Notch, as observed in the mouse tail (Sonnen et al. 2018, https://doi.org/10.1016/j.cell.2018.01.026). We observe that this phenomenon occurs when neighboring cells excite each other from the steady state where Wnt and Notch are both low or high, generating a new limit cycle where Wnt and Notch oscillate in-phase, Figure S24B. Importantly, we learn that this is possible only when Wnt promotes Notch and Notch inhibits Wnt, as inverting the interactions would also invert the stable states of the system driving out of phase oscillations, see Figure S23. Addressing the comments raised by the reviewer below, we also discuss how these interactions are consistent with the excitable behavior observed in isolated cells of the mouse PSM presented in Hubaud et al. 2017 (https://doi.org/10.1016/j.cell.2017.08.043).

Finally, given that neighboring cells can excite each other when they are in opposite positions in the limit cycle, we added a novel prediction of the CWS model, which is that neighboring cells should have a tendency to maintain out of phase oscillation between them (lines 538-546 and Figure S32). Importantly, this prediction should not be confused with the prediction about the change in relative phase between Notch and Wnt oscillations. In this case, we are considering that all cells are in the excitable regime where Wnt and Notch oscillate in phase. The out of phase oscillation among neighboring cells refers to the fact that when one cell is in a high phase of Wnt and Notch, the neighboring cells in a low phase for both Wnt and Notch. We now show a preliminary analysis of the in-vitro data presented in Habaud 2017, showing that neighboring cells indeed exhibit out of phase oscillations of the LuVeLu reporter as predicted by the model (Figure S32).

On the other hand, their model provides very concrete predictions that should be tested experimentally in the future.

One example is the finding that Wnt has to have a positive effect on Notch and Notch a negative effect on Wnt to allow the change in Wnt Notch phase-relationship along the tissue. The authors could discuss these predictions more explicitly and make suggestions on what should be tested to support the validity of their model and this way understand the segmentation clock better.

Theoretically, the prediction stems from the dynamics of the two variations in the phase space. We have added panels D and H to Figure S23 to illustrate these differences. It is know from experimental findings by Sonnen et al. 2018 (https://doi.org/10.1016/j.cell.2018.01.026) and Aulehla et al. 2003 (https://doi.org/10.1016/S1534-5807(03)00055-8) that Wnt and Notch oscillate out of phase in the most posterior part of the tail, and synchronize to become in-phase in the anterior side. When Wnt has a positive effect on Notch and Notch a negative effect on Wnt, the shape of the nullclines create steady states in the upper right and lower left corners of the phase space, where both Wnt and Notch are either high or low (Figure S23D). The oscillations in this phase space creates phase waves that are in-phase (described in detail in Figure S24) and a wave pattern that arrests anteriorly in-phase. For the opposite case, where Notch has a positive effect on Wnt and Wnt a negative effect on Notch, the phase space is the mirror image of the standard (Figure S23H). In other words, the nullclines have been flipped from left to right. This

© 2024. Published by The Company of Biologists under the terms of the Creative Commons Attribution License (https://creativecommons.org/licenses/by/4.0/).

creates phase waves and arrested oscillations around the steady states where Notch is high and Wnt is low (upper left) and where Notch is low and Wnt is high (lower right).

From an experimental perspective, in lines 462-473 we discuss the evidence supporting interactions between Wnt and Notch within the segmentation clock. Specifically, we propose that Notch may inhibit Wnt, as discussed in Acar et al. 2021 (https://doi.org/10.1038/s41598-021-88618-5), and suggest that the crosstalk between these pathways may be further mediated by a positive interaction between Wnt and Notch, as proposed in Aulehla et al. 2003 (https://doi.org/10.1016/s1534-5807(03)00055-8) and Gibb et al. 2009 (https://doi.org/10.1016%2Fj.ydbio.2009.02.035). Additionally, we clarify that the negative feedback between Wnt and Notch may act redundantly to the more established self-regulatory negative feedback of Notch (lines 454-461, Figure 4N-P).

Our hypothesis is that crosstalk between Wnt and Notch also mediates the excitable behavior of mouse PSM cells. We demonstrate that this hypothesis aligns with the effect of Notch inhibition applied in high-density cultures, as illustrated in Figure 5C-E. Furthermore, our model suggests that one way in which YAP signaling could reduce the excitability of PSM cells, as observed in Hubaud et al. 2017 (https://doi.org/10.1016/j.cell.2017.08.043), is by altering the self-regulation strength of Wnt, mediated by cross-talk between YAP and Wnt signaling (Jiang et al. 2020, https://doi.org/10.3892/mmr.2020.11529). In lines 526-537, we propose that this could be tested by partially inhibiting Wnt signaling in high-density cells grown in Fibronectin to mitigate the effect of YAP and restore oscillations.

- The authors use explants as experimental conditions to compare their new model to the PORD and CG model. In their analysis, the authors should keep in mind that gradients do reform during 2D culture which correlates with formation of phase waves (Lauschke et al.). Only in complex culture conditions with Wnt and FGF activation and BMP inhibition, oscillation dynamics of the explant or reaggregates are changed (Hubaud et al.). However, in these latter conditions also endogenous signalling gradients are changed. In their data (Figure 5), the authors consider all possibilities. However, the discussion of the biology and interpretation of their results should be adapted.

In general, in all their interpretation of data from Hubaud et al., the authors should consider that experiments were performed in “explant medium (dissection medium with 0.1mM of β-mercaptoethanol, Chir-99021 (Sigma) 3 μM, LDN-193189 (Sigma/Stemgent) 200nM, BMS-493 (Sigma) 2.5 μM, mFGF4 (R&D) 50ng/mL, heparin (Sigma) 1 μg/mL and Y-27632 (P212121/Tocris/Selleckchem) 10 μM)”, while in Lauschke et al. explants were cultured DMEM F12 without any small molecules.

Indeed we agree that these are important aspects to keep in mind while comparing experimental findings to simulations, so we thank the reviewer for their comment. In the current version of the manuscript we have improved the presentation of the experiments by Lauschke et al. 2013 (https://doi.org/10.1038/nature11804) and Hubaud et al. 2017 (https://doi.org/10.1016/j.cell.2017.08.043) in the Results Section (lines 553-568) and we provide a more detailed description of the experimental procedures used in Lauschke et al. 2013 and Hubaud et al. 2017 in Supplementary Section S20.

Specifically, as pointed out by the reviewer, we acknowledge that depending on the experimental protocol used, phase waves either ceased after a few cycles with a re-establishment of global signaling gradients (Lauschke et al., 2013), or persisted over two days in the absence of Fgf signaling gradients (Hubaud et al., 2017). Since our study primarily focuses on phase wave formation, we specifically consider only the initial oscillatory dynamics observed by Lauschke et al. (2013) and the oscillations in Hubaud et al., 2017. Additionally, given the uncertainty regarding the role that re-established gradients may play in both situations, we clarify that our simulations aim to explore more generally how well different models can synchronize phase waves with decreasing levels of guiding cues in explants. In response to suggestions from other reviewers, we have enhanced Figure 5G-M to illustrate this approach more clearly.

From these simulations, we learn that PORD explants fail to produce phase waves due to the fragility of the model in response to cell rearrangements. On the other hand, both the CG and

© 2024. Published by The Company of Biologists under the terms of the Creative Commons Attribution License (https://creativecommons.org/licenses/by/4.0/).

CWS models are capable of generating circular phase waves even without gradients. In this scenario, the models primarily rely on initial phase values inherited from the tail. Additionally, the CWS model can generate phase waves even in the absence of initial phase values, which is influenced by cells from the tip of the tail serving as a central pacemaker population. Consistent with experiments outlined in Hubaud et al. (2017), we demonstrate in Figure S29 that the circular waves can persist in CWS explants after the ablation of the central population. Moreover, similarly results are observed in explants lacking cells from the tip (Figure 5M).

The key message is that both CG and CWS explants can effectively generate self-organizing phase waves solely guided from a pre-pattern of phase values. However, the CWS model can additionally generate circular phase waves from homogeneous initial phase values. Furthermore, we now clarify (see answers below) and in lines 602-610 and Figure S28 and Movie 13 that both models perform similarly well in mixed explants, generating rotating patterns. Additionally, the CWS model can generate periodic phase waves.

- The authors should be careful when interpreting experimental findings that inhibition of Notch signalling “arrests oscillations” (line 635, Figure 5C). The reporter Luvelu is not only a reporter for oscillations but also a direct target gene of Notch signalling. However, in the framework of their interpretation their data makes sense.

We agree with the reviewer that the sentence needed to be rephrased, as it was not our intention to say that Notch arrests oscillations in general. To make this clear now say:

“A previous study showed that mouse PSM cells stop Notch’s target oscillations like Lnfg at low density in-vitro cultures on Fibronectin, but can be excited to oscillate when the density is increased” (lines 488-491)

“Previous experiments proposed that inhibiting Notch signaling reduces the stimulus that triggers excitability, leading to the arrest of oscillations observed in the LuVeLu reporter.” (lines 508-510)

- There are a few aspects mentioned in the introduction that should be rewritten or added. These mostly do not have an impact on the model or the results section, but should be changed.

- Line 45: Add Takashima et. al PNAS 2011 as reference for delayed feedback.

  We agree with the addition of the reference which we have added (lines 49-50).

- Line 46 and following: “The specific mechanism responsible for the spatial synchronization of oscillations, however, remains a subject of debate.” Is not entirely correct, as there is evidence that Notch signalling is responsible for the synchronization between cells. I think the authors mean the tissue-wide pattern. That should be rewritten to make clear.

  We have changed text accordingly to “The specific mechanism responsible for the spatial synchronization of oscillations at the tissue level, however, remains a subject of debate” (lines 68-70).

- In addition, references should be added to the two possibilities mentioned in lines 48 and 50.

  We have added a reference to Kuyyamudi et al. 2022 (https://doi.org/10.1088/1478-3975/ac31a3) with a specific example of how a global gradient has been thought to control the frequency to the first statement: “One possibility is that the tissue-wide frequency profile is regulated by posterior signals, for example by modulating the local coupling-strength of delta-notch” which is now in lines 99-102 in the current manuscript.

  The second statement: “Another possibility is that the oscillations are synchronized through local cell communication” in line 50 in the previous version of the manuscript

© 2024. Published by The Company of Biologists under the terms of the Creative Commons Attribution License (https://creativecommons.org/licenses/by/4.0/).

has been removed due to space constraints, but is otherwise discussed in lines 111-114: “On the other hand, the synchronization of oscillation underlying phase waves may involve local cell communication.” where we refer to: Maroto et al. 2005 (https://doi.org/10.1387/ijdb.041958mm), Masamizu et al. 2006 (https://doi.org/10.1073/pnas.0508658103), Webb et al. 2016 (https://doi.org/10.7554/eLife.08438), and Oates et al. 2020 (https://doi.org/10.1016/j.gde.2020.04.008).

- Line 80 etc.: “Moreover, experimental evidence shows that posterior signaling gradients of Wnt and Fgf allow cells to oscillate at the posterior tip of the tail.” Is a too strong statement. There is indeed a correlation between gradients and oscillations. Even though there is experimental evidence that gradients influence the waves, as the authors also discuss in the paper, how exactly they are linked and whether the gradients are necessary for the oscillations still has to be investigated. The authors should rephrase it.

  This is true and we thank the reviewer for pointing out this statement so we could rephrase it to “Moreover, experimental evidence shows that posterior signaling gradients of Wnt and Fgf can *modulate* the oscillations at the posterior tip of the tail (Dubrulle et al., 2001; Naiche et al., 2011; Aulehla and Pourquié, 2010)” (lines 83-86).

- Lines 123 and following: it has been shown in both explant cultures and reaggregates that signalling gradients are re-established (Lauschke et al. 2013, Tsiairis et al. 2015). In fact, in reaggregates this correlates with the transition from in-phase oscillations to waves. I agree with the authors’ conclusion that PSM cells have the capacitiy to self-organize, however this cannot be separated from the spatial gradients. This aspect also has implications for their modelling data here.

  When discussing results of the explant simulations we now make it clear that explant simulations reflect only the broad, high-level qualitative behavior of the models rather than quantitative details. We show virtual explants with a variety of guiding cues that can either be inherited from the tail, or re-established by the cells ex-vivo (lines 570-581). As mentioned above, we do not directly compare the simulations with experimental findings, but discuss how the properties of the models could relate to experimental setups. In the Results Section (lines 611-618) we discuss what future experiments could take into account to explore the possible self-organizing aspects of the mixed explants. We also have a more detailed description of the experimental procedures used in Lauschke et al. 2013 (https://doi.org/10.1038/nature11804) and Hubaud et al. 2017 (http://dx.doi.org/10.1016/j.cell.2017.08.043) in Supplementary Section S20.

  We have also double-checked that it is clear, as this was the intention in our previous version of the manuscript as well, that the majority of explant simulations of the CG model are also able to recapitulate experimental data. As an improvement to the paper, after comments from the reviewers, we have replaced the “inherited gradients” in explants (previous version of the manuscript: Figure 5H,I top rows), which had some noise due to shuffling during the creation of the explants, with a new explant simulation with a smooth re-established circular gradient by the function FG without noise (current version of the manuscript: Figure 5H,J to the left). In these explants, a target pattern can be formed in the CG model in agreement with experiments.

- Besides changes in the phase shift between Wnt and Notch signalling, there is also an increase in oscillation amplitude from posterior to anterior. It would be interesting to investigate how amplitude changes along the tissue depending on AP position. However, this might be beyond the scope of this study, which I cannot judge properly.

This is a very interesting point that would be great to explore in future projects. In Figure 4F,I,L we have added the average values of u and v along the x-axis for each model at the end of the simulations, where it can be seen that all three models have a small increase in amplitude as the oscillations freeze, but it is not a significant change. We don’t exclude, however, that the amplitude difference could be larger with other parameters.

© 2024. Published by The Company of Biologists under the terms of the Creative Commons Attribution License (https://creativecommons.org/licenses/by/4.0/).

Minor:
- In lines 818 following the authors discuss oscillations in single cells. In this discussion, the authors should also mention the possible influence of cellular mechanics on signalling oscillations. As the authors write, when single mouse cells are cultured on BSA, they oscillate, which cells don't do when adherent.

We now discuss that the difference in behavior upon changes in cell density were observed in Fibronectin (lines 488-491) (Hubaud et al. 2017, http://dx.doi.org/10.1016/j.cell.2017.08.043). We also discuss that isolated cells can be recovered to oscillate by reducing YAP signaling in lines 518-537. We now speculate that the effect of YAP signaling in the CWS model could be mediated by changes in the relative strength of the auto-regulation of Wnt and Notch (specifically Wnt). As mentioned in lines 529-534, this idea is also consistent with the observation that fibronectin increases from posterior to anterior in the chick PSM, which in turn could increase YAP, changing the rate of Wnt auto-activation (k1) as shown in our CWS model with a graded increase in k1 along the anterior-posterior axis in Figure 4Q.

- The authors discuss that there Sevillator can be used to model other aspects in biology/ developmental biology. It would be useful if the authors could expand on this further and give examples.

This is a great suggestion that we followed to rewrite the last paragraph of the Discussion (lines 712-727). Here we discuss how the Sevilletor framework can be used to study other early development patterning processes in mouse that involve Notch where lateral inhibition chessboard patterns are created, such as in the retina (Formosa-Jordan et al. 2013, https://doi.org/10.1387/ijdb.120259jf) and inner ear (Adam et al. 1998, https://doi.org/10.1242/dev.125.23.4645 and Petrovic et al. 2014, https://doi.org/10.1242/dev.108100).

- Fig. 2C: Symbols are too small to discern properly. The same is true for Fig. 4B, where also the legend is missing.
- Fig. 5G-N: It might be useful to restructure this whole part of the figure to indicate, what the input conditions (Fig.5D) and the results of each model were (Fig. 5 H-J). Right now, the figure is difficult to read, in my opinion.

We agree with the reviewer that the last part of Figure 5 was difficult to read, so we have re-structured Figure 5 and also made sure that all text and captions in the figures are easily readable.

We sincerely thank Reviewer 2 for their focused discussion of the biological aspects of the manuscript which have guided us to increase the usefulness of the paper for developmental biologists. It is now easier to understand what we can learn from the models and how the predictions relate to experiments, which has improved the manuscripts' scientific impact.

***** Reviewer 3 Advance Summary and Potential Significance to Field:
The manuscript "A Clock and Wavefront Self-Organizing Model Recreates the Dynamics of Mouse Somitogenesis in-vivo and in-vitro" by Klepstad and Marcon presents a minimal dynamical system model, based on two coupled partial differential equations, that they have termed the Sevilletor. The model is remarkable, because tuning very few parameters it is able to produce very different dynamical regimes relevant for segmentation clock studies: fixed points, bistability, relaxation and excitable oscillations. This is the beauty and the strength of this model: it allows to very cleanly transition between this different dynamical regimes, and spatial variations of the model parameters can produce a wealth of dynamics reminiscent of observations in different settings of segmentation clock studies. I therefore think that this setting can be a powerful tool to gain understanding on the underlying mechanisms of segmentation clock dynamics in different organisms and settings, and therefore recommend publication.

However, I have two main comments about the origin of the model and its relation with previous research, and the tone that the manuscript has in some paragraphs.

© 2024. Published by The Company of Biologists under the terms of the Creative Commons Attribution License (https://creativecommons.org/licenses/by/4.0/).

We are very grateful to the reviewer for recognizing the value of our approach to study the field of somitogenesis. We are happy to have addressed all the comments about improving our discussion of dynamical system theory as well as previous somitogenesis models, and we believe that this has helped us to significantly improve the overall tone of the manuscript and write a better balanced discussion of our findings.

Reviewer 3 Comments for the Author:
Eqs. 1-2 are very closely related to the complex Ginzburg-Landau equation (CGLE) with broken gauge invariance, see Aranson and Kramer for a review of the state of the art in 2002. The CGLE with broken gauge invariance is in general of an excitable nature, and it shows transitions from oscillations to bistability (actually, varying the parameter gamma in Eq. 86 of Aranson and Kramer is quite equivalent to varying the difference between k1 and k2 in the current manuscript). It has very rich dynamics, and I would be utterly surprised if the dynamical behaviors described in the manuscript have not been observed or described in the CGLE framework, for which an extensive literature exists.

We agree with the reviewer's observation that in the previous version of the manuscript, we did not adequately emphasize how other dynamical systems, such as the complex Ginzburg-Landau equation (CGLE), can exhibit similar behaviors and bifurcations. Following the reviewer's insightful comment, we realized that in our effort to condense the manuscript, we removed important citations to previous theoretical work in the main manuscript. We have rectified this by including references to fundamental studies in the Introduction in lines 145-149, including the influential work of Tyson and Keener 1988 mentioned by the reviewer. Importantly, in the Results section in lines 195-202 we now clarify that while previous models, like the CGLE, Fitzhugh-Nagumo, or Oregonator can undergo similar bifurcations from an oscillatory to a bi-stable state, their bi-stable regime typically features a central unstable steady state and two stable steady states. In contrast, the Sevilletor model exhibits a bi-stable regime characterized by 5 fixed points: a central unstable point and two stable points situated near unstable points. The presence of an unstable point in the proximity of each stable point is crucial for the Sevilletor's diffusion-driven excitable behavior, distinguishing it from the excitability observed in the CGLE, Fitzhugh-Nagumo, or Oregonator model (lines 221-239), as elaborated below and in the supplementary Section S3 with Figure S3 and Figure S4. We also acknowledge that similar bifurcations and phase portraits have been observed in Jutras-Dube et al. 2020 (https://doi.org/10.7554/eLife.55778) (Francois et al. 2007 https://doi.org/10.1038/msb4100192) (lines 228-230), although these studies did not explore the effect of diffusion and excitable behaviors.

This connection should be made in the manuscript, and, importantly, I am convinced that assertions like that of line 239 (“This excitable self-organizing patterning behavior has not been described previously”) do not hold.

Thanks to the reviewer’s comment, we realize that this sentence lacked details and could be misinterpreted. As we said in the previous point, we agree with the reviewer that there are several other models that exhibit excitable behaviors and bi-stablity. We now emphasize that the Sevilletor equations exhibit a form of excitability that to the best of our knowledge has not been characterized before. This excitability is based on a diffusion-driven behavior mediated by a complex phase portrait with 5 fixed points (see above). As we now explain in the supplementary Section S3, diffusion between neighboring cells that are in opposite bi-stable states can repeatedly push the cells towards the trajectory of the nearby unstable point generating a limit cycle. In supplementary Section S3, we delineate the distinction between this diffusion-driven excitable behavior and the excitable behavior seen in previous models from a single stable steady state, or wavefront formation in bi-stable regimes.

In agreement with this explanation, we have rephrased the sentence mentioned by the reviewer as follows “near this bifurcation, our system exhibits a previously undescribed dynamic behavior: the formation of periodic phase waves via diffusion-driven excitation of a bistable state” in lines 151-154 in the Introduction. Throughout the manuscript wemake sure that it is clear that the Sevilletor excitability behavior involves two stable as well as three unstable points. In the Results section lines 500-503 and Discussion section lines 673-681, we also clarified that this diffusion-driven excitable behavior differs from the excitable behavior of the FitzHugh Nagumo equations

© 2024. Published by The Company of Biologists under the terms of the Creative Commons Attribution License (https://creativecommons.org/licenses/by/4.0/).

used in Hubaud et al. 2017 (http://dx.doi.org/10.1016/j.cell.2017.08.043).

Moreover, pattern formation in excitable media has been a very intensive field of research at least since the 80s, where all kinds of imaginable phenomena have been observed. Already in 1988 Tyson and Keener wrote a review (with 999 citations to this day) where they discuss the generic properties of systems of equations of the form:

u_t = D1*Delta u +
f(u,v)
v_t = D2*Delta v +
g(u,v),

which includes Eqs. 1-2 of this manuscript. A vast literature exists studying this type of models, some of them very close to the model proposed. However, a casual reader of the manuscript would not be aware of this. In line 166 the authors recognized that the phenomena in their model has been observed before, but the single reference to a low-profile recent work (43) does not convey the huge amount of work existent in this field. Another reference is made in the methods, (44), and together with some citations to founding papers of out-of-equilibrium studies, that is all. I understand that the goal here is not the review the field of pattern formation in excitable systems, but a clear presentation of the background from which this model is formulated should be given.

As mentioned above, we fully agree with the reviewer about the necessity for a more comprehensive discussion of seminal theoretical works in the field. A pivotal reference in our manuscript is Cross and Hoehenberg's "Pattern Formation outside of Equilibrium" from 1993 (http://doi.org/10.1103/RevModPhys.65.851), which is among the most cited in the field and also describes the Swift-Hohenberg model, related to the Ginzburg-Landau equation. We have now cited this work in the Introduction (lines 145-149) and Results sections (lines 334-337), as well as in supplementary Section S3.

Furthermore, we acknowledge the reviewer's comment about the need for a more extensive discussion about classical reaction-diffusion models. In response, we highlighted in the Result section that the diffusion-driven excitable behavior of the Sevilletor is not a Turing instability (lines 238-239), also discussed in supplementary Section S3 and shown by the dispersion relations in Figure S2. Regarding the work of Alan Turing, in lines 297-301 we state that the Sevilletor lateral inhibition behavior is equivalent to the “stationary waves of extreme short wave-length” in Turing 1952 (https://doi.org/10.1098/rstb.1952.0012). Additionally, in the Result section lines 195-202 and 221-239, the Discussion section lines 673-681 and supplementary Section S3 (Figures S3-S5) we now discuss in more detail the difference between our model and previous excitable reaction-diffusion models such as the Fitzhugh Nagumo Model, Brusselator and Oregonator.

This discussion is particularly pertinent concerning the Fitzhugh Nagumo model, previously used in Hubaud et al. 2017 (http://dx.doi.org/10.1016/j.cell.2017.08.043) to investigate the excitable behavior of the mouse PSM. In the discussion lines 673-681, we now explain that in Hubaud et al. 2017, the excitable behavior of the PSM was explored using a single-cell FitzHugh-Nagumo model, where the transition from quiescent to oscillatory states was simulated by adjusting the magnitude of a stimulus that excites a single stable steady state. In contrast, in the CWS model, this transition emerges from a bi-stable regime with three unstable states, where cells are excited to oscillate in high density cultures due to a relatively strong contribution of diffusion predicted by a spatially explicit model, as shown in Figure 5A. Finally, we mention that another unique property of the Sevilletor equations is that the distance between the unstable and stable points in the phase portrait determines the excitability, as illustrated in Figure 5E.

The authors have chosen to call “models” the different dynamical regimes that the Sevilletor equations can reach when certain values of the parameters or their spatial dependence are implemented. This can be somehow confusing, specially since specific implementations of this “models” studied in the manuscript do not cover the whole space of phenomena that the family of published works related to these regimes can show, nor can some of the conclusions extracted here from Sevilletor simulations be generalized so easily as sometimes implied. Also, the

© 2024. Published by The Company of Biologists under the terms of the Creative Commons Attribution License (https://creativecommons.org/licenses/by/4.0/).

intention of extending segmentation clock models to describe latest experimental developments like in vitro explants and somitoids is commendable, but the tone (previous efforts being wrong, when in fact they were proposed for more restricted settings; observations of the model being firsts, when references to the rich physics literature studying these kinds of dynamical systems are missing) can definitely be improved. Seasoned theoreticians familiar with segmentation clock models and a broad knowledge of dynamical systems would be able to see through this tone what is really going on, a real danger exists of misleading experimentalists not so familiar with technical aspects. With a little editing here and there, this issue can be easily corrected.

We thank the reviewer for providing us with this valuable feedback that we have used to improve the manuscript. We now make sure to refer to the four different models as the Sevilletor implementations of the CW/PORD/CG/CWS models throughout the manuscript to make sure that it is clear that these are phenomenological recreations of previous somitogenesis models, rather than perfect reincarnations. Importantly, we make clear that we don't want to compare models as mutually exclusive possibilities, favoring one model in an absolute manner against another.

Instead, as we say in the Results (lines 2454-251), our goal is to compare the qualitative patterning behaviors of different somitogenesis models by exploiting the dynamical patterning regimes exhibited by the Sevilletor equations. Specifically, our objective is to capture the qualitative aspects that control the emergence of oscillations, phase waves, and the arrest of oscillations in different models." We also briefly repeat this in the Introduction and Discussion to make sure it is clear to all readers (lines 161-164 and 627-630).

In this respect, our discussion and prediction in explant simulations reflect only the broad, high-level qualitative behavior of the models rather than quantitative details. We have also double-checked that it is clear, as this was the intention in our previous version of the manuscript as well, that the majority of explant simulations of the CG model are also able to recapitulate experimental data (lines 602-610). Related to this, we have now considered that graded signals such as FGF can be re-established in the explants (lines 576-581 and supplementary Section S20) for the PORD and CG explants in Figure 5H,J to the left. This new option replaces the previous simulation with an "inherited frequency gradient FG" (previous version of the manuscript: Figure 5H,I top rows), which had some noise due to shuffling during the creation of the explants. The new explant simulation with a smooth re-established circular frequency gradient FG without noise can also create a target pattern in the CG model in agreement with experiments (current version of the manuscript: Figure 5J to the left).

We discuss that the main differences between the Sevilletor implementation of the CG and the CWS explant simulations are the dynamics that cause target patterns to be formed. For the CG model it can be a re-established frequency gradient or a prepattern of phase values (u and v) from the tail, while for the CWS model it can also be the prepattern of phase values from the tail, or a pacemaker that guides the waves in the excitable regime.

Regarding the explants of the Sevilletor implementation of the PORD model, our findings show that the virtual explants are unable to create target patterns due to the initial noise. To support this claim, we have added Figure S14 where we show what happens when noise is added to the original PORD equations in 1D and 2D, to demonstrate how both the Sevilletor implementation of the PORD model and the model by Cotterell et al. 2015 (https://doi.org/10.1016/j.cels.2015.10.002) are sensitive to noise, consistent with studies by Pantoja-Hernández et al. 2021 (https://doi.org/10.1063/5.0045460).

We now make clear that the main novelty of the CWS with respect to previous models is its ability to recapitulate excitable dynamics observed experimentally in-vitro, such as density-dependent oscillations, Notch-dependent oscillation excitation thresholds, possible modulation induced by YAP signaling, and a newly added prediction: that in pairs of dissociated neighboring cells, the oscillations between the neighbors will be out-of-phase with excitable behavior, as observed in Movie S8 in Hubaud et al. 2017 (https://doi.org/10.1016/j.cell.2017.08.043) (Figure S32). These behaviors can be recapitulated because of the specific excitable behavior of the CWS model.

Some more small comments:

© 2024. Published by The Company of Biologists under the terms of the Creative Commons Attribution License (https://creativecommons.org/licenses/by/4.0/).

Line 97: "In essence, in the CG model, both the arrest of oscillations and the formation of phase waves are under the control of global signals".

This is a subtle point, as discussed in most of the references that propose this model. In them, a tissue-wide frequency profile is proposed, true, but the origin of this profile is not a part of the model. Global signals, e.g., signaling gradients are usually discussed, but other possibilities, as self-organization, are also consistent with these theoretical formulations. Note that these models were formulated for the geometry of the PSM, and were never intended to discuss in vitro or organoid experiments. Efforts tackling these new systems, as the manuscript under consideration, are necessary.

We thank the reviewer for bringing up this interesting point. We have reformulated this idea to: "One possibility is that the tissue-wide frequency profile is regulated by posterior signals, …" (lines 99-102), and we make sure not to draw definitive conclusions as to the origin of the frequency modulation for any of the models. Instead we refer to a specific example of how a global gradient has been thought to control the frequency in Kuyyamudi et al. 2022 (https://doi.org/10.1088/1478-3975/ac31a3): "... for example by modulating the local coupling-strength of delta-notch." (lines 99-102). We also clarified this point in the Discussion (line 643-649).

The time derivatives in Eqs. 1-2 should be partial derivatives, at least if the Laplacian has to be read in the continuous sense.

We have changed the equations in the paper correspondingly and we thank the reviewer for reminding us.

The value D=0.3 is used, but only mentioned in the methods. It would be useful to mention it also in the caption of Figure 2, where values are given for other parameters. The sizes of squares in this figure and Figure 3 are given in methods, but it would also be helpful to have them in the captions.

We have now added Tables 1-7 in the Methods Section with all the parameters used in every simulation presented in the Manuscript and the Supplementary. We now write the values of D and Lx, Ly as well as the most relevant parameters in the Figure captions and refer to the tables for the complete list of parameters used in each simulation.

Lines 2356 and 2357 in Methods: there is a typo. u(x,y,t+1) should be u(x,y,t+dt), and v(x,y,t+1) should be v(x,y,t+dt). It would also be nice to say here that the values of dt used at particular simulations will be given below.

We thank the reviewer for drawing our attention to the error that we have now corrected in equations 10-11. We refer to the newly added Tables 1-7 with all parameters so the readers can easily find the values used for dt for each simulation.

Parameters: if the time step used for the somitogenesis models is the same, dt=0.002, why is the frequency of adding a new row of cells during the somitogenesis simulation in a virtual tail different for each model?

The reason for varying the frequency of adding new posterior rows between the CG and CWS models comes from the different oscillation periods in the different models. The faster the oscillation is, the faster we set the growth of the tail, in order to create a somite pattern where the number of cells in the somites matches experimental findings (see supplementary Section S22). The oscillations in the CG model are faster than the ones in the CWS model, so the tail grows faster. For the PORD model, the growth of the tail has been set to match the speed of establishment of somites, as this is a self-organizing process that happens with a particular speed with the set of parameters used.

Line 404: "However, applying noise to the posterior gradient PG causes somite pattern disorganization over time (Figure 4J and Figure S9)". The "noise" in different models is difficult to compare. According to the supplement, 0.5% noise is added to the parameters k1, k3, and k4

© 2024. Published by The Company of Biologists under the terms of the Creative Commons Attribution License (https://creativecommons.org/licenses/by/4.0/).

every time a new row of cells is added on the posterior side. But in absolute value, this implies wider differences for model implementations that use higher values of the constants, and therefore not only the results of noise, but what could be considered comparable noise situations, is very subtle. I recommend a more nuanced and careful discussion.

The reviewer is right and indeed the fluctuations from the original values along the frequency gradient FG (previously called posterior gradient PG) profile will be higher the higher the value of FG is. We have now removed all references to “the same amount of noise”, that are now reformulated to “comparable amounts of noise” with a more detailed discussion in the new Methods section called “Noise”.

Indeed, determining the most comparable type of noise to parameters is inherently challenging and somewhat subjective. However, our approach benefits from using identical parameters for the posterior oscillatory and the anterior bistable regions across all models (k1=1 and k1=4, respectively for CW/CG/CWS), ensuring a consistent level of multiplicative noise added to k1. For k3 and k4, we set a minimum value of k3=k4=1 in all models. Given that in both the PORD and CG models, it is the relative difference in frequency that governs phase wave formation, we opted for relative multiplicative noise. This decision accounts for the differences in the maximum posterior values of k3 and k4, which are k3=k4=1.3 in the PORD and k3=k4=3 in the CG model. Note that in Figure 4E,H,G,K the maximum of the k3, k4 gradient has been rescaled for visualization. Interestingly, this adjustment seemingly favors the PORD model with lower absolute noise levels, yet this model emerges as the more fragile. Furthermore, noise is introduced each time that a new row of cells is added to the model ensuring normalization with respect to developmental time. Again, despite the slower growth rate of the PORD model, resulting in less absolute noise per unit time, it remains the more fragile model. Additionally, to ensure an equal accumulation of noise in parameters k1/k3/k4 across all cell rows, noise is added to complete parameter arrays (x=1 to L(t=final)) throughout the simulation. This approach ensures that the amount of noise does not diminish as cells progress anteriorly, even in regions not yet visible in simulations. This is discussed in the method section called “Noise”. Finally, it is important to note that we observed identical results to Figure 4G,J,M also when adding noise with a fixed amplitude instead of multiplicative (data not shown).

Therefore, although we are using multiplicative noise for parameters, we are confident that noise levels should be comparable for studying qualitative patterning behaviors. Additionally, in the main text and captions, we avoid direct robustness comparisons between models. Instead, we present the predictions of each model as independent observations of their qualitative behavior in noisy conditions.

We have also added the following simulations to provide a more detailed analysis of how noise and perturbations affect the Sevilletor implementations of the three models:

- PORD model:
  To support the argument that the PORD model is not robust to noise we have added the new Figure S14 that shows that both the original PORD equations by Cotterell et al. 2015 (https://doi.org/10.1016/j.cels.2015.10.002) modeled in 1D and 2D, and the Sevilletor implementation of the PORD model, are sensitive to noise, consistent with studies by Pantoja-Hernández et al. 2021 (https://doi.org/10.1063/5.0045460) (lines 316-323).
- CG model:
  We have added simulations of the CG and CWS models that include movement of cells (Figure S17), which illustrates that the Sevilletor implementation of the CG model is generally robust to perturbations in the phase values, further strengthening our hypothesis that it is in fact the noise in the frequency gradient FG (k3/k4), rather than in the phase values (u and v), that eventually causes the disruption of the pattern in Figure 4J and Movie 7 (lines 347-354).
- CWS model:
  We have added a new Figure S16 where we show the effect of adding noise with temporal fluctuations in the scaling of the noise in the CWS model to show that it is robust to form phase waves even when bursts of noise appear, and it is robust to include movement of cells (Figure S17) (lines 406-416).

© 2024. Published by The Company of Biologists under the terms of the Creative Commons Attribution License (https://creativecommons.org/licenses/by/4.0/).

The details are provided in the section called "Noise" in the Methods Section. We also provide all parameters used for the simulations in the new Tables 1-7 for complete transparency.

Line 406: "Finally, the model predicts a loss of phase waves in mutants with posterior signal expansion and flattening, contrary to experiments [3] (Figure 4I)". This is a misleading representation of what the Clock and Gradient model can do. Reference [3] studies mutant beta-catenindel(ex3)/+ mice embryos, the conclusion, expressed in the title, is that a beta-catenin gradient links the clock and wavefront systems in mouse embryo segmentation. Certainly then, in the framework of the Clock and Gradient model, the effect of the beta-catenindel(ex3)/+ mutation would not be limited to an expansion of the PSM: a change of the shape of the gradient would be certainly expected. These are phenomenological models, and therefore it is difficult to find phenomena that they cannot reproduce. The simulation of figure 4I shows an expanded PSM with a very flat gradient, abruptly decaying in the anterior PSM. A gradual decay from posterior to anterior would reproduce the phase wave observed in the experiments, scheme in Figure 3i of [3].

We very much appreciate the reviewer's comments regarding the way the different models were compared in the previous version of the manuscript and we agree that a more extensive discussion and comprehensive study of the CG model was needed. Firstly, we relocated the simulations with extended posterior gradients to the supplementary section (Figure S21), where we now perform a more complete sweep of perturbations of R and FG (frequency gradient, previously "PG") in the CG and CWS model. Here, we speculate that the most direct comparison of the extended gradients of Pea3 (FGF signaling) and Axin2 (WNT signaling) observed in Aulehla et al. (2008) would be the case where R and FG are extended anteriorly, which in the Sevilletor implementation of the CG model shows a loss of phase waves (Figure S21D). However, we also show that under different perturbations of R and FG (where R is expanded anteriorly and FG is left unchanged), the CG model can also give rise to multiple phase waves (Figure S21H). We also show that the CG creates phase waves with a linearly decreasing FG (Figure S21I), and that the CG does not form waves with a flat FG (Figure S21J). This highlights a key feature of the qualitative behavior of Clock and Gradient (CG) models, as the name suggests, which is that a frequency gradient is needed for the formation of phase waves. However it is not needed for establishing the somite pattern. In the CWS model the phase waves are created due to the excitable behavior, so phase waves as well as somite formation is not dependent upon a frequency gradient.

Importantly, we now removed the statements that said that the CG model predicts loss of phase waves, and we have clarified that the main aim of our study is not to favor one model over another, but rather to employ the Sevilletor equations to illustrate how the main dynamical patterning regimes of the models can replicate distinct qualitative behaviors of the PSM (lines 245-251). Specifically, we emphasize that the excitable regime of the CWS model can recapitulate the behavior of the mouse PSM observed in vitro. In addition, we mention that the features of the CG and CWS models are not mutually exclusive, now in the Discussion section (lines 627-630), and we do not exclude that other versions of the models can recapitulate features that are not included in the Sevilletor implementations.

Regarding this mistake in model interpretation, I would like to stress again that a wonderful thing about the model proposed is its power to reproduce different mechanisms in a single framework, but with great power comes great responsibility, and care has to be taken before reaching conclusions.

We thank the reviewer for the awesome quote and indeed we have used it to significantly improve the manuscript with a more nuanced approach to discuss possible conclusions and make comparisons.

Line 482: "providing a possible explanation for the multiple phase waves observed upon expansion and flattening posterior gradients in mouse mutants (Figure 4L, Movie 8) [3]". This is true, and nice, but recall the previous comment that other model formulations can also reproduce this observation, and that it is because a flawed formulation that this was not recapitulated in the manuscript.

© 2024. Published by The Company of Biologists under the terms of the Creative Commons Attribution License (https://creativecommons.org/licenses/by/4.0/).

In our current improved version of the manuscript we make sure to show how different perturbations of the Sevilletor implementation of the CG model affects the dynamics to either create more or less phase waves (details in the previous comment) (Figure S21). We now state that an interesting feature of the CWS model is that the excitable regime can create many phase waves without the need for a frequency gradient, without drawing conclusions to directly compare which model corresponds best to experiments.

Line 495: "the somite pattern formed by the CWS is also robust to noise applied to parameter values. Indeed, the model can be implemented with any values of $0 < k1 \leq 2.3$ for the posterior oscillatory region and any values of $k1 > 4$ in the anterior region". As discussed above, I believe this comparison is not straightforward, and recommend a more nuanced discussion. Note also "same amount of noise" in line 502.

This sentence has been removed in the improvement and shortening of the manuscript, and all references to "the same amount of noise" have also been reformulated to "comparable amounts of noise" with a more nuanced and detailed discussion of the result of adding noise to different parameter values.

Typo: Ref. 21 is incomplete.

We have corrected this reference.

References

Aranson, I. S., & Kramer, L. (2002). The world of the complex Ginzburg-Landau equation. Reviews of modern physics, 74(1), 99.
Tyson, J. J., & Keener, J. P. (1988). Singular perturbation theory of traveling waves in excitable media (a review). Physica D: Nonlinear Phenomena, 32(3), 327-361.

We thank the reviewer for providing these references which we now discuss in the supplementary Section S3, and that we have added to the Introduction (lines 145-149) and Results (lines 221-239) sections of the manuscript.

We are very grateful to Reviewer 3 for the careful considerations made in reviewing our manuscript and all the comments that have allowed us to improve our manuscript to be more well-balanced and considerate towards the discussion of previous models of somitogenesis and also dynamical systems theory.

Second decision letter

MS ID#: DEVELOP/2023/202606

MS TITLE: A Clock and Wavefront Self-Organizing Model Recreates the Dynamics of Mouse Somitogenesis in-vivo and in-vitro

AUTHORS: Julie Klepstad and Luciano Marcon
ARTICLE TYPE: Research Article

I am happy to tell you that your manuscript has been accepted for publication in Development, pending our standard ethics checks.

Reviewer 1

Advance summary and potential significance to field

© 2024. Published by The Company of Biologists under the terms of the Creative Commons Attribution License (https://creativecommons.org/licenses/by/4.0/).

The authors of this excellent manuscript have done a fantastic job in this revision. The final piece is ready for publication in my opinion, and addresses well the comments posed by myself and my co-referees. The work is extremely thorough on the theory side, and is very readable/understandable for experimentalists. It will be a fantastic resource to inspire future experiments and theory alike in the coming years. The approach used here — taking a common set of equations and exploring how alternative spatiotemporal patterns of parameters influence emergent behaviour differentially among alternative conceptual models/mechanisms — is something that more theorists studying development should aspire towards.

Comments for the author

I have no further comments. The authors have gone above and beyond the expected in answering my set of questions in my first review.

Reviewer 2

Advance summary and potential significance to field

The authors have addressed all my comments appropriately and - in my opinion - also those of the other reviewers. This has improved the manuscript and I fully support its publication.

Comments for the author

I don't have any further suggestions to add at this stage.

Reviewer 3

Advance summary and potential significance to field

In this new version, the authors have made a very thorough effort to address the comments of the reviewers and follow their suggestions. As a result, the new version of the manuscript is much more successful in putting the work into context, and more rigorous in its details. I think that the process has been constructive by all parts, improving the work, that now I recommend accepting.

Comments for the author

Regarding the responses to my comments in particular, I am satisfied with the effort done by the authors.

© 2024. Published by The Company of Biologists under the terms of the Creative Commons Attribution License (https://creativecommons.org/licenses/by/4.0/).
